# Inhibition of calpain-mediated HMGB1 alleviates cardiac inflammation and dysfunction induced by ultra-processed foods

**DOI:** 10.1172/jci.insight.199622

**Published:** 2026-04-09

**Authors:** Claire Ross, Sanskruti Ravindra Gare, Nasser H.O. Alatawi, Oveena Fonseka, Xinyi Chen, Jiayan Zhang, Yihua Han, Andrea Ruiz-Velasco, Riham R.E. Abouleisa, Yingjuan Liu, Xiangjun Zhao, Han Xiao, Bernard D. Keavney, Gareth J. Howell, Tao Wang, Tamer M.A. Mohamed, Elizabeth J. Cartwright, Wei Liu

**Affiliations:** 1 Faculty of Biology, Medicine and Health, The University of Manchester, Manchester, United Kingdom.; 2 Surgery Department, Baylor College of Medicine, Houston, Texas, USA.; 3 Department of Cardiology and Institute of Vascular Medicine, Peking University Third Hospital, Beijing, China.; 4 Manchester Heart Centre, Manchester University Hospitals NHS Foundation Trust, Manchester Academic Health Science Centre, Manchester, United Kingdom.

**Keywords:** Cardiology, Inflammation, Metabolism, Diabetes, Heart failure

## Abstract

Increased consumption of ultra-processed foods (UPFs) is a risk factor for metabolic disorder–associated heart failure (HF). Here, we demonstrate that UPF-induced calpain-1 aggravated oxidative stress, thereby increasing high mobility group box 1–mediated (HMGB1-mediated) myocardial inflammation, which contributes to cardiac dysfunction. After illustrating the dysregulated inflammatory pathways in human and murine hearts upon metabolic stress, we revealed an increase in calpain-1 alongside profound oxidative stress and inflammation in the failing myocardium. Mechanistically, in neonatal rat cardiomyocytes and human induced pluripotent stem cell–derived cardiomyocytes, HMGB1 was upregulated by calpain-1 and reactive oxygen species (ROS) upon stress of saturated and trans fatty acids. Consequently, HMGB1 promoted a proinflammatory response in macrophages. In contrast, inhibition of calpain or ROS efficiently repressed HMGB1 in cardiomyocytes. Therapeutically, either recombinant adeno-associated virus 9–delivered inhibitor of calpain-1 or its pharmacological inhibitor attenuated ROS and HMGB1-induced inflammation in the myocardium and mitigated HF in both male and female mice fed with an ultra-processed diet. Collectively, we have demonstrated the effects of suppressing calpain-1 and oxidative stress on alleviating myocardial inflammation via blockage of HMGB1 and cardiac dysfunction. The results provide a promising therapeutic strategy for preventing or treating HF in metabolic disorders.

## Abstract 2

Cardiac CAST protects against diet-induced heart dysfunction and remodeling, suggesting a potential therapy for cardiometabolic heart disease.

## Introduction

Heart failure (HF) is one of the most prevalent causes of hospital admission and has a poor prognosis in diabetes, despite breakthroughs in treatment strategies (1). The diabetic heart is characterized by abnormal cardiac structure and impaired performance in the absence of other cardiac risk factors (2). Limited knowledge of the molecular mechanisms underlying HF in diabetes restricts our efforts to identify specific treatments (3, 4). Thus, it is imperative to decipher the molecular pathogenesis, which will provide potential avenues to discover potential therapeutic strategies.

Recent studies report that high consumption of ultra-processed foods (UPFs) accounts for more than 10% of the diet proportion in daily life (5), which is an undeniable risk factor for adverse health outcomes, including inflammation and cardiometabolic disorders (6, 7). Greater exposure to UPFs is closely associated with the increased prevalence of diabetes and subsequent HF in adults (8–10) but there is limited understanding of the biological processes at the molecular level and the related treatment targets.

Myocardial inflammatory responses have been considered as the underlying pathology of metabolic disorder–induced HF (11), while inhibition of cardiac inflammation mediates clinical benefits of HF therapies (12). High mobility group box 1 (HMGB1) is a non-histone chromosome-binding protein participating in gene regulation, whereas extracellular HMGB1 acts as a danger-associated molecule pattern that triggers inflammation (13). Of note, cardiomyocyte-sourced HMGB1 contributes to the development of HF in diabetes (14), providing a rationale for the therapeutic potential of repressing HMGB1 secretion, rather than completely reducing its levels in the myocardium. Under oxidative stress, HMGB1 is prone to be oxidized or acetylated (15, 16), and is thus transported from the nucleus to the cytoplasm and released extracellularly (17).

The calpain family are calcium-dependent neutral cysteine proteases playing roles in multiple cellular processes. Dysfunctional calpain contributes to the development of adverse cardiac remodeling and eventual HF under various pathological conditions, including metabolic stress (18, 19). In particular, calpain-1, as the predominant isoform expressed in cardiomyocytes (20), is a significant contributor to cardiac dysfunction due to inducing profound oxidative stress. For instance, calpain-1 is activated by oxidases to induce cardiomyocyte apoptosis (19, 21). In turn, elevated calpain-1 provokes reactive oxidative species (ROS) generation (22, 23). In contrast, inhibition of calpain-1 ameliorates oxidative stress–triggered cardiac dysfunction (20, 24). It has also been demonstrated that activated calpain-1 triggers cardiac inflammation and myocarditis (25). Additionally, it is positively correlated with activation of proinflammatory pathways in various immune cells (26). However, the effects of calpain regulation on myocardial inflammation under metabolic stress induced by UPFs have never been investigated to the best of our knowledge.

In this study, using preclinical models, we decipher the molecular mechanism whereby calpain-1 promoted oxidative stress in the heart in response to ultra-processed diet (UPD) leads to higher levels of HMGB1 and cardiac inflammation. Firstly, a synchronous increase in calpain-1, oxidative stress, and HMGB1 was detected in diabetic failing human hearts, accompanied by augmented transcripts of proinflammatory cytokines. Next, metabolic stress was induced by UPD feeding in both male and female mice, manifesting a similar cardiac phenomenon. Additionally, blockage of ROS by an antioxidant, *N*-acetylcysteine (NAC), prevented cytosolic HMGB1 increases in rat cardiomyocytes and human induced pluripotent stem cell–derived cardiomyocytes (hiPSC-CMs) upon a combined stress of palmitic acid (PA), elaidic acid (EA), and linoelaidic acid (LA). Importantly, functional evidence was obtained showing that both overexpression of an endogenous inhibitor of calpain and the pharmacological inhibition of calpain reduced oxidation stress and alleviated HMGB1 levels in the myocardium, thereby preventing cardiac inflammation and dysfunction induced by UPD.

## Results

### Calpain-1 and HMGB1 are associated with cardiac inflammation in HF.

**Figure 1:**
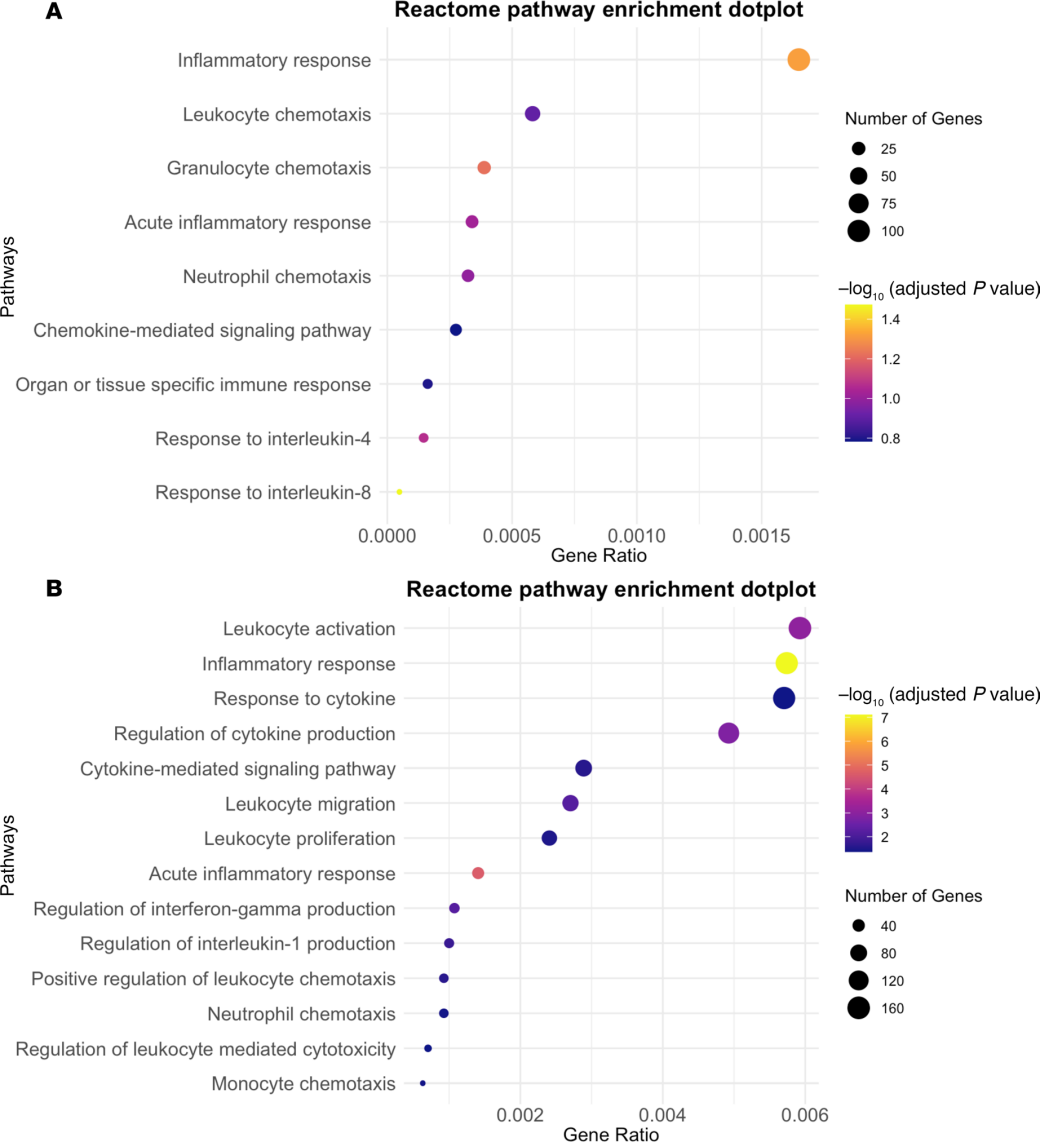
Inflammation is increased in diabetes-associated heart failure (HF). (**A** and **B**) GO cellular biological process analysis of differentially expressed genes in human HF versus normal hearts (*n* = 4–12) (305 genes with absolute fold change greater than 1) (**A**) and hearts obtained from mice fed a 25-week high-fat diet (HFD) or chow diet (*n* = 6) (998 genes with absolute fold change greater than 1) (**B**).

**Figure 2:**
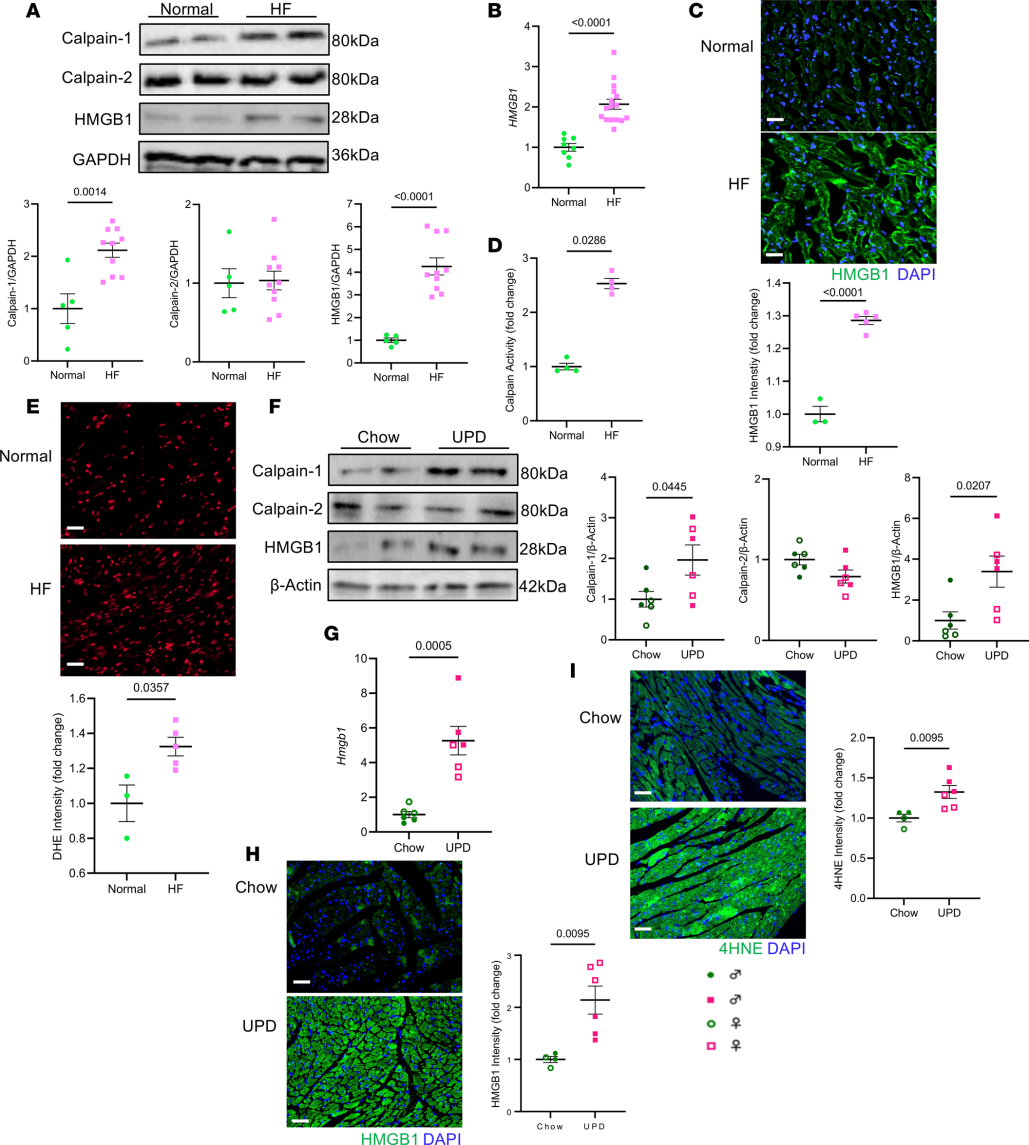
Oxidative stress is associated with increased cardiac HMGB1 and calpain-1 upon ultra-processed diet (UPD). (**A**) Representative immunoblots and quantification demonstrating cytosolic HMGB1, calpain-1, and calpain-2 expression in human HF hearts, where GAPDH was used as a loading control (*n* = 5–10 hearts). (**B**) qPCR of *HMGB1* (*n* = 8–16 hearts). (**C**) Representative images and quantification of cardiac HMGB1 (scale bars: 20 μm) in human HF hearts (*n* = 3–5 hearts). (**D**) Calpain activity assay in human HF versus normal hearts (*n* = 4 hearts). (**E**) Representative images and quantification of cardiac DHE (scale bars: 50 μm) in human HF hearts (*n* = 3–5 hearts). (**F**) Representative immunoblots and quantification demonstrating HMGB1, calpain-1, and calpain-2 expression in murine hearts following 12 weeks of UPD or chow diet, where β-actin was used as a loading control (*n* = 6 hearts). (**G**) qPCR of *Hmgb1* (*n* = 6 hearts). (**H** and **I**) Representative images and quantification of cardiac HMGB1 (**H**) (scale bars: 20 μm) and 4HNE (**I**) (scale bars: 50 μm) in murine hearts following 12 weeks of UPD or chow diet (*n* = 4–6 hearts). Data are presented as mean ± SEM, with solid and hollow symbols representing male and female mice, respectively (**F**–**I**). *P* values were calculated using 2-tailed Student’s *t* tests (**A**, **B**, **F**, and **G**) and Mann-Whitney tests (**C**–**E**, **H**, and **I**).

RNA sequencing was conducted on failing heart samples obtained from humans with metabolic disorders to assess cardiac inflammation in metabolic stress–associated HF. Pathway analysis using ShinyGO 0.82 (Supplemental Methods; supplemental material available online with this article; https://doi.org/10.1172/jci.insight.199622DS1) revealed significantly altered inflammatory pathways (Figure 1A). A similar phenomenon was observed in the hearts of mice fed with a high-fat diet (HFD, 60% calorie from fat) for 25 weeks (Figure 1B). As an important trigger of the inflammatory response, HMGB1 was increased in the cytosol of failing human hearts (Figure 2, A–C). Since calpain-1 impairs the redox state intracellularly, the synchronous augmented levels of calpain-1 expression and activity, and ROS, as indicated by dihydroethidium (DHE) staining, were observed (Figure 2, A, D, and E).

Comparing preclinical models of metabolic disorders induced by various diets, mice fed with a UPD were more susceptible to both diastolic and systolic dysfunction within the same time frame (Supplemental Figure 1 and Supplemental Table 1). UPD promoted cardiac inflammation, firstly evidenced by the higher levels of proinflammatory genes (*Tnfa*, *Il1b*, *Il6*, and *Crp*) but lower levels of antiinflammatory genes (*Arg1*, *Il10*, and *Gdf15*) in the myocardium (Supplemental Figure 2, A and B). Of note, calpain-1 (but not calpain-2) and HMGB1 were increased significantly in the hearts of mice fed with a UPD (Figure 2, F–H), which was associated with oxidative stress as indicated by elevated 4-hydroxynonenal (4HNE) expression, which itself is generated in response to ROS (Figure 2I) and an increase in the expression of antioxidant (*Sod2* and *Gpx1*) genes (Supplemental Figure 2C). These were strongly linked to a profound rise in pathological cardiac remodeling markers (*Nppb*, *Col1a2*, and *Col3a1*) (Supplemental Figure 2D). Noticeably, recombinant calpain-1 protein increased intracellular ROS and cytosolic HMGB1 levels, concurrently with confirmed overexpression of calpain-1 protein, in both neonatal rat cardiomyocytes (NRCMs) and hiPSC-CMs (Supplemental Figure 3, A–F). Furthermore, overexpression of calpain-1 in NRCMs similarly correlated with elevation in ROS and cytosolic HMGB1 levels (Supplemental Figure 4, A–C). These data indicate the possible relationship between the cascade of calpain-1, ROS, HMGB1, and cardiac inflammation in UPD-induced metabolic disorder.

### Cardiac CAST overexpression ameliorates UPD-induced cardiac dysfunction and oxidative stress.

**Figure 3:**
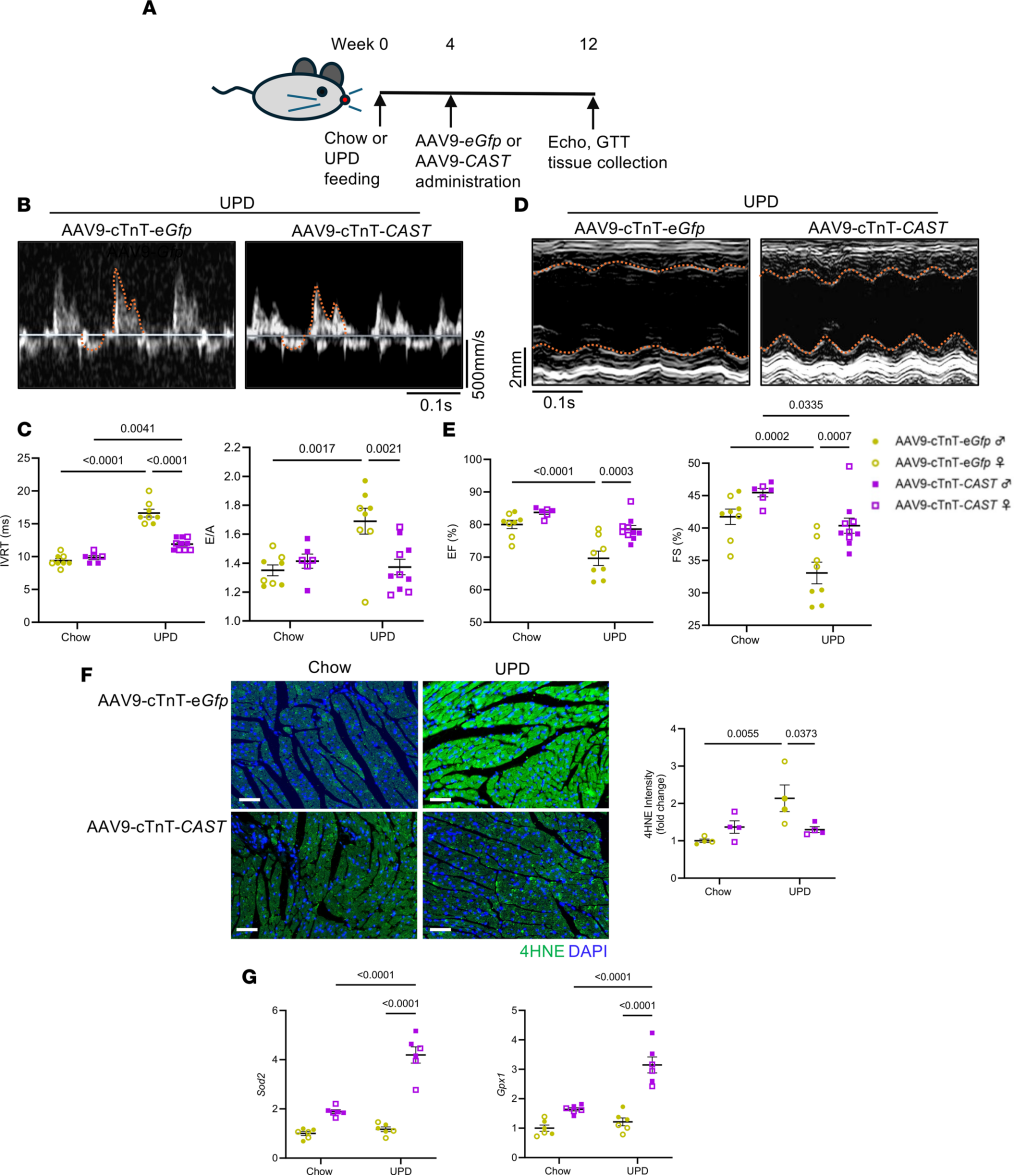
Cardiac-specific calpain inhibition reduces ultra-processed diet–induced cardiac dysfunction and oxidative stress in both sexes of mice. (**A**) Schematic of the experimental design. (**B**) Representative pulsed-wave Doppler tracings. (**C**) Isovolumic relaxation time (IVRT) and ratio of peak velocity blood flow from left ventricular relaxation in early diastole to that in late diastole (E/A) (*n* = 6–10 mice). (**D**) Representative left ventricular M-mode echocardiographic tracings in short-axis view. (**E**) Percentage of ejection fraction (EF%) and fractional shortening (FS%) (*n* = 6–10 mice). (**F**) Representative images and quantification of cardiac 4HNE staining (green) with DAPI-stained nuclei (blue) (scale bars: 50 μm) (*n* = 4 hearts). (**G**) qPCR of antioxidant genes *Sod2* and *Gpx1* (*n* = 6 hearts). Data are presented as mean ± SEM, with solid and hollow symbols representing male and female mice respectively. *P* values were calculated using 2-way ANOVA with Šidák’s post hoc test (**C** and **E**–**G**). UPD, ultra-processed diet.

To test the hypothesis that calpain-1 initiates the harmful effects, we assessed whether the specific inhibition of cardiac calpain-1 in the myocardium ameliorates the detrimental effects of UPD-induced cardiac dysfunction. Calpastatin (CAST) is an endogenous protein that inhibits the activity of calpain (27), including the most predominant member, calpain-1, that was increased in the failing heart (Figure 2, A and F). Thus, adeno-associated virus 9 carrying the human *CAST* cDNA sequence driven by the cardiac troponin T (cTnT) promoter (AAV9-cTnT-*CAST*), which achieved cardiac overexpression of CAST (Supplemental Figure 5, A and B), was used to block calpain-1, as indicated by a significant reduction in cardiac calpain activity (Supplemental Figure 6). Since mice fed with UPD exhibited a significant decrease in diastolic function 4 weeks after feeding (Supplemental Table 2), mice were intravenously injected with AAV9-cTnT-*CAST* after 4 weeks (Figure 3A) to determine whether cardiac dysfunction can be treated by blockage of calpain activity. Firstly, UPD-fed male and female mice receiving AAV9-cTnT-e*Gfp* displayed increased isovolumic relaxation time (IVRT), an increase in the ratio of early to late diastolic filling velocities of the left ventricle (E/A ratio), pulmonary edema indicated by higher lung weight to tibia length ratio (Figure 3, B and C, and Supplemental Table 3), and decreased percentages of ejection fraction (EF%) and fractional shortening (FS%) (Figure 3, D and E, and Supplemental Table 3), validating UPD-induced cardiac dysfunction. However, mice with cardiac CAST overexpression exhibited significant improvement in both diastolic and systolic functional parameters in both sexes (Figure 3, B–E, and Supplemental Table 3).

Of note, metabolic disorder was induced by the UPD, evidenced by increased body weight and fasting blood glucose levels, and impaired glucose tolerance test, which was unaffected by cardiac CAST overexpression (Supplemental Figure 7, A–C), indicating that the improved cardiac performance is independent of systemic metabolic changes. Moreover, UPD contributed to pathological cardiac remodeling, evidenced by cardiomyocyte hypertrophic growth (Supplemental Figure 8, A and B), cardiac fibrosis (Supplemental Figure 8, A and C), and profound apoptosis in the myocardium (Supplemental Figure 8D). In contrast, the harmful effects of UPD were alleviated in cardiac CAST–overexpressing mice (Supplemental Figure 8, A–D). More importantly, UPD-induced oxidative stress in the myocardium was ameliorated by CAST overexpression (Figure 3F), accompanied by augmented levels of antioxidant genes, such as *Sod2* and *Gpx1* (Figure 3G). Taken together, these observations suggest that cardiac-specific calpain inhibition protects the heart against UPD-induced dysfunction.

### Cardiac CAST overexpression mitigates inflammation in the myocardium upon UPD feeding.

**Figure 4:**
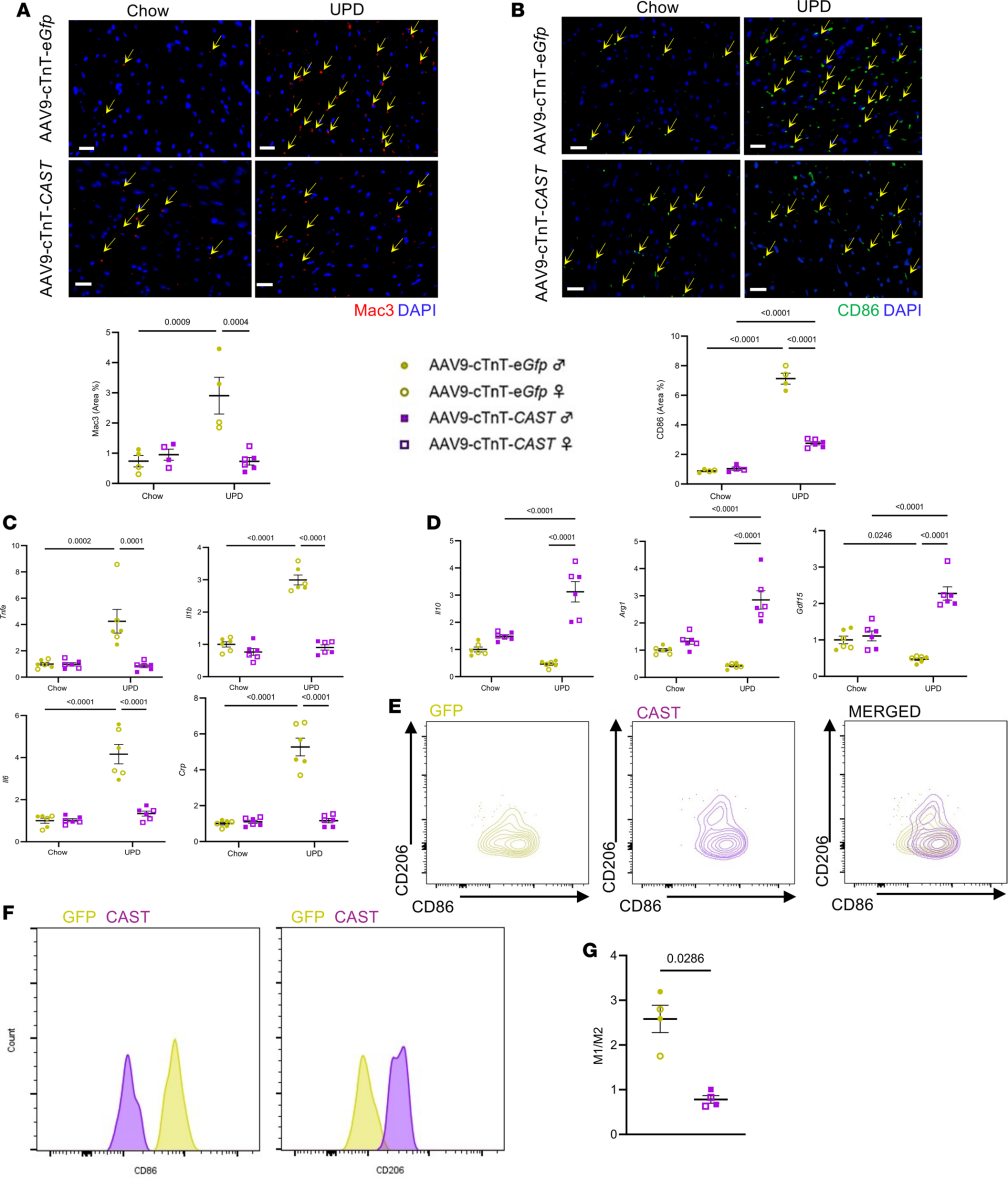
Cardiac-specific calpain inhibition reduces ultra-processed diet–induced cardiac inflammation. (**A**) Representative images and quantification of cardiac Mac3 staining (red, arrows) with DAPI-stained nuclei (blue) (scale bars: 20 μm) (*n* = 4–6 hearts). (**B**) Representative images and quantification of cardiac CD86 staining (green, arrows) with DAPI-stained nuclei (blue) (scale bars: 20 μm) (*n* = 4–6 hearts). (**C** and **D**) qPCR of proinflammatory (**C**) and antiinflammatory (**D**) genes. (**E**) Representative flow contour plots of CD86^+^ and CD206^+^ macrophages in the heart. (**F**) Representative histograms displaying CD86^+^ and CD206^+^ macrophage subtypes in the heart. (**G**) Quantification of the relative ratio of M1 and M2 macrophages (M1/M2) (*n* = 4 hearts). Data are presented as mean ± SEM, with solid and hollow symbols representing male and female mice, respectively. *P* values were calculated using 2-way ANOVA with Šidák’s post hoc test (**A**–**D**) and Mann-Whitney test (**G**). UPD, ultra-processed diet.

**Figure 5:**
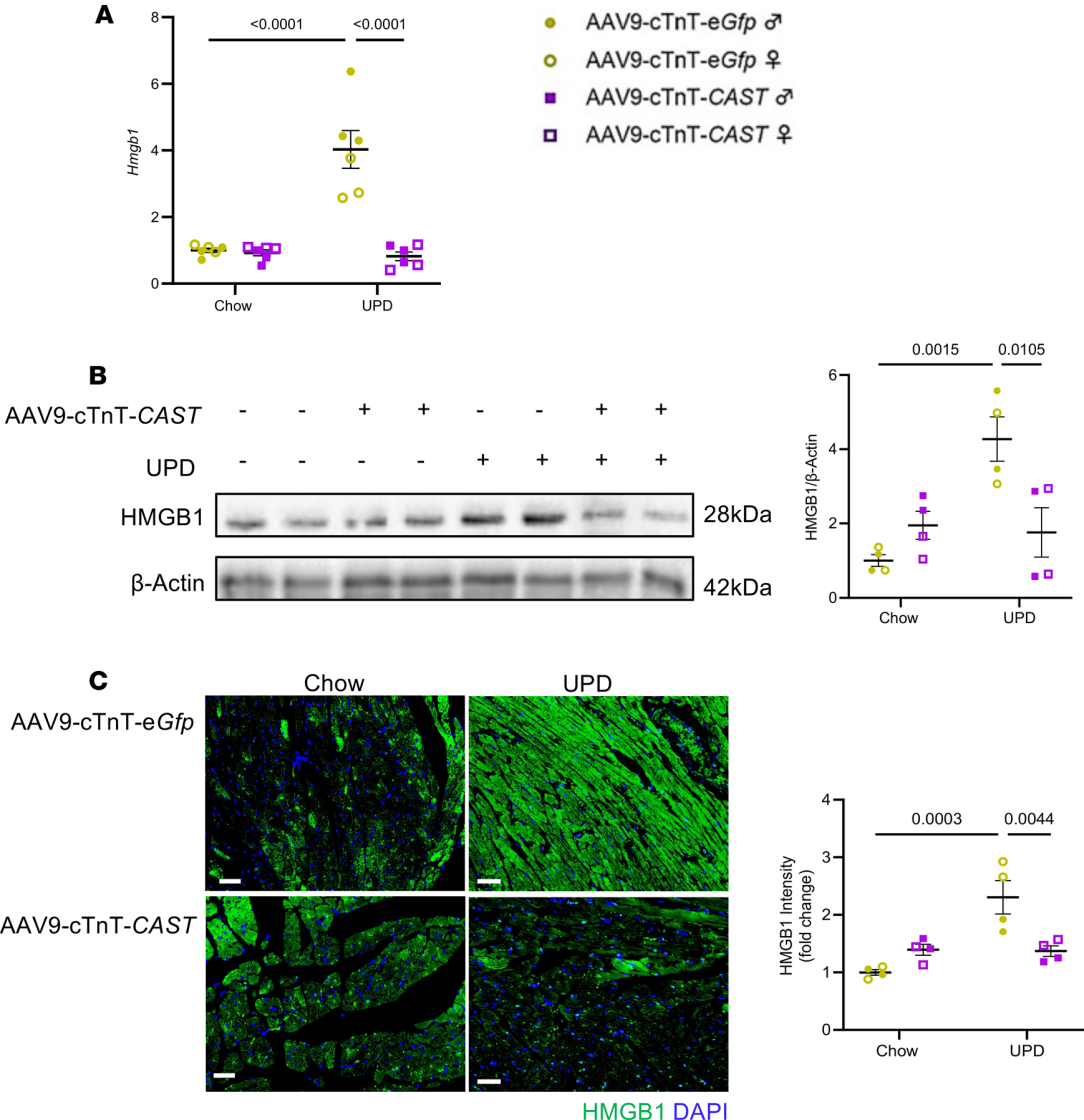
Cardiac-specific calpain inhibition decreases cardiac HMGB1. (**A**) qPCR of *Hmgb1* (*n* = 6 hearts). (**B**) Representative immunoblots and quantification demonstrating HMGB1 expression in response to ultra-processed diet (UPD) and CAST overexpression, where β-actin was used as a loading control (*n* = 4 hearts). (**C**) Representative images and quantification of cardiac HMGB1 staining (green) with DAPI-stained nuclei (blue) (scale bars: 50 μm) (*n* = 4 hearts). Data are presented as mean ± SEM, with solid and hollow symbols representing male and female mice, respectively. *P* values were calculated using 2-way ANOVA with Šidák’s post-hoc test (**A**–**C**).

Next, we assessed myocardial inflammation in the diseased state in the presence and absence of CAST overexpression. First, the number of macrophages increased in the hearts of mice fed with a UPD for 12 weeks, which was determined by the increased amount of Mac3^+^ cells (a general macrophage marker) (Figure 4A) and CD86^+^ cells (a specific M1 macrophage marker indicating proinflammation) (Figure 4B). Strikingly, the levels of both Mac3 and CD86 were significantly decreased in the hearts overexpressing CAST (Figure 4, A and B). Furthermore, the increased transcripts of proinflammatory genes, such as *Tnfa*, *Il1b*, *Il6*, and *Crp*, in the myocardium of mice fed with an UPD were all ameliorated by cardiac CAST overexpression (Figure 4C). Conversely, the expression of antiinflammatory genes, such as *Il10*, *Arg1*, and *Gdf15*, were augmented in UPD-fed mice by the inhibition of calpain-1 (Figure 4D). More convincingly, flow cytometry performed on isolated macrophages from the collected hearts demonstrated that there was a decrease in the proinflammatory M1 subtype (CD86^+^) macrophages in CAST-overexpressing mice in comparison with the untreated UPD-fed group (Supplemental Figure 9 and Figure 4, E–G). As expected, HMGB1 in the myocardium was promoted by UPD for 12 weeks (Figure 5, A–C); however, both its transcript (Figure 5A) and protein levels (Figure 5, B and C) were blunted by CAST overexpression (Figure 5, A–C). Collectively, we have demonstrated that cardiac CAST overexpression prevents oxidative stress and myocardial inflammation in UPD-induced cardiac dysfunction, at least partially resulting from downregulation of HMGB1.

### A pharmacological inhibitor of calpain-1 delivers beneficial effects on cardiac function.

**Figure 6:**
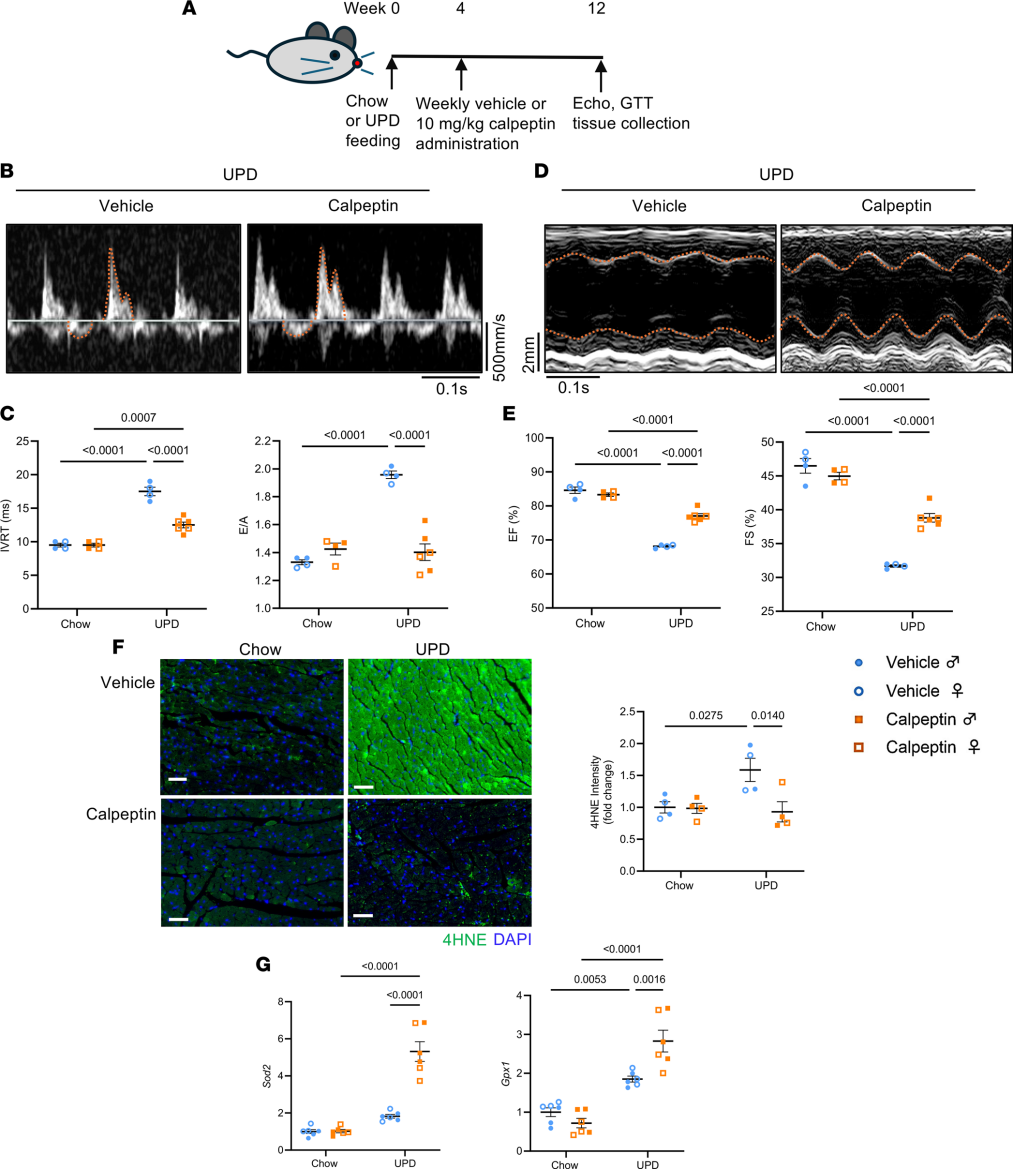
Pharmacological inhibition of calpain reduces ultra-processed diet–induced cardiac dysfunction and oxidative stress in both sexes of mice. (**A**) Schematic of the experimental design. (**B**) Representative pulsed-wave Doppler tracings. (**C**) Isovolumic relaxation time (IVRT) and ratio of peak velocity blood flow from left ventricular relaxation in early diastole to that in late diastole (E/A) (*n* = 4–6 mice). (**D**) Representative left ventricular M-mode echocardiographic tracings in short-axis view. (**E**) Percentage of ejection fraction (EF%) and fractional shortening (FS%) (*n* = 4–6 mice). (**F**) Representative images and quantification of cardiac 4HNE staining (green) with DAPI-stained nuclei (blue) (scale bars: 50 μm) (*n* = 4 hearts). (**G**) qPCR of antioxidant genes *Sod2* and *Gpx1* (*n* = 6 hearts). Data are presented as mean ± SEM, with solid and hollow symbols representing male and female mice, respectively. *P* values were calculated using 2-way ANOVA with Šidák’s post hoc test (**C** and **E**–**G**). UPD, ultra-processed diet.

To assess the potential benefits of calpain-1 inhibition for the treatment of UPD-induced HF, the pharmacological inhibitor of calpain family, calpeptin, was administered to mice under UPD stress. Following feeding with the UPD for 4 weeks, weekly intravenous injections of calpeptin (10 mg/kg) were performed for an additional 8 weeks (Figure 6A). Although systemic metabolic profiles were unchanged by calpeptin treatment, as shown by similar body weight, fasting blood glucose level, and glucose tolerance capability (Supplemental Figure 10, A–C), echocardiography assessment revealed that compared with UPD-fed mice that received a placebo (vehicle), calpeptin administration rescued diastolic dysfunction, as determined by reduced IVRT and E/A ratio in both male and female mice (Figure 6, B and C, and Supplemental Table 4). In addition, cardiac hypertrophy, indicated by the ratio of heart weight to tibia length, and the complementary systolic dysfunction were both ameliorated by weekly administration of calpeptin in both sexes fed a UPD (Figure 6, D and E, and Supplemental Table 4). Akin to CAST overexpression, pharmacological inhibition of calpain-1 also attenuated pathological cardiac remodeling, indicated by less hypertrophy (Supplemental Figure 11, A and B, and Supplemental Table 4), fibrosis (Supplemental Figure 11, A and C), and cell death (Supplemental Figure 11D). Furthermore, the observed changes in 4HNE expression determined that calpeptin treatment reduced oxidative stress in the myocardium following UPD feeding (Figure 6F). This was further evidenced by the higher levels of antioxidant genes, such as *Sod2* and *Gpx1*, due to calpeptin administration (Figure 6G). Pharmacological treatment–based data supported the protective action of calpain inhibition to tackle the metabolic stress provoked by UPD.

### Calpeptin administration ameliorates HMGB1-associated inflammation in the heart.

**Figure 7:**
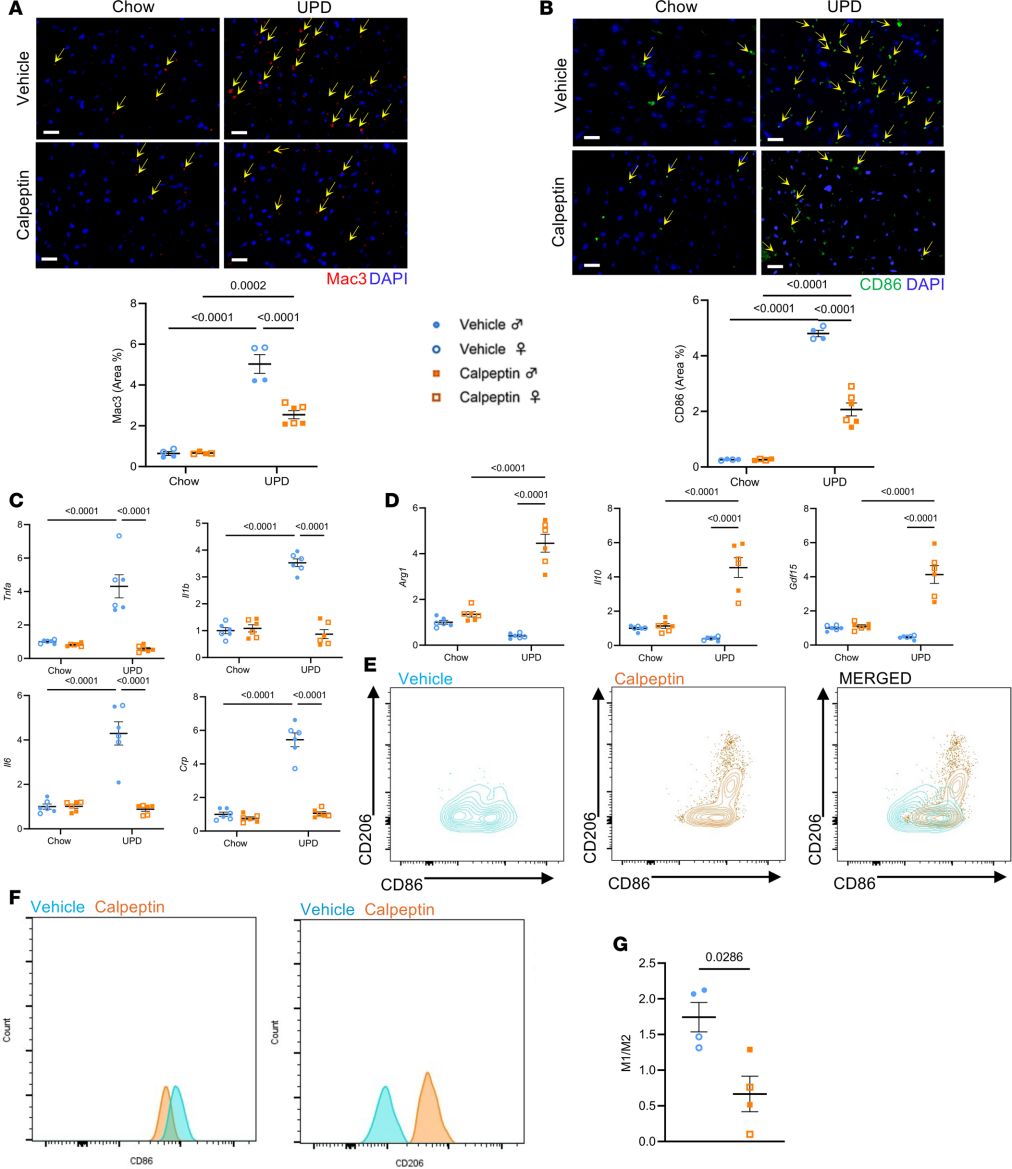
Pharmacological inhibition of calpain reduces ultra-processed diet–induced cardiac inflammation. (**A**) Representative images and quantification of cardiac Mac3 staining (red, arrows) with DAPI-stained nuclei (blue) (scale bars: 20 μm) (*n* = 4–6 hearts). (**B**) Representative images and quantification of cardiac CD86 staining (green, arrows) with DAPI-stained nuclei (blue) (scale bars: 20 μm) (*n* = 4–6 hearts). (**C** and **D**) qPCR of proinflammatory (**C**) and antiinflammatory (**D**) genes. (**E**) Representative flow contour plots of CD86^+^ and CD206^+^ macrophages in the heart. (**F**) Representative histograms displaying CD86^+^ and CD206^+^ macrophage subtypes in the heart. (**G**) Quantification of the relative ratio of M1 and M2 macrophages (M1/M2) (*n* = 4 hearts). Data are presented as mean ± SEM, with solid and hollow symbols representing male and female mice, respectively. *P* values were calculated using 2-way ANOVA with Šidák’s post hoc test (**A**–**D**) and Mann-Whitney test (**G**).

**Figure 8:**
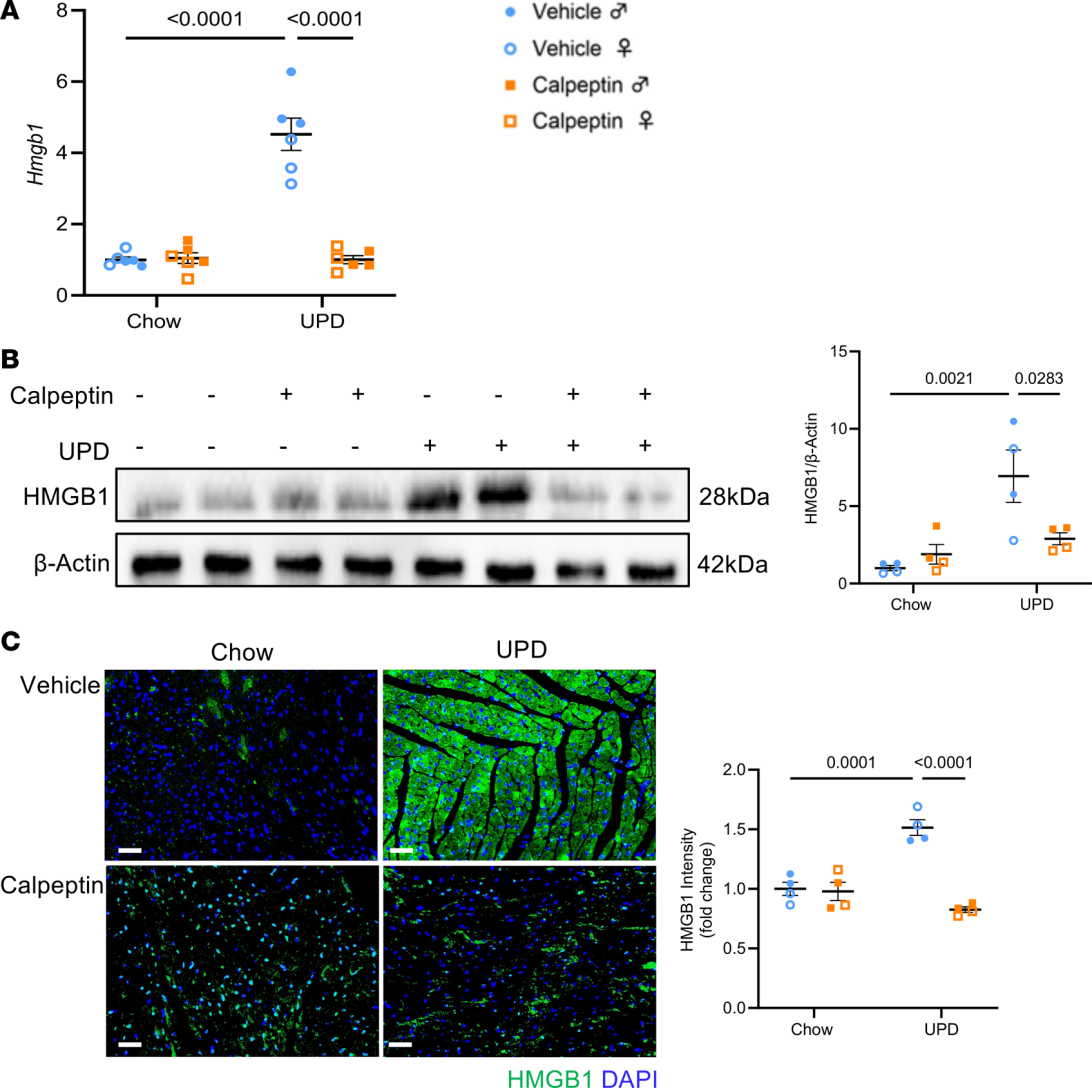
Pharmacological inhibition of calpain decreases cardiac HMGB1. (**A**) qPCR of *Hmgb1* (*n* = 6 hearts). (**B**) Representative immunoblots and quantification demonstrating HMGB1 expression in response to ultra-processed diet (UPD) and calpeptin treatment, where β-actin was used as a loading control (*n* = 4 hearts). (**C**) Representative images and quantification of cardiac HMGB1 staining (green) with DAPI-stained nuclei (blue) (scale bars: 50 μm) (*n* = 4 hearts). Data are presented as mean ± SEM, with solid and hollow symbols representing male and female mice, respectively. *P* values were calculated using 2-way ANOVA with Šidák’s post hoc test (**A**–**C**).

Immunofluorescent staining of the heart for Mac3 (Figure 7A) and CD86 (Figure 7B) indicated that calpeptin treatment countered UPD-induced macrophage infiltration in the myocardium, particularly impeding M1 macrophages from promoting inflammation. The augmented proinflammatory response as a result of the UPD was limited by treatment with calpeptin, as determined by diminished transcript levels of *Tnfa*, *Il1b*, *Il6*, and *Crp* in the myocardium (Figure 7C). In contrast, the transcripts of antiinflammatory genes (*Arg1*, *Il10*, and *Gdf15*) were enhanced in the hearts of mice treated with calpeptin receiving the UPD (Figure 7D). Similar to the genetic modulation, there was a decrease in the proinflammatory M1 (CD86^+^) macrophages in calpeptin-treated mice in comparison with the untreated UPD-fed group in both sexes (Supplemental Figure 12 and Figure 7, E–G). In line with the observations obtained from CAST-overexpressing hearts, HMGB1 levels were remarkably reduced in calpeptin-treated mice under UPD stress (Figure 8, A–C). Altogether, these data suggest that either genetic intervention or pharmacological treatment to block HMGB1 levels can counteract myocardial oxidative stress and the rising HMGB1 levels in the myocardium, thereby protecting the heart against UPD-induced inflammation and decelerating the onset and progression of HF.

### Calpain inhibition ameliorates trans FA–induced increased HMGB1 levels in cardiomyocytes and M1 macrophage polarization.

**Figure 9:**
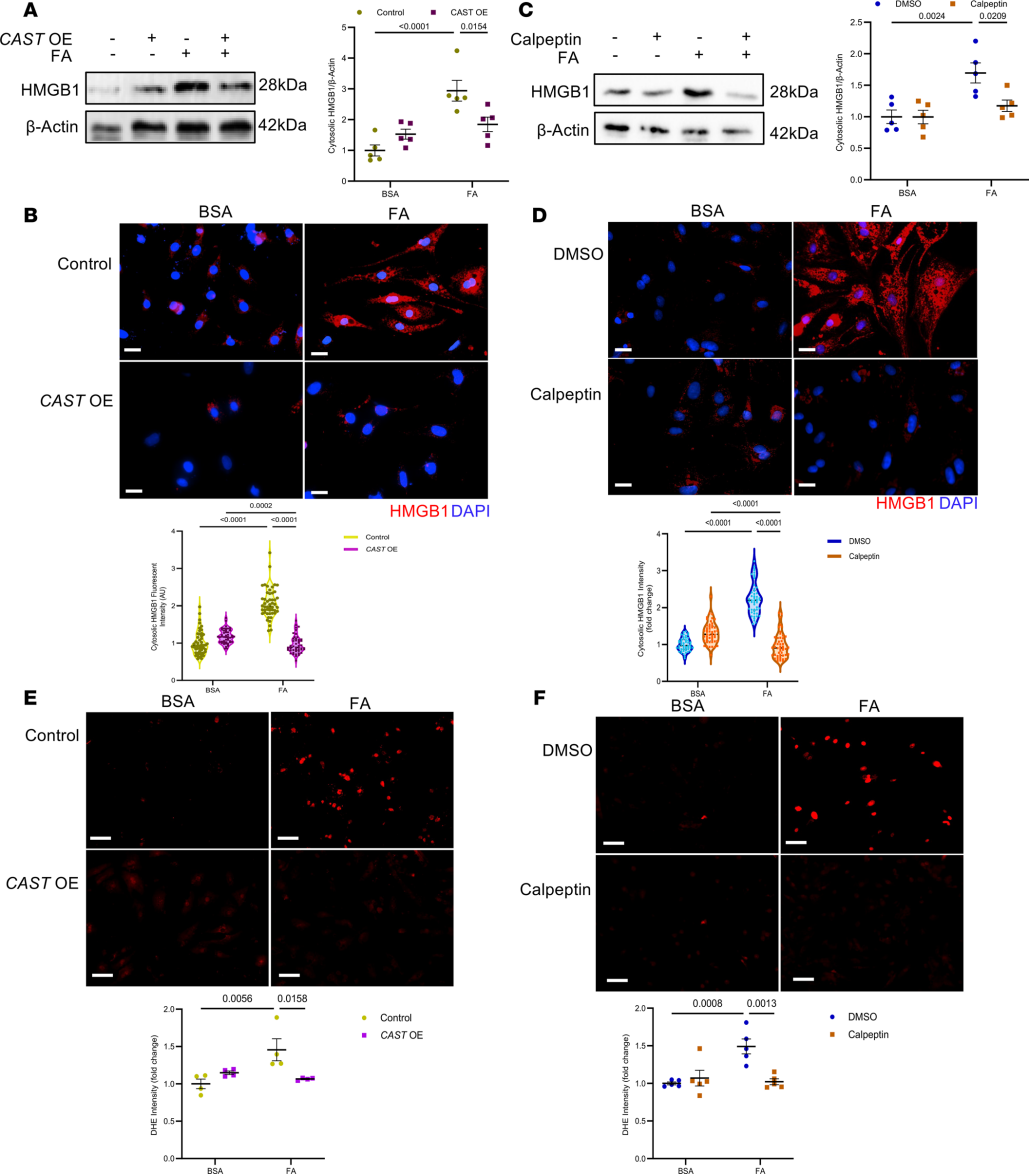
Inhibition of calpain alleviates oxidative stress and HMGB1 in neonatal rat cardiomyocytes (NRCMs) under stress of fatty acids (FAs). (**A**) Representative immunoblots and quantification of cytosolic HMGB1 expression in NRCMs with CAST overexpression in response to stimulation with FAs (palmitic acid [PA, 500 μM[, elaidic acid [EA, 500 μM], and linoelaidic acid [LA, 500 μM]) for 8 hours, where β-actin was used as a loading control (*n* = 5 experiments). (**B**) Representative images and quantification of cytosolic HMGB1 (red) with DAPI-stained nuclei (blue) (scale bars: 20 μm) (*n* = 50 cells in 3 experiments). (**C**) Representative immunoblots and quantification of cytosolic HMGB1 in NRCMs with calpeptin treatment (20 μM) under stimulation with FAs for 8 hours, where β-actin was used as a loading control (*n* = 5 experiments). (**D**) Representative images and quantification of cytosolic HMGB1 (red) with DAPI-stained nuclei (blue) (scale bars: 20 μm) (*n* = 50 cells in 3 experiments). (**E** and **F**) Representative images and quantification of DHE (scale bars: 50 μm) in NRCMs with CAST overexpression (**E**) (*n* = 4 experiments) or calpeptin treatment (**F**) (*n* = 5 experiments) under FAs for 8 hours. Data are presented as mean ± SEM. *P* values were calculated using 2-way ANOVA with Šidák’s post hoc test (**A**–**F**).

Furthermore, we obtained molecular evidence that the abundant FAs in UPFs trigger an inflammatory response through calpain in cardiomyocytes. NRCMs were stimulated with a combination of FAs, including PA (a saturated FA, 500 μM), LA (an omega-6 trans FA, 400 μM), and EA (a trans FA, 400 μM). In response to the stimulation of FAs for 8 hours, cytosolic HMGB1 in NRCMs was significantly increased compared with BSA controls, which was prevented by overexpression of *CAST* (Supplemental Figure 13 and Figure 9, A and B). Consistently, treatment with calpeptin also restrained FA-induced augmented HMGB1 distribution in the cytosol (Figure 9, C and D). Imbalanced oxidative status triggers HMGB1 cytosolic accumulation and likely its extracellular release (17). As a reactive form of oxygen, superoxide is a major component of oxidative stress. DHE staining supported the notion that FAs in UPFs gave rise to more ROS (Figure 9, E and F); however, either CAST overexpression (Figure 9E) or calpeptin treatment (Figure 9F) alleviated such an impact of FAs on the oxidative state in cardiomyocytes.

**Figure 10:**
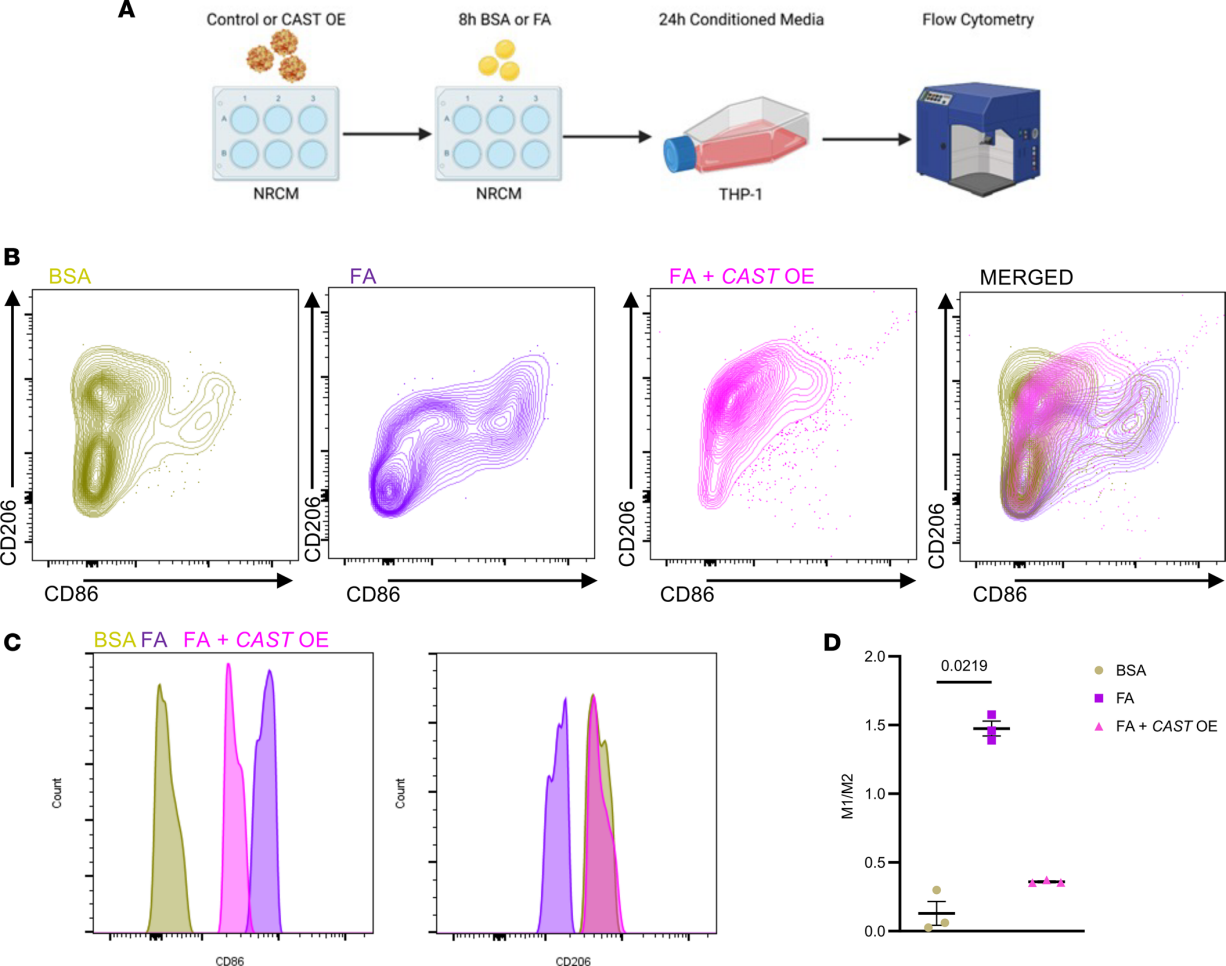
CAST overexpression (OE) in neonatal rat cardiomyocytes (NRCMs) reverses fatty acid–induced macrophage proinflammatory phenotype. (**A**) Experimental design of culture of THP-1 using the conditional media from NRCMs. (**B**) Representative flow contour plots of CD86^+^ and CD206^+^ macrophages following exposure to conditioned media. (**C**) Representative histograms displaying CD86^+^ and CD206^+^ macrophage subtypes. (**D**) Quantification of the relative ratio of M1 and M2 macrophages (M1/M2) (*n* = 3 experiments). Data are presented as mean ± SEM. *P* values were calculated using Kruskal-Wallis test with Dunn’s post hoc test (**D**). FA, fatty acid.

Enhanced polarization of M1 macrophages is considered a marker of a predominant proinflammatory response. HMGB1 released from stressed cardiomyocytes can induce M1 macrophage polarization and the subsequent activation of cardiac inflammatory response in a paracrine manner (14). Therefore, we evaluated the impact of FAs as well as the effects of inhibiting calpain in cardiomyocytes on subsequent macrophage polarization. Control and *CAST*-overexpressing NRCMs were stimulated with FAs (BSA as a control); the media were collected to culture phorbol 12-myristate 13-acetate–primed (PMA-primed) human monocytic leukemia cell line, THP-1 cells (Figure 10A). Flow cytometry analyses revealed that THP-1 cells treated with the conditioned media obtained from FA-stimulated NRCMs exhibited an increased proportion of M1 macrophages (CD86^+^ cells) compared with M2 macrophages (CD206^+^ cells) (Supplemental Figure 14 and Figure 10, B–D). Importantly, THP-1 cells exposed to the media obtained from NRCMs with CAST overexpression showed less M1 macrophage polarization (Supplemental Figure 14 and Figure 10, B–D). These results explain the in vivo phenotype, implying that increased cardiac HMGB1 triggers cardiac inflammation, while inhibition of calpain in cardiomyocytes can suppress this inflammatory response.

### CAST overexpression represses HMGB1 in hiPSC-CMs and the related inflammatory response.

**Figure 11:**
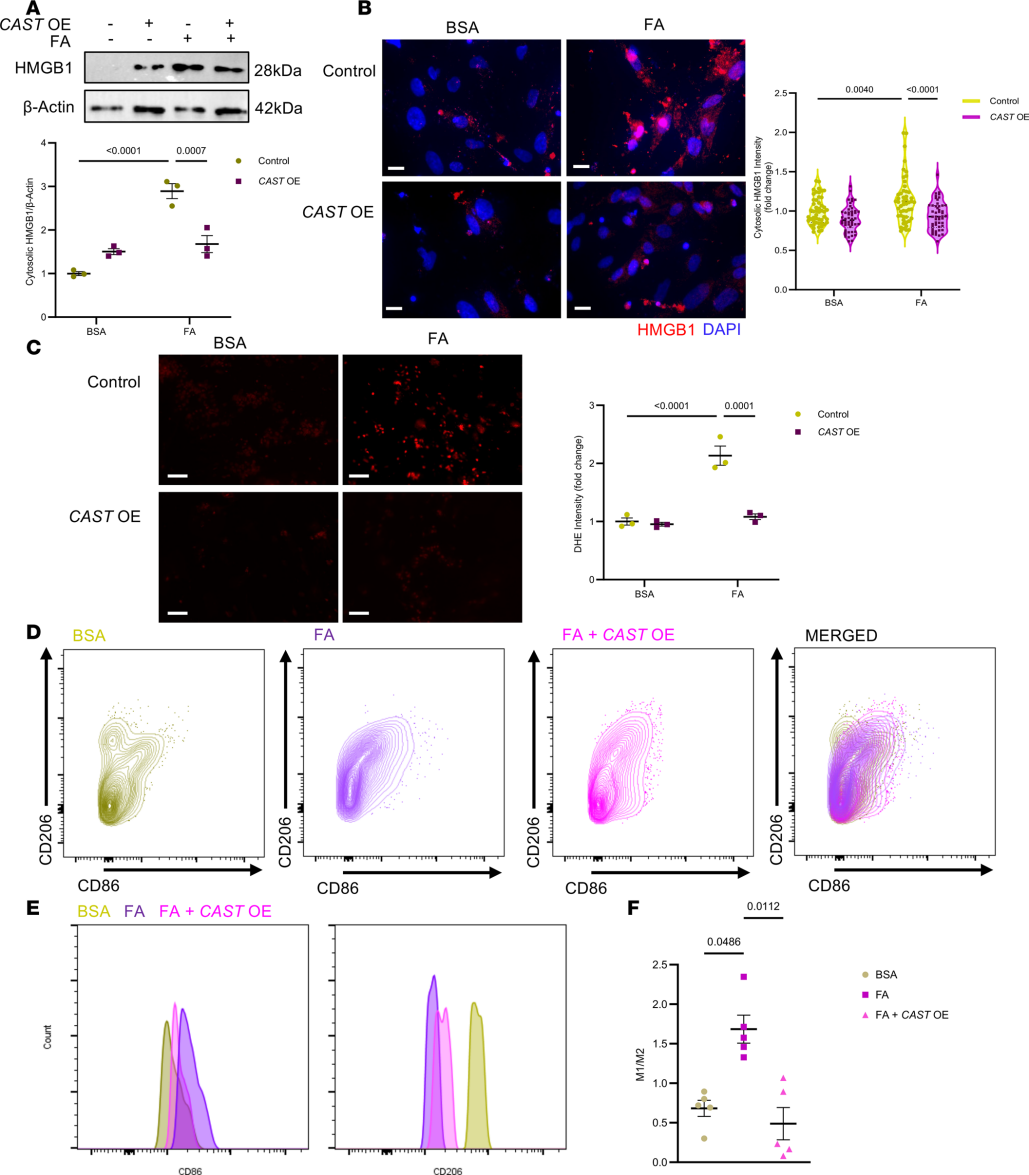
CAST overexpression in human induced pluripotent stem cell–derived cardiomyocytes (hiPSC-CMs) mitigates oxidative stress and subsequent inflammatory response. (**A**) Representative immunoblots and quantification of cytosolic HMGB1 expression in hiPSC-CMs with CAST overexpression under stimulation with fatty acids (FAs) (palmitic acid [PA, 500 μM], elaidic acid [EA, 500 μM], and linoelaidic acid [LA, 500 μM]) for 18 hours, where β-actin was used as a loading control (*n* = 3 experiments). (**B**) Representative images and quantification of cytosolic HMGB1 (red) with DAPI-stained nuclei (blue) (scale bars: 20 μm) (*n* = 50 cells in 3 experiments). (**C**) Representative images and quantification of DHE (scale bars: 50 μm) in hiPSC-CMs with CAST overexpression under FA stimulation for 18 hours (*n* = 3 experiments). (**D**–**F**) Representative flow contour plots (**D**), histograms of CD86^+^ and CD206^+^ macrophages (**E**), and the ratio of M1 and M2 macrophages (**F**) following exposure to the conditioned media collected from hiPSC-CMs. Data are presented as mean ± SEM. *P* values were calculated using 2-way ANOVA with Šidák’s post hoc test (**A**–**C**) or Kruskal-Wallis with Dunn’s post hoc test (**F**).

We next evaluated the above mechanisms underlying trans FAs in UPD mediating inflammation using hiPSC-CMs. The cells were transfected with either *CAST* or *Flag* cDNA, as a control, followed by stimulation with FAs (500 μM PA, 400 μM LA, 400 μM EA) for 18 hours. In line with the observations detected in NRCMs, cytosolic HMGB1 was upregulated by FAs in control cells (Figure 11, A and B), along with the higher content of ROS (Figure 11C). CAST overexpression suppressed FA-increased HMGB1 levels and oxidative stress (Figure 11, A–C). Moreover, PMA-primed THP-1 cells were exposed to the conditioned media collected from the cultured hiPSC-CMs. After 24 hours of incubation, polarization of THP-1 cells was assessed by flow cytometry (Supplemental Figure 15). The higher proportion of M1 macrophages was detected following the incubation in media from hiPSC-CMs treated with FAs, which was reversed when THP-1 cells were exposed to the media from hiPSC-CMs with CAST overexpression (Figure 11, D–F). Overall, human-relevant results have also endorsed the findings that saturated and trans FAs in the UPD instigate HMGB1-associated activation of proinflammatory macrophages.

### Prevention of oxidative stress has the potential to reduce HMGB1 and reverse cardiac dysfunction.

**Figure 12:**
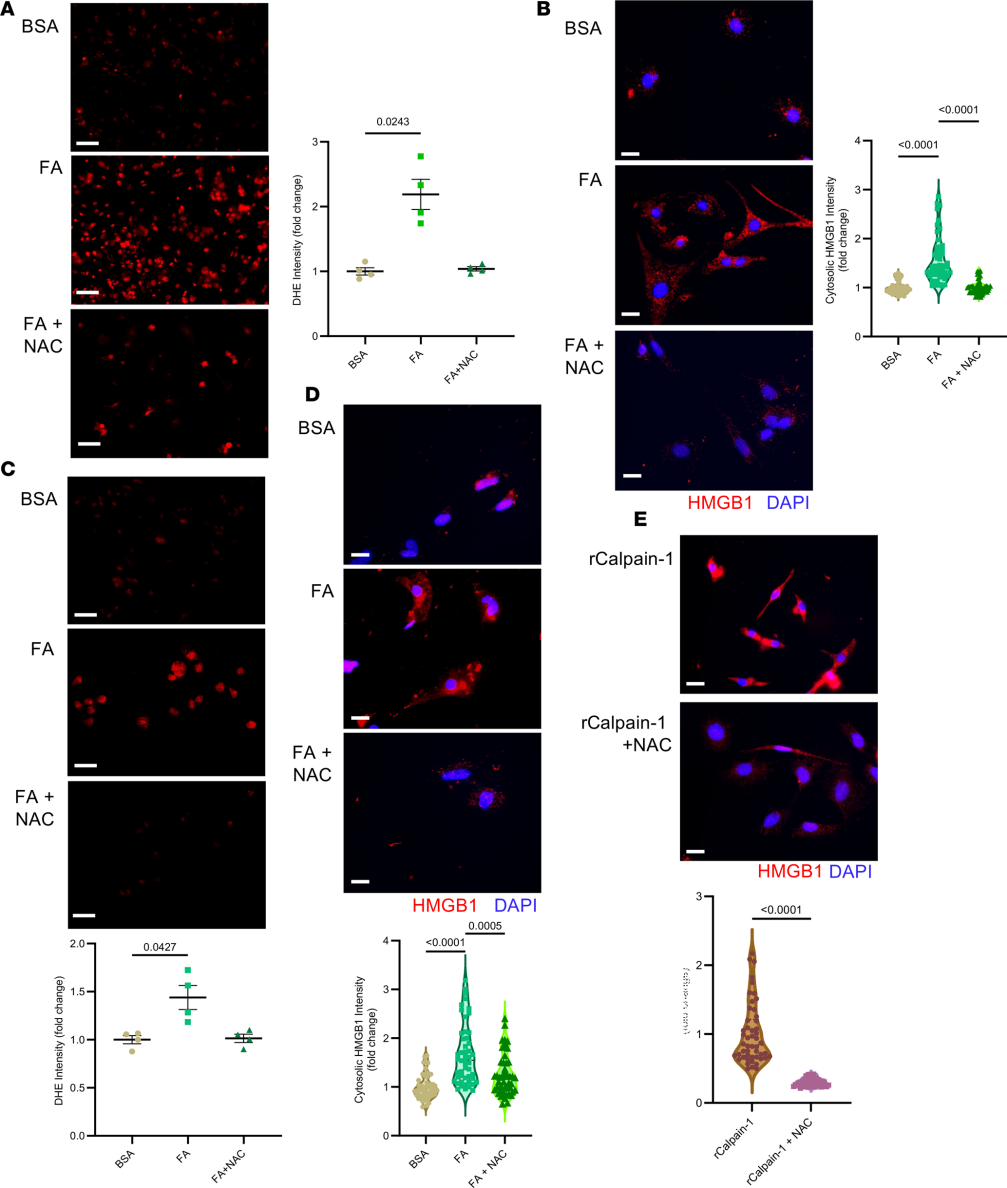
Inhibiting reactive oxygen species (ROS) reduces cytosolic HMGB1 in cardiomyocytes. (**A** and **B**) Representative images and quantification of DHE (scale bars: 50 μm) (*n* = 4 experiments) (**A**) and cytosolic HMGB1 staining (red) with DAPI-stained nuclei (blue) (scale bars: 20 μm) (*n* = 50 cells across 3 experiments) (**B**) in NRCMs stimulated for 8 hours with FAs with or without NAC treatment (4 mM). (**C** and **D**) Representative images and quantification of DHE (scale bars: 50 μm) (*n* = 4 experiments) (**C**) and cytosolic HMGB1 staining (red) with DAPI-stained nuclei (blue) (scale bars: 20 μm) (*n* = 50 cells across 3 experiments) (**D**) in human induced pluripotent stem cell–derived cardiomyocytes (hiPSC-CMs) stimulated for 18 hours with FAs with or without NAC (4 mM). (**E**) Representative images and quantification of cytosolic HMGB1 staining (red) with DAPI-stained nuclei (blue) (scale bars: 20 μm) (*n* = 50 cells across 3 experiments) in NRCMs stimulated with recombinant calpain-1 (rCalpain-1) in the presence or absence of NAC (4 mM) for 8 hours. Data are presented as mean ± SEM. *P* values were calculated using Kruskal-Wallis with Dunn’s post hoc test (**A**–**D**) and Mann-Whitney test (**E**).

We further provided a causative link between ROS and HMGB1 accumulation in the cytosol in NRCMs and hiPSC-CMs. First, NRCMs were stimulated with hydrogen peroxide (H_2_O_2_, 200 μM) for 24 hours, detecting increased intracellular ROS (Supplemental Figure 16A). Increased cytosolic HMGB1 was also detected in response to stimulation with this ROS-inducing compound (Supplemental Figure 16B), supporting the idea that oxidative stress promotes HMGB1 cytosolic distribution. Importantly, treatment with NAC (4 mM), a potent ROS inhibitor, successfully diminished DHE intensity in NRCMs under FA stress (Figure 12A), accompanied by a decline in HMGB1 in the cytosol (Figure 12B).

Consistent results were noted in hiPSC-CMs, whereby 24 hours of H_2_O_2_ treatment (200 μM) significantly increased the levels of ROS (Supplemental Figure 16C) and cytosolic HMGB1 (Supplemental Figure 16D). The detrimental outcomes induced by FA stress were rescued by simultaneous administration of NAC (4 mM) (Figure 12, C and D). Additionally, recombinant calpain-1–increased cytosolic HMGB1 was also ameliorated by concurrent treatment of NAC in vitro (Figure 12E). These data illustrate that calpain-increased cytosolic HMGB1 is through an ROS-dependent manner in cardiomyocytes under stress of saturated and trans FAs.

**Figure 13:**
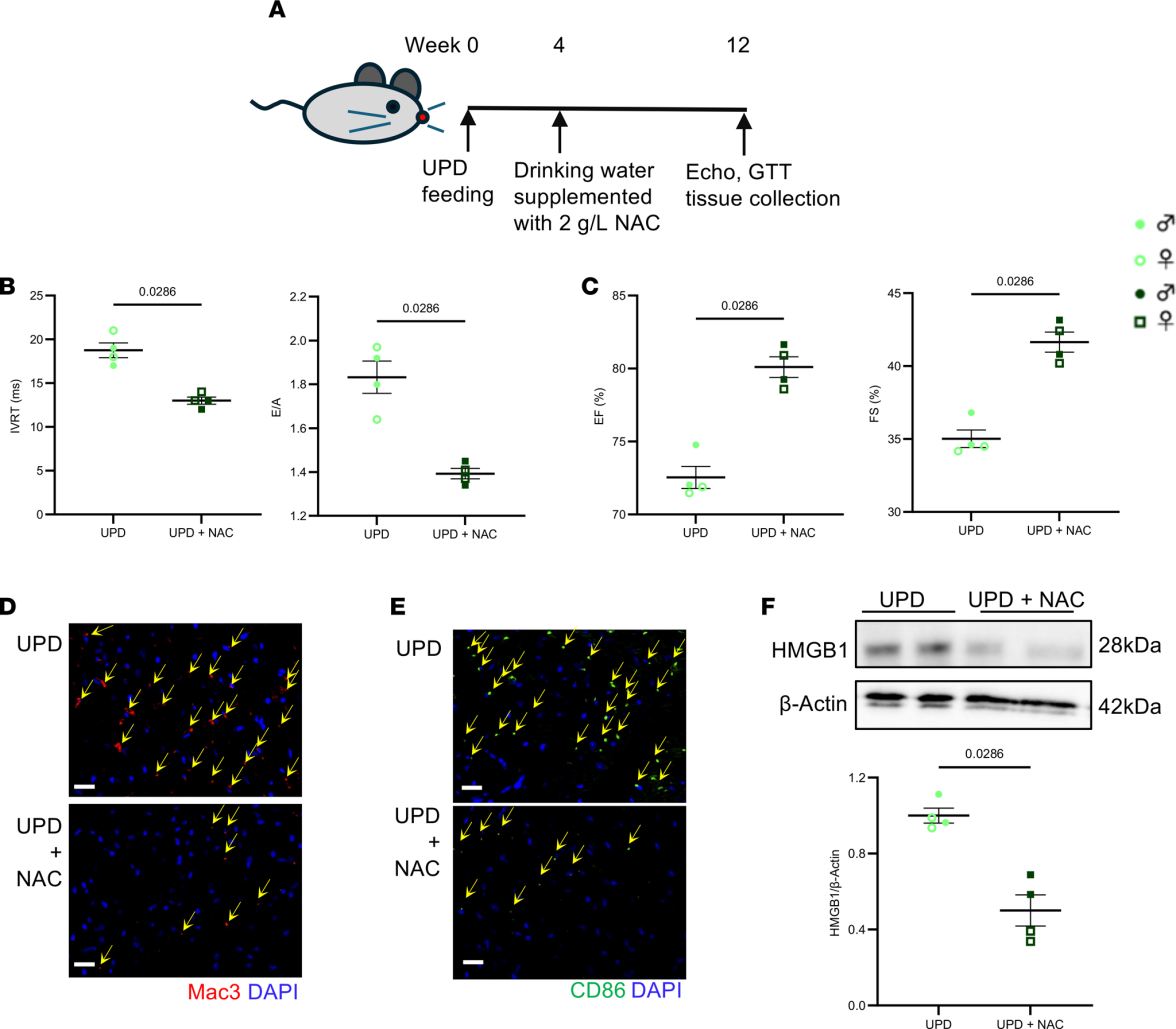
Pharmacological inhibitor of reactive oxygen species (ROS) reduces cardiac dysfunction. (**A**) Schematic of the experimental design. (**B**) Isovolumic relaxation time (IVRT) and ratio of peak velocity blood flow from left ventricular relaxation in early diastole in late diastole (E/A) (*n* = 4 mice). (**C**) Percentage of ejection fraction (EF%) and fractional shortening (FS%) (*n* = 4 mice). (**D**) Representative images of cardiac Mac3 staining (red, arrows) with DAPI-stained nuclei (blue) (scale bars: 20 μm) (4 hearts). (**E**) Representative images of cardiac CD86 staining (green, arrows) with DAPI-stained nuclei (blue) (scale bars: 20 μm) (4 hearts). (**F**) Representative immunoblots and quantification demonstrating HMGB1 expression in response to UPD and NAC treatment, where β-actin was used as a loading control (*n* = 4 hearts). Data are presented as mean ± SEM. *P* values were calculated using Mann-Whitney tests (**B**, **C**, and **F**).

Furthermore, we sought the treatment potential of NAC in vivo through the blockage of HMGB1. Mice fed with UPD for 4 weeks were administered NAC in drinking water for 8 weeks (Figure 13A). Although systemic metabolic profiles were not altered by the treatment (Supplemental Figure 17), UPD-damaged cardiac function was reversed (Figure 13, B and C, and Supplemental Table 5). Importantly, myocardial inflammation was reduced by NAC treatment, as indicated by fewer Mac3^+^ cells (Figure 13D) and CD86^+^ cells (Figure 13E) in the heart. Reduction of HMGB1 was obtained by NAC treatment (Figure 13F). Altogether, the findings provide evidence that blockage of ROS has the beneficial effects of inhibiting HMGB1 and subsequently reversing cardiac dysfunction under stress.

## Discussion

Diet-induced metabolic stress is a high-risk factor for cardiac dysfunction; however, inadequate therapies preventing or treating HF in such a context are due to a limited understanding of the underlying mechanisms. This study introduces what we believe is a new model mimicking the diseased condition induced by a UPD, in both sexes, wherein HF occurs at least partially due to myocardial inflammation. Calpain-1 in the heart is upregulated in response to metabolic stress, acting as a stimulator for promoting ROS. Intracellular oxidative stress boosts HMGB1 in cardiomyocytes, thereby aggravating the inflammatory response in the heart. Importantly, it has been shown by functional studies that either genetic or pharmacological inhibition of calpain-1 reduced ROS and triggered antiinflammatory processes in the heart. Our findings demonstrate that the induction of myocardial inflammation through the signaling cascade of calpain-1, ROS, and HMGB1 in cardiomyocytes acts as a potential therapeutic avenue for preventing or treating UPF-associated HF.

### Health risks associated with high UPF consumption.

A significant portion of the daily calorie intake comes from UPFs. This is the main cause of the increasing prevalence of obesity, type 2 diabetes, and metabolic syndrome. Higher UPF consumption is also associated with an increased risk of cardiovascular diseases. A higher content of saturated FAs and trans FAs is recognized as the harmful aspect causing increased morbidity and mortality, likely through lipid abnormalities and inflammation; however, the exact mechanisms are still under investigation. The UPD used in our study consists of hydrogenated coconut oil, peanut butter, and chocolate spread, imitating trans FA–containing UPFs consumed by humans. The mice fed such a diet were more vulnerable to impaired systolic and diastolic function compared with the other high-calorie diets, implying that a UPD contains more harmful elements to cardiac health. The acids LA and EA are *trans* isomers of linoleic and oleic acid, respectively, and are both found in hydrogenated vegetable oils and some dairy products that are routinely consumed and linked to health risks. Therefore, the preclinical model established in our study can also be used to investigate other UPF-linked common health concerns.

### Sex differences in HF.

HF is classified into 2 main subcategories dependent upon the diastolic function. HF with preserved ejection fraction (HFpEF), where EF is greater than 50%, accounts for more than 50% of HF in clinics, and develops due to aging, metabolic syndrome, or inflammatory stress. Thirty-five percent of people with HF have HF with reduced EF (HFrEF), where EF is below 40%, and is commonly a result of ischemia or other underlying cardiovascular disorders (12). HF phenotypes, however, differ between sexes. Although women with HF have a longer survival rate and a lower risk of sudden death, HFpEF has a greater prevalence in women (28). Of note, metabolic stress may contribute to HF to a larger extent in women; however, we found that the UPD-induced diabetic condition and the development of the associated HF were similar in both male and female mice. On the other hand, sex is also a biological variable that affects systemic immune responses, but comparable UPD-associated cardiac inflammation has been demonstrated in this study. All these findings indicate that the consumption of UPFs exert equal detrimental effects on cardiac function in both sexes. However, more basic and clinical research are warranted to establish the correlation of UPFs and cardiac performance between men and women.

### Calpain-1 induces oxidative stress in the heart.

The calpain family is involved in various pathophysiological processes in the heart. For instance, calpain activation contributes to adverse cardiac remodeling, cell death, impaired contractility, and subsequent HF under pathological stresses (18). In particular, cardiac overexpression of calpain-1 causes HF spontaneously (22). Transgenic overexpression of mitochondria-targeted calpain-1 also exacerbated mitochondrial oxidative stress and cell death in isolated hearts following either ischemia/reperfusion injury or streptozotocin injection used to mimic type 1 diabetes (24). Importantly, oxidative stress and inflammation in the heart of diabetic rats were likely mediated via calpain-1 (29). These findings are supportive of the current study, which shows that increased calpain-1 in the heart was sufficient to trigger detrimental effects on cardiac function in response to UPD-induced metabolic stress.

Calpain-1 is also associated with the redox status in cells. Calpain-1 activation in the myocardium is mediated through the oxidase-dependent pathways under pathological stresses (19, 21). In turn, activated calpain-1 augmented ROS production, particularly from the mitochondria, as calpain-1 increased leakage of electrons from the mitochondrial respiratory chain, provoking the production of ROS, such as superoxide (30). Our study showed consistent observations, whereby the higher levels of calpain-1 were accompanied by more ROS in both in vivo and in vitro models. Importantly, either the recombinant calpain-1 protein or overexpression of calpain-1 increased DHE^+^ cells in the absence of other kinds of stresses, indicative of more superoxide in cardiomyocytes. However, the direct mechanisms underlying these effects require further investigation.

### Oxidation of HMGB1 is associated with inflammation.

Oxidative stress, specifically through ROS, can contribute to the increase in inflammatory responses. For example, ROS activates intracellular signaling pathways and the downstream transcription factors, such as nuclear factor κB (NF-κB), that upregulate cytokine production in immune cells (31). In addition, oxidation influences cytokine function by modulating the cytokine configuration and activity when oxidized, affecting its binding to specific receptors (32).

As an inflammatory mediator in the extracellular space, we have demonstrated that cardiomyocyte-sourced HMGB1 can amplify the inflammatory response in the heart (14). Previous studies have demonstrated that oxidative conditions elicit a structural change in HMGB1 via the formation of a disulfide bond in its functional domain. Oxidized HMGB1 facilitates its translocation from the nucleus to the cytoplasm, where HMGB1 is prone to be released from the cells through different mechanisms (16, 33). Convincingly, ROS (H_2_O_2_) triggers activation and release of HMGB1 from macrophages and monocytes (15). Here, we first detected profound ROS along with increased cytosolic HMGB1 in failing hearts upon metabolic stress. H_2_O_2_ directly upregulated the levels of HMGB1 in the cytosol, while ROS inhibition prevented such an increase in cardiomyocytes. Most importantly, NAC treatment of UPD-fed mice inhibited ROS reduced HMGB1 and the related cardiac dysfunction. Our study provides molecular and functional evidence that oxidative stress causes HMGB1 activation and myocardial inflammation, although whether ROS mediates the transcription and/or translation of HMGB1 in the myocardium needs to be explored further.

### Potential treatment strategy to inhibit ROS and inflammation by targeting calpain-1.

Targeted gene inactivation of calpain-1 by interrupting exon 4 in mice suppresses oxidative stress (34), which is highly supportive of our observations in the heart. To seek the treatment feasibility of HF by blocking calpain-1 during the progression of pathological conditions, we aimed to evaluate the effects of inhibiting endogenously excessive calpain-1 in the established HF model. Since CAST is an endogenous inhibitor of the calpain family, it shows treatment potential in various disease conditions by limiting the harmful action of overactive calpain. In the heart, its overexpression reduces cardiac hypertrophy induced by angiotensin-II (27). In addition, the protective effects of CAST in the heart by preventing mitochondrial calpain in response to ischemia and reperfusion have been reported (24). This study, for the first time to our knowledge, reveals the benefits of CAST in inhibiting oxidative stress and inflammation in the myocardium, as evidenced by the changes in the genes participating in anti–oxidative stress and antiinflammatory responses. Noticeably, compared with HFD (14), the UPD enhanced the susceptibility of the heart to inflammatory activation and cardiac dysfunction in a shorter time frame. With the changes in our diet, the rise in UPFs may result in an increased incidence of HF. Therefore, CAST and its synthetic analogs likely hold promise as therapeutic agents to prevent or treat HF induced by UPFs.

Of note, the treatments were provided after cardiac function was impaired, 4 weeks after UPD feeding. The findings indicate treatment potential to reverse HF in diabetes, although the feasibility of prevention of HF requires further investigation. In addition, either genetic (overexpression of CAST) or pharmacological (calpeptin) inhibition does not block calpain-1 specifically. Of note, we found that calpain-1 was increased in the failing diabetic hearts and that recombinant calpain-1 augmented HMGB1 expression. Although calpain-2 (also highly expressed in the heart) was not affected in the myocardium upon metabolic stress in humans and mice, the function of calpain-2 in the diabetic heart and the treatment effects of CAST or calpeptin by various doses is yet to be explored.

### Conclusion.

The present research illustrates that UPF-increased calpain-1 interferes with the redox state in the myocardium, leading to HMGB1 activation and cardiac inflammation. We have provided proof-of-concept evidence that calpain inhibition mitigates oxidative stress–associated myocardial inflammation triggered by UPFs in both sexes.

## Methods

### Sex as a biological variable.

All human and animal studies involved the assessment of both male and female participants or mice, with similar findings being reported for both sexes.

### Animal studies.

All animals were purchased from Envigo.

### Induction of metabolic stress using a UPD.

Male and female C57BL/6J mice aged approximately 7–8 weeks old were provided with either a standard chow diet or UPD (LBS Biotechnology, U8954A01R 00344) ad libitum for 12 weeks.

### AAV9 gene delivery.

Cardiac-specific CAST overexpression was achieved by tail vein injection of AAV9-cTnT-*CAST* at a dosage of 1 × 10^11^ viral particles. The mice injected with AAV9-cTnT-*eGfp* served as control mice.

### Drug administration.

The pharmacological calpain inhibitor calpeptin (MedChemExpress, HY-100223), was administered to mice intravenously at a dose of 10 mg/kg weekly for 8 weeks (35). In addition, NAC (Sigma-Aldrich, A9165) was provided in the drinking water ad libitum at 2 g/L for 8 weeks (36).

### Echocardiography.

Transthoracic 2-dimensional M-mode and pulse wave Doppler ultrasound images were obtained using the Acuson Sequoia C256 system. Left ventricular chamber and wall dimensions, wall thickness, and diastolic function parameters (IVRT and the E/A ratio) were measured, and systolic function parameters (FS% and EF%) were calculated accordingly.

### Human samples.

To perform immunofluorescent and DHE staining on cryosections of human hearts, heart tissues were obtained from BioIVT (formerly Asterand) from either consented donors without cardiovascular diseases as controls (without metabolic and cardiovascular diseases) or donors diagnosed with metabolic syndrome–associated ischemia, atrial fibrillation, or HF.

To perform RNA sequencing, quantitative PCR (qPCR), and immunoblotting analyses, more human hearts were obtained to include hearts from normal healthy donors (without metabolic syndrome and cardiovascular diseases) and HF donors (metabolic syndrome and HF).

### Statistics.

Data are presented as bar/dot plots showing mean ± SEM. Where sample sizes were 5 or greater, the Shapiro-Wilk test was conducted to first determine whether data was normally distributed. Normally distributed data sets were analyzed using ordinary 2-way ANOVA followed by appropriate post hoc tests, whereas comparisons between 2 groups were performed using a 2-tailed Student’s *t* test. The non-parametric equivalents were utilized for skewed data and data sets where sample sizes were below 5. Mann-Whitney tests were used for non-parametric 2-group comparisons. Statistical analyses were performed using GraphPad Prism 10 software and indicated in legends. A *P* value of less than 0.05 was considered statistically significant.

### Study approval.

All animal studies were conducted in agreement with the United Kingdom Animals (Scientific Procedures) Act 1986 following ARRIVE guidelines and were approved by the University of Manchester Ethics Committee. For studies regarding human heart tissue, ethical approval and consent was obtained following the United Kingdom Human Tissue Authority regulations or from the United Network for Organ Sharing (UNOS) through IIAM and Novabiosis, with next-of-kin informed consent.

### Data availability.

The data, experimental materials, and methods are available from the corresponding author on reasonable request for the purpose of reproducing the results or replicating the procedure. Detailed methods are available in the supplemental material. Values for all data points in graphs are reported in the Supporting Data Values file.

## Author contributions

The project conceptualization as detailed in this manuscript was done by WL. CR contributed significantly to the experimental design, animal work, functional and molecular assessments, data acquisition, analysis, and interpretation. SRG, NHOA, XC, OF, and YH helped with animal work and data collection. NHOA, JZ, YL, XZ, and TW helped with hiPSC culture. ARV performed data collection regarding murine RNA sequencing. RREA and TMAM provided human heart tissues. GJH and TW provided mentorship and alongside HX reviewed the manuscript. BDK also provided mentorship for the work. EJC reviewed in vivo studies. WL drafted the manuscript with contributions from CR, SRG, and XC.

## Conflict of interest

TMAM holds equities in Tenaya Therapeutics.

## Funding support

This work is the result of NIH funding, in part, and is subject to the NIH Public Access Policy. Through acceptance of this federal funding, the NIH has been given a right to make the work publicly available in PubMed Central.

- British Heart Foundation grants FS/PhD/22/29307, PG/22/10904, and PG/22/11075 (to WL).
- British Heart Foundation Accelerator award AA/18/4/34221 (to the University of Manchester).
- British Heart Foundation Personal Chair (to BDK).
- NIH grants R01HL147921 and P30GM127607 (to TMAM).
- NIH grant F32HL149140 (to RREA).
- Department of Defense grant W81XWH-20-1-0419 (to TMAM).
- American Heart Association grant 16SDG29950012 (to TMAM).

## Supplementary Material

Supplemental data

Unedited blot and gel images

Supporting data values

## Version 1 04/22/2026

In-Press Preview

## Version 2 06/08/2026

Electronic publication
