# Supplementary material for: Inhibition of calpain-mediated HMGB1 alleviates cardiac inflammation and dysfunction induced by ultra-processed foods
Source: JCI Insight. 2026 Apr 9;11(11):e199622. doi: 10.1172/jci.insight.199622 (PMC13313562; doi:10.1172/jci.insight.199622)
Supplement: Supplemental data [file jciinsight-11-199622-s132.pdf]

SUPPLEMENTAL MATERIAL

Supplemental Methods

Animal studies

All animal studies were conducted in agreement with the United Kingdom Animals (Scientific Procedures) Act 1986 following ARRIVE guidelines and were approved by the University of Manchester Ethics Committee. All the animals were housed in a clean, pathogen-free facility at the University of Manchester. Both male and female C57BL/6J mice were purchased from Envigo (UK), as well as pregnant Sprague-Dawley rats. With the exception of strain, age and sex, no exclusion/inclusion criteria were set, and all animals are presented in the data. Animals were randomly allocated into groups using an online random group generator and investigators were blinded to experimental groups for data analysis.

Tohoku Hospital Pediatrics-1 cells (THP-1) cell line

Human leukemia monocytic cell line, THP-1 cells, were purchased from the American Type Culture Collection (ATCC, TIB-202). Cells were maintained in RPMI Medium 1640 (Gibco, A10491) with 0.05 mM β-mercaptoethanol and fetal bovine serum (10% v/v).

Human induced pluripotent stem cells-derived cardiomyocytes (hiPSC-CMs)

The hiPSC 02C9 cells were established from adult human dermal fibroblasts (Invitrogen) using the CytoTune-iPS 2.0 Sendai Reprogramming Kit (Life Technologies; A16518) according to manufacturer's instructions. The hiPSCs were maintained and differentiated into cardiomyocytes as described previously (1). In short, hiPSCs were cultured in mTeSR Plus media (Stem Cell Technologies, 100-0276) on Geltrex-coated plates (Life Technologies, A1413302) and differentiated into cardiomyocytes using 4 µM CHIR99021 and 5 µM IWP2 (Major Resources Table) for 48 hours each, in RPMI1640 HEPES Glutamax medium (Life Technologies, 72400021) with B27 minus insulin supplement.

Neonatal rat cardiomyocytes (NRCMs)

Neonatal rat cardiomyocytes were isolated from 2/3-day-old Sprague-Dawley rats as described previously (2). Briefly, hearts were excised into small pieces and subjected to multiple cycles of enzymatic digestion, for 6 minutes per cycle, in 7ml of buffer [116 mM NaCl, 20 mM, 1 mM NaH_2PO_4, 6 mM glucose, 5 mM KCl, 0.8 mM $MgSO_4$, pH 7.4] containing 0.33 U/mL Collagenase A (Roche, 10103586001) and 100 mg/mL pancreatin (Sigma-Aldrich, P3292). NRCMs were maintained in the medium containing 80% DMEM, 20% M199, 1% FBS, penicillin-streptomycin, fungizone, and 1 μM bromodeoxyuridine.

Human samples

To perform immunofluorescent and Dihydroethidium (DHE) staining, human heart tissues were purchased from Asterand (BioIVT, UK). The samples were collected from consented donors without cardiovascular diseases as controls (female and male, BMI<25) and donors diagnosed with metabolic syndrome-associated ischemia, atrial fibrillation, coronary artery disease, or congestive heart failure (female and male, BMI 30-35). Asterand obtained ethical approval and consent following the United Kingdom Human Tissue Authority regulations. The tissue was embedded in OCT embedding matrix and stored in -80°C for histological analyses. Cryosections were cut at a thickness of 10μm using the Leica CM3050S Cryostat microtome.

On the other hand, to perform RNA Sequencing, qPCR and immunoblots, human hearts were obtained from the United Network for Organ Sharing (UNOS) through IIAM and Novabiosis, with next of kin informed consent. Human hearts were classified according to the following inclusion and exclusion criteria. Normal healthy donors: inclusion criteria: 1) aged 50 or above and, 2) ejection fraction>55%; exclusion criteria: 1) history of myocardial infarction, 2) pregnant women, 3) decompensated heart failure, 4) severe kidney disease, 5) asthma or severe chronic lung disease, 6) cardiac pacemaker or implantable defibrillator, 7) cerebral aneurysm clip, 8) neural stimulator, and 9) history of diabetes. HF donors: inclusion criteria: 1) aged 50 or above, and 2) heart failure with history of at least one of chronic kidney disease,

hypertension or diabetes; exclusion criteria: 1) pregnant women, 2) asthma or severe chronic lung disease, 3) cerebral aneurysm clip, and 4) neural stimulator.

All experimental procedures conducted on human samples were approved by the University of Manchester Research Ethics Committees and in agreement with the Declaration of Helsinki.

Induction of metabolic stress

Male and female C57BL/6J mice aged ~7-8 weeks old were provided with either a standard chow diet (Envigo, 2018 Teklad Global 13 kcal% fat, 18 kcal% protein Rodent diet) or ultra-processed diet (UPD) (15%, 22%, 30% and 14% calories from protein, fat, sugars and starch respectively, LBS Biotechnology U8954A01R 00344) *ad libitum* for 12 weeks. Alternatively, mice were induced with metabolic disorders by various routes, including fed with high-fat diet (HFD) (60% calorie from fat, SDS, 824054) for 25 weeks; fed with HFD (45% calorie from fat, SDS, 824018); fed with HFD plus intraperitoneal injection streptozotocin (STZ, 40 mg/kg daily for 3 days); or HFD (60% calorie from fat) with regular drinking water containing Nω-nitro-l-arginine methyl ester (L-NAME) (Sigma-Aldrich, N5751) at a concentration of 0.5 g/L (pH 7.4) (3).

UPD Ingredient	Component ID	Quantity g/kg
Maltodextrin	60343A99	154
Sucrose	61254A99	224
Crude cellulose	64254A99	50
Casein	63400A99	119
L-cystine	65799A99	3
Soybean Oil	61900A99	35
Hydrogenated Coconut Oil	61958A99	50
Cholesterol	61968A99	12.5

Sodium Chloride	65400A99	5
Vitamin pre-mix PV AIN93M_G1%	90002A99	10
Choline bitartrate	65890A99	2.5
Mineral pre-mix PM AIN93M_G3.5%	91001A99	35
Chocolate spread	67788A99	150
Peanut butter	67789A99	150

Adeno-associated virus 9 (AAV9) gene delivery

Cardiac-specific CAST overexpression was achieved by using an AAV9 vector carrying human *CAST* cDNA sequence (VectorBuilder, VB221202-1131rtv) under the control of cardiac troponin T (cTnT) promoter. Following the first 4 weeks of chow or UPD feeding, CAST overexpression was achieved by tail vein injection of AAV9-cTnT-*CAST* at a dosage of $1x10^{11}$ viral particles per mouse. The mice injected with AAV9-cTnT-e*Gfp* served as control mice.

Drug administration

Following the first 4 weeks of chow or UPD feeding, the pharmacological calpain inhibitor calpeptin (MedChemExpress, HY-100223), was administered to C57BL/6 mice intravenously at a dose of 10 mg/kg weekly for 8 weeks (4). A 3 mg/mL calpeptin stock solution was freshly prepared weekly using Tween-80 (15% v/v) and 0.9% saline (85% v/v).

Following 4 weeks of UPD feeding, mice were administered with the ROS scavenger N-acetyl-cysteine (NAC) dissolved in their drinking water for 8 weeks at a concentration of 2 g/L (5). Water containing NAC was refreshed every 2-3 days to ensure maximal efficacy of the drug.

Metabolic profile measurements

Fasting blood glucose levels in the mice were measured after 16 hours of fasting using an

Accu-Chek Aviva glucometer from blood collected from the lateral tail vein. For the glucose tolerance test (GTT), after 16 hours of fasting, an intraperitoneal injection of 2g/kg glucose was administered. Blood was collected from the lateral tail vein, and glucose levels were measured at 30-minute intervals over 2 hours using an Accu-Chek Aviva glucometer.

Two-dimensional echocardiography

Mice were anesthetized using 2% isoflurane mixed with 100% oxygen at a rate of 1.5 L/min. Transthoracic two-dimensional M-mode and pulse wave Doppler ultrasound images were obtained using the Acuson Sequoia C256 system. Left ventricular chamber dimension and wall thickness, diastolic function parameters [isovolumic relaxation time (IVRT) and the ratio of early (E) to late (A) diastolic filling velocities of the left ventricle (E/A)] were measured; and systolic function parameters [fractional shortening (FS%) and ejection fraction (EF%)] were calculated accordingly.

Tissue processing

After cervical dislocation, the heart was dissected and cut cross-sectionally into two halves. The lower apical part was snap-frozen and stored for molecular assessments, while the upper basal part was fixed in 4% paraformaldehyde (PFA) for histological assessments. Tissue processing was conducted by consecutive dehydration with 75%, 90%, and 99% ethanol followed by xylene clearance overnight. The tissues were immersed in paraffin wax and embedded to produce tissue blocks for microtomy. The embedded samples were sectioned at 5 μM using a Leica RM2135 paraffin microtome.

Hematoxylin and Eosin (H&E) staining

The paraffin sections were deparaffinized in xylene overnight, followed by sequential rehydration in descending order of IMS concentrations (100%, 75%, and 50%) for 10 minutes each. Sections were then stained in Harris' hematoxylin (Sigma-Aldrich, HHS32) for 5 minutes. Thereafter, the sections were differentiated in acid alcohol [1% (v/v) hydrochloric acid, 70%

(v/v) ethanol] for 10 seconds to get rid of the excess background and then counterstained with Eosin (Thermo Scientific, 6766007) for 1 minute, followed by distilled water rinsing. Finally, the sections were dehydrated in increasing concentrations of IMS (50%, 75%, and 100%) for 5 minutes each, followed by 20 minutes of clearing in xylene and mounted in xylene-based medium DePex mounting medium.

Masson's Trichrome staining

Masson's Trichrome staining was performed to assess the extent of myocardial fibrosis in the heart. The heart sections were deparaffinized and rehydrated in decreasing concentrations of IMS, followed by Bouin's (Sigma, HT110132) treatment for 2 hours. Consequently, the slides were immersed in filtered Harris' hematoxylin for 5 minutes. Thereafter, sections were immersed in red solution [0.9% (w/v) Biebrich scarlet, 0.1% Fuschin (w/v), and 1% (v/v) acetic acid] (Sigma, HT151) for 5 minutes to stain the cardiomyocytes red. After rinsing the sections, they were differentiated in 2.5% (w/v) phosphomolybdic acid (Sigma, 221856) for 15 minutes. The heart sections were then stained with aniline blue (Sigma-Aldrich, B8563) for 5 minutes to visualize the fibrotic areas. After rinsing, the sections were differentiated in 1% (v/v) glacial acetic acid for 30 seconds and subsequently rinsed with distilled water. Finally, the sections were dehydrated in decreasing concentrations of IMS and cleared in xylene. The slides were then dried and mounted in xylene-based DePex mounting medium.

TUNEL staining

A terminal deoxynucleotidyl transferase dUTP nick end labeling (TUNEL) assay was performed using the in-situ Cell Death Detection Kit (Roche, 11684795910) to detect apoptosis. The paraffin sections were deparaffinized and rehydrated as mentioned before and then treated with proteinase K in PBS (0.02 mg/mL per section) (ThermoFisher Scientific, AM2546) for 15 minutes at 37°C to allow enzymatic antigen retrieval. The tissue sections were then permeabilized with 0.1% (v/v) Triton-X and 0.1% (w/v) sodium citrate in PBS for 8 minutes and later incubated with TUNEL enzyme solution (1:100 dilution in labeling agent) at 37°C for

1 hour in a humidified chamber. Finally, the sections were mounted with a coverslip using ProLong™ Gold Antifade Mountant containing 4′,6-diamidino-2-phenylindole (DAPI) (ThermoFisher Scientific, P36935).

Immunofluorescence staining

Immunofluorescence staining on paraffin sections was conducted to assess the degree of inflammation and oxidative stress in response to UPD. The sections were deparaffinized and rehydrated as mentioned above, and then antigen retrieval was achieved via incubation with 10 mM sodium citrate pH 6.0 in the Techne Hybridizer HB-1D for 30 minutes at 95°C. The slides were quenched with 50 mM ammonium chloride for 30 minutes at room temperature. After further PBS washes, the slides were blocked with 10% normal donkey serum with 0.1% Triton-X at room temperature for one hour and then incubated with primary antibodies diluted in 5% normal donkey serum in PBS at 4°C overnight. Sections were incubated with fluorescent-conjugated secondary antibodies (Major Resources Tables) in 5% normal donkey serum in PBS for 1 hour at room temperature. The slides were then washed with PBS and mounted with ProLong™ Gold Antifade Mountant with DAPI.

Cellular immunofluorescence staining was performed on NRCMs or hiPSC-CMs following stimulation with fatty acids (FAs) for various time durations. Cells were fixed with cold methanol at 4°C for 20 minutes. Subsequently, the fixed cells were blocked with 1% (w/v) BSA in PBS and incubated with primary antibodies diluted in 0.5% (w/v) BSA in PBS at 4°C overnight. Incubation with fluorescent-conjugated secondary antibodies (Major Resources Tables) in 0.5% (w/v) BSA in PBS occurred for 1 hour at room temperature. The coverslips were then washed with 0.5% (w/v) BSA in PBS and mounted with ProLong™ Gold Antifade Mountant with DAPI.

Dihydroethidium (DHE) staining

The levels of superoxide (O_2^-) in the human heart tissues and both NRCMs and hiPSC-CMs

were evaluated using the oxidative fluorescence dye DHE (ThermoFisher Scientific, D1168). The myocardial cryosections or NRCMs or hiPSC-CMs were incubated with diluted DHE in PBS (10 μM) in a dark humidified chamber at 37°C for 30 minutes and the sections were mounted using ProLong™ Gold Antifade Mountant and immediately imaged.

Real-time quantitative polymerase chain reaction (RT-qPCR)

Total RNA from the heart tissue was extracted using TRIzol and treated with DNAse (ThermoFisher Scientific, AM1906) to eliminate genomic DNA contamination. The total RNA was converted into complementary DNA (cDNA) using LUNAscript (New England Biolabs, E3010). The qPCR reaction was conducted on 30 ng cDNA using SYBR Select PCR master mix (Applied Biosystems, 4367659) and the appropriate primers (Major Resources Tables), followed by running on the Applied Biosystems QuantStudio™ Flex System. The fold change was determined using the comparative threshold (Ct) method ($^{\triangle\triangle}$CT method) (6). The mRNA level of the target gene was normalized to the housekeeping gene 18S and PGK1 for mouse and human samples, respectively.

RNA sequencing

Total RNA quality and integrity from C57BL/6J mice hearts were first checked using a 2100 Bioanalyzer (Agilent Technologies) and the TruSeq Stranded mRNA assay (Illumina). Data analyses were performed as described previously (7). Briefly, unmapped paired-reads of 59bp were interrogated using a quality control pipeline consisting of FastQC v0.11.3 (http://www.bioinformatics.babraham.ac.uk/projects/fastqc/) and FastQ Screen v0.9.2 (https://www.bioinformatics.babraham.ac.uk/projects/fastq_screen/). Normalization and differential expression analysis were performed using DESeq2 v1.10.1 on R v3.2.3.

Total RNA from human hearts was extracted using Trizol, followed by RNA Library Preparation and NovaSeq Sequencing (GENEWIZ UK Ltd.). Samples were quantified using Qubit 4.0 Fluorometer (Life Technologies, Carlsbad, CA, USA) and RNA integrity was checked with

RNA Kit on Agilent 5300 Fragment Analyzer (Agilent Technologies, Palo Alto, CA, USA). RNA sequencing libraries were prepared using the NEBNext Ultra II RNA Library Prep Kit for Illumina following manufacturer's instructions (NEB, Ipswich, MA, USA) as described previously (1). Raw sequence data (.bcl files) generated from Illumina NovaSeq was converted into fastq files and de-multiplexed using Illumina bcl2fastq 2.20 software. DESeq2 results were filtered for genes that had a corresponding NCBI gene ID and for those genes surviving the DESeq2 multiple testing correction in the results object.

For mice and human RNA sequencing data, absolute log_2 fold change values of 1 were used to generate significantly changed pathways in ShinyGo 0.82 (http://ge-lab.org/go/).

Treatment on cells

Palmitic acid (PA, 500 µM), linoelaidic acid (LA, 400 µM), and elaidic acid (EA, 400 µM) were prepared in 0.5% (w/v) fatty acid-free bovine serum albumin (BSA) for conjugation. The molar ratio 7:1 of PA:BSA, LA:BSA, EA:BSA was utilized to mimic pathophysiologic states (8). Cells were treated with above fatty acids (FAs) for various durations depending on experimental design and purpose as stipulated in Results. The control groups received 0.5% (w/v) BSA treatment.

To determine the protective efficacy of inhibiting calpain in the presence of FA stress, hiPSC-CMs and NRCMs were treated with 20 µM calpeptin or a DMSO control as previously described (9). Conversely, to further investigate the effect of calpain on ROS and HMGB1 expression, hiPSC-CMs and NRCMs were treated with 9.5 U/mL recombinant human calpain (Sigma-Aldrich, 208713) for 18 and 8 hours respectively. To assess how reactive oxygen species affect HMGB1 in hiPSC-CMs or NRCMs, cells were exposed to FA stress concurrently in the presence or absence of 4 mM *N*-acetyl-cysteine (NAC). Alternatively, to induce ROS, hiPSC-CMs or NRCMs were exposed to 200 µM H_2O_2 for 24 hours.

Overexpression of genes

To evaluate the effect of CAST (VectorBuilder, VB221202-1131rtv) and Calpain-1 (addgene, 60941) overexpression in NRCMs, the cells were transfected with 2.5 µg human *CAST* or *CAPN1* cDNA using FuGENE® 4K Transfection reagent according to manufacturer's instructions (Promega, E5911).

In hiPSC-CM, 2.5 µg human *CAST* cDNA was transfected using Lipofectamine Stem Transfection Reagent (ThermoFisherScientific, STEM00001) following the manufacturer's instructions.

In both NRCM and hiPSC-CMs, p3xFLAG-CMV-7.1 was utilized as a control for CAST cDNA transfection.

Calpain Activity Assay

A calpain activity assay (Sigma-Aldrich, CBA054) was conducted to determine the activity of calpain enzymes and validate the efficacy of overexpressing CAST in mice according to manufacturer's guidelines. Briefly, samples were diluted to 2mg/mL and incubated with an activation buffer and calpain substrate with gentle shaking. Relative fluorescence was measured using excitation/emission wavelengths of 380 nm/500 nm.

Protein lysates extraction and immunoblotting

Protein lysates were produced utilizing Triton lysis buffer [137 mM NaCl, 20 mM Tris, 0.1% (w/v) SDS, 2 mM EDTA, 10% (v/v) glycerol, 1% (Triton-X), 25 mM glycerophosphate, protease and phosphatase inhibitor cocktail, pH 7.4]. In addition, cytosolic protein lysates were produced using a modified lysis buffer (10 mM NaCl, 20 mM Tris, 2 mM EDTA, 25 mM glycerophosphate, 2 mM $MgCl_2$, protease and phosphatase inhibitor cocktail). Protein concentration was determined using the Bradford assay (Bio-Rad, 500-0006); immunoblotting was performed using approximately 30µg of protein lysate. The primary antibodies (Major

Resources Table) and both anti-mouse and anti-rabbit HRP-conjugated secondary antibodies were diluted in 5% (w/v) fat-free milk in TBS-T. Amersham ECL Prime (RPN2236) and Select (RPN2235) detection reagents were utilized alongside the ChemiDoc MP System (BioRad) to visualise protein bands via chemiluminescence.

THP-1 polarization

For flow cytometry analysis, THP-1 cells were seeded at a density of 100,000 cells/cm^2. THP-1 cells were primed with 150 nM phorbol 12-myristate 13-acetate (PMA, Sigma-Aldrich, P8139) for 24 hours. Following a 24-hour recovery period, differentiated THP-1 cells were treated with conditioned media as indicated in Results for 24 hours. Alternatively, M1 and M2 macrophage controls were polarized via incubation with 100 ng/mL lipopolysaccharide (Sigma-Aldrich, LPS25) and 100 ng/mL IFNγ (Biotechne, 285-IF) or, 20 ng/mL recombinant human IL-4 (Biotechne, 204-IL) respectively for 24 hours.

Macrophage isolation from the heart

Macrophages were isolated from the hearts of UPD fed mice with CAST overexpression or following receipt of calpeptin treatment for flow cytometric analysis as previously described (10). Briefly, hearts were dissected into small pieces and digested in DMEM with 600 U/mL Collagenase type 2 (Worthington, LS004174) and 60 U/mL DNase I (Worthington, LS002139). The tissue was filtered through a 45 μM strainer and the resulting pass through was neutralized with 0.1% BSA (w/v) 1% FBS (v/v) HBSS and red blood cells (RBCs) lysed in an RBC buffer [150 mM NH_4Cl, 10 mM $NaHCO_3$, 1.2 mM EDTA-disodium].

Flow cytometry

Flow cytometry was employed to perform immunofluorescence-based analysis of single cell THP-1 derived macrophages and macrophages isolated from murine hearts. Suspensions of $1x10^6$ cells prepared in 100 μL PBS were stained with Zombie Aqua™ (BioLegend, 423101) (1:500 and 1:1000 respectively). Cells were blocked with CD16/32 (1:100) for 10 minutes on

ice and subsequently incubated with CD86 and CD206 antibodies (Major Resources Tables) for 30 minutes at room temperature in flow cytometry staining buffer (ThermoFisher Scientific, 00-4222-26). In addition to CD86 and CD206, tissue derived macrophages were also stained with CD45, F4/80 and Ly6G antibodies (Major Resources Tables). Stained cells were fixed with 2% PFA and following further washing were resuspended in 100 µL PBS. Samples were run on BD Biosciences LSRFortessa with BDFACS software. Single antibody controls were utilized to generate a compensation matrix. Data was acquired utilizing the following channels: CD86 (M1) 640 (780/60), CD206 (M2) 640 (670/30), Zombie Aqua™ (dead cells) 405 (450/50), CD45 (leukocytes) 488 (780/60), F4/80 (macrophages) 488 (586/15), Ly6G (neutrophils) 640 (730/45). Data was analyzed utilized FlowJo v10 software with dead cells being excluded. For tissue derived macrophages, Ly6G positive cells were also excluded.

Imaging of histology

H&E and Masson's Trichrome-stained slides were imaged using a 3D-Histech Pannoramic-250 microscope slide-scanner using a Zeiss 20x/0.80 Plan Apochromat objective. Snapshots of the scanned slides were taken using the Case Viewer software (3D-Histech). Fluorescent-labeled slides for TUNEL, DHE, and immunofluorescence staining were imaged using a Zeiss Axioimager D2 upright microscope with Zeiss LD Plan-Neofluar objectives (x20, x40) and captured using a Coolsnap HQ2 camera (Photometrics) through Micromanager software v1.4.23. Specific band-pass filter sets for DAPI, FITC, and Texas Red were used to prevent bleeding from one channel to the next. Images were then further processed and analyzed using Image J software.

Statistical analysis

Data are presented as bar/dot plots showing mean ± SEM. Where sample sizes were ≥5, the Shapiro-Wilk test was conducted to first determine whether data was normally distributed. Normally distributed data sets were analyzed using ordinary two-way ANOVA followed by appropriate post-hoc tests, whereas comparisons between two groups were performed using

2-tailed Student's t tests. The non-parametric equivalents were utilized for skewed data and data sets where sample sizes were <5. Mann Whitney tests were used for non-parametric two group comparisons. Statistical analyses were performed using the GraphPad Prism 10 software, and *p*<0.05 was considered statistically significant. The sample size corresponds to biological replicates and is specified for each experiment within the figure legend.

KEY RESOURCE TABLE

**ANIMALS**		
**SPECIES**	**STRAIN**	**SOURCE**
Mouse	C57BL/6J	Envigo
Rat	Sprague-Dawley	Envigo
**CULTURED CELLS**		
**CELL**	**SOURCE**	**IDENTIFIER**
NRCM	Primary cells	N/A
hiPSC	Invitrogen	hiPSC 02C9
THP-1	ATCC	TIB-202
**qPCR PRIMERS**		
**PRODUCT**	**SOURCE**	**IDENTIFIER**
HMGB1 (Hs)	Qiagen	QT01002190
PGK1 (Hs)	Qiagen	QT00013776
Hmgb1 (Ms)	Qiagen	QT00247786
Tnfα (Ms)	Qiagen	QT00116564
Il1β (Ms)	Qiagen	QT01048355
Il6 (Ms)	Qiagen	QT00098875
Crp (Ms)	Qiagen	QT00255444
Arg1 (Ms)	Qiagen	QT00134288

Il10 (Ms)	Qiagen	QT00106169
Gdf15 (Ms)	Qiagen	QT00124481
Sod2 (Ms)	Qiagen	QT00161707
Gpx1 (Ms)	Qiagen	QT01195936
Nppb (Ms)	Qiagen	QT00107541
Col1a2 (Ms)	Qiagen	QT02325736
Col3a1 (Ms)	Qiagen	QT00297094
18s (Ms)	Qiagen	QT02448075

**IMMUNOBLOTTING ANTIBODIES**			
**ANTIBODIES**	**DILUTION**	**SOURCE**	**IDENTIFIER**
HMGB1	1:1000	Abcam	ab18256
CAST	1:1000	Proteintech	12250-1-AP
calpain-1	1:1000	Proteintech	10538-1-AP
calpain-2	1:1000	Proteintech	11472-1-AP
GAPDH	1:1000	Abcam	mAbcam 9484
Beta Actin (β-Actin)	1:1000	Proteintech	66009-1-Ig
HRP-linked anti-mouse	1:1000	Cell Signaling Technology	7076
HRP-linked anti-rabbit	1:1000	Cell Signaling Technology	7074

**IMMUNOFLUROSCENCE ANTOBODIES**			
**ANTIBODIES**	**DILUTION**	**SOURCE**	**IDENTIFIER**
HMGB1	1:100-1:1000	Abcam	ab18256
4HNE	1:50	Abcam	ab46545

CD86	1:100	Cell Signaling Technology	19589S
Mac3	1:300	BioLegend	108502
Alpha-Actinin (α-Actinin)	1:100	Sigma-Aldrich	A7811
Anti-rabbit AlexaFluor 594	1:250-1:500	Jackson ImmunoResearch	711-585-152
Anti-rabbit AlexaFluor 488	1:250	Jackson ImmunoResearch	711-545-152
Anti-mouse AlexaFluor 488	1:250	Jackson ImmunoResearch	715-546-151
Anti-rat AlexaFluor 594	1:200	Jackson ImmunoResearch	712-585-153
Anti-mouse AlexaFluor 594	1:1000	Jackson ImmunoResearch	715-586-151
**FLOW CYTOMETRY ANTOBODIES**			
Anti-mouse CD16/32	1:500	BioLegend	101319
APC/Cy7 anti-mouse CD86	1:100	BioLegend	105029
APC anti-mouse CD206	1:100	BioLegend	141707
PE anti-mouse F4/80	1:50	BioLegend	123109
AF700 anti-mouse Ly6G	1:200	BioLegend	127621
PE/Cy7 anti-mouse CD45	1:100	BioLegend	103114

Zombie Aqua™ Fixable Viability Kit	1:500	BioLegend	423101

OTHER MAJOR RESOURCES		
**PRODUCT**	**SOURCE**	**IDENTIFIER**
RPMI 1640	Gibco	A10491
CytoTune iPS Programming Kit	Life Technologies	A16518
DMEM/F-12 GlutaMAX	Life Technologies	31331028
mTeSR Plus	Stem Cell technologies	100-0276
Thiazovivin	Scientific Laboratory Supplies	420220
CHIR99021	Merck Millipore	361559
IWP2	Merck Millipore	681671
RPMI 1640 GlutaMAX	Life Technologies	72400021
B27 Minus Insulin Supplement	Life Technologies	A1895601
B27 Insulin Supplement	Life Technologies	17504044
DMEM	Gibco	11966-025
M199	Gibco	31150-022
Collagenase A	Roche	10103586001
BRDU	Sigma-Aldrich	B5002
Pancreatin	Sigma-Aldrich	P3292
Collagenase Type 2	Worthington	LS004174
DNase I	Worthington	LS002139
Calpeptin	MedChemexpress	HY-100223
Recombinant human calpain	Sigma-Aldrich	208713
N-acetyl-cysteine (NAC)	Sigma-Aldrich	A9165
Lipopolysaccharide (LPS)	Sigma-Aldrich	LPS25

Recombinant Human IL-4	R&D Systems	204-IF
Phorbol 12-Myristate 13-Acetate (PMA)	Sigma-Aldrich	P8139
Recombinant Human IFNγ	R&D Systems	285-IF
Palmitic acid (PA)	Sigma-Aldrich	P5585
Elaidic acid (EA)	Sigma-Aldrich	E4637
Linoelaidic acid (LA)	BroadPharm	BP-40891
FuGene®	Promega	E5911
Lipofectamine Stem Reagent	ThermoFisher Scientific	STEM00001
TRizol reagent	ThermoFisher Scientific	15596018
DNA-free Kit	ThermoFisher Scientific	AM1906
LunaScript	New England Biolabs	E3010L
Power SYBR Green PCR master mix	Applied Biosystems	4367659
ECL Select Western Blotting detection reagent	Amersham	RPN2235
ECL Prime Western Blotting detection reagent	Amersham	RPN2236
DHE	ThermoFisher Scientific	D1168
TUNEL	Roche	11684795910
ProLong™ Gold Antifade Mountant	ThermoFisher Scientific	P36935
Flow Cytometry Staining Buffer	ThermoFisher Scientific	00-4222-26
InnoZyme Calpain 1/2 Activity Assay Kit	Sigma-Aldrich	CBA054

References

1. Fonseka O et al. XBP1s-EDEM2 Prevents the Onset and Development of HFpEF by Ameliorating Cardiac Lipotoxicity. *Circulation.* 2025.
2. Ruiz-Velasco A et al. Targeting mir128-3p alleviates myocardial insulin resistance and prevents ischemia-induced heart failure. *Elife.* 2020;9:e54298.
3. Schiattarella GG et al. Nitrosative stress drives heart failure with preserved ejection fraction. *Nature.* 2019;568(7752):351-6.
4. Gautheron F et al. Bone marrow-derived extracellular vesicles carry the TGF-beta signal transducer Smad2 to preserve hematopoietic stem cells in mice. *Cell Death Discov.* 2023;9(1):117.
5. Ma Y, Gao M, and Liu D. N-acetylcysteine Protects Mice from High Fat Diet-induced Metabolic Disorders. *Pharm Res.* 2016;33(8):2033-42.
6. Livak KJ, and Schmittgen TD. Analysis of relative gene expression data using real-time quantitative PCR and the 2– ΔΔCT method. *methods.* 2001;25(4):402-8.
7. Ruiz-Velasco A et al. Restored autophagy is protective against PAK3-induced cardiac dysfunction. *Iscience.* 2023;26(6).
8. Kaur N et al. Paracrine signal emanating from stressed cardiomyocytes aggravates inflammatory microenvironment in diabetic cardiomyopathy. *iScience.* 2022;25(3):103973.
9. Liu W et al. Metabolic stress-induced cardiomyopathy is caused by mitochondrial dysfunction due to attenuated Erk5 signaling. *Nature communications.* 2017;8(1):494.
10. Xiao H et al. IL-18 cleavage triggers cardiac inflammation and fibrosis upon beta-adrenergic insult. *Eur Heart J.* 2018;39(1):60-9.

Supplemental Tables

12 weeks	Chow	45% HFD	45% HFD+STZ	60% HFD+L-NAME	UPD
FS (%)	38.77±1.87	37.59±0.76	38.05±2.07	38.74±1.19	30.28±0.98*
EF (%)	76.59±2.15	75.79±0.88	75.99±2.44	76.85±1.40	65.97±1.40*
sD(mm)	2.73±0.10	2.69±0.05	2.48±0.09	2.49±0.03	3.15±0.10
dD (mm)	4.45±0.06	4.32±0.05	4.01±0.14*	4.07±0.05*	4.51±0.10
dIVS (mm)	0.74±0.02	0.77±0.03	0.83±0.03	0.81±0.03	0.82±0.01*
sIVS (mm)	0.94±0.03	1.05±0.03	1.05±0.07	1.04±0.04	1.13±0.03*
dPW (mm)	0.60±0.02	0.78±0.03*	0.89±0.02*	0.89±0.03*	0.82±0.02*
sPW (mm)	0.89±0.02	1.01±0.05	1.17±0.11*	1.24±0.07*	1.10±0.04*
IVRT (ms)	8.88±0.35	10.30±0.63	13.25±0.63	16.43±0.65*	16.13±0.74*
E/A	1.38±0.03	1.40±0.04	1.53±0.13	2.03±0.09*	1.82±0.06*
HR	469.13±13.85	464.40±11.44	500.25±20.99	499.14±11.43	416.88±6.20*

Supplemental Table 1. Echocardiographic assessments of C57BL/6J mice (male) following 12 weeks of chow diet or various dietary stresses. High-fat diet (HFD), streptozotocin (STZ), N(ω)-nitro-L-arginine methyl ester (L-NAME), ultra-processed diet (UPD), fractional shortening (FS%), ejection fraction (EF%), left ventricular end-systolic diameter (sD), left ventricular end-diastolic diameter (dD), end-diastolic interventricular septum thickness (dIVS), end-systolic interventricular septum thickness (sIVS), end-diastolic left ventricular posterior wall thickness (dPW), end-systolic left ventricular posterior wall thickness (sPW), isovolumic relaxation time (IVRT), heart rate (HR) (n=12, 10, 4, 7, 8 mice respectively). Data are presented as mean ± S.E.M. p values were calculated using a Kruskal Wallis with Dunn's post-hoc tests. * $p<0.05$ compared to chow group.

4 weeks	Chow	UPD
FS (%)	39.51±1.88	37.55±1.57
EF (%)	77.24±2.00	75.13±1.90
sD(mm)	2.49±0.08	2.36±0.10
dD (mm)	4.11±0.04	3.76±0.10*
dIVS (mm)	0.71±0.03	0.72±0.03
sIVS (mm)	0.92±0.01	0.94±0.02
dPW (mm)	0.58±0.01	0.76±0.02*
sPW (mm)	0.88±0.03	1.02±0.03*
IVRT (ms)	9.36±0.34	13.25±0.65*
E/A	1.42±0.04	1.78±0.11*
HR	446.36±10.59	438.67±10.42

Supplemental Table 2. Echocardiographic assessments of C57BL/6J mice, mixed sexes, following 4 weeks feeding with chow or ultra-processed diet (UPD). Fractional shortening (FS%), ejection fraction (EF%), left ventricular end-systolic diameter (sD), left ventricular end-diastolic diameter (dD), end-diastolic interventricular septum thickness (dIVS), end-systolic interventricular septum thickness (sIVS), end-diastolic left ventricular posterior wall thickness (dPW), end-systolic left ventricular posterior wall thickness (sPW), isovolumic relaxation time (IVRT), heart rate (HR) (n=11, 12 mice respectively). Data are presented as mean ± S.E.M. *p* values were calculated using a 2-tailed Student's t tests. * $p<0.05$ compared to chow group.

	Chow		UPD	
	**AAV9-cTnT-e*Gfp***	**AAV9-cTnT-*CAST***	**AAV9-cTnT-e*Gfp***	**AAV9-cTnT-*CAST***
FS (%)	41.93±0.97	45.46±0.65	32.23±1.33*	40.05±1.10$^{+\#}$
EF (%)	80.28±1.03	83.74±0.59	68.48±1.80*	78.24±1.09$^{+\#}$
sD(mm)	2.38±0.06	2.08±0.05	2.84±0.14*	2.42±0.05$^{\#}$
dD (mm)	4.09±0.07	3.82±0.05	4.16±0.14	4.03±0.02
dIVS (mm)	0.73±0.01	0.74±0.01	0.83±0.01*	0.76±0.01$^{\#}$
sIVS (mm)	0.94±0.01	0.94±0.01	1.11±0.02*	0.95±0.01$^{\#}$
dPW (mm)	0.59±0.01	0.58±0.01	0.85±0.02*	0.65±0.01$^{+\#}$
sPW (mm)	0.90±0.01	0.85±0.03	1.13±0.04*	0.95±0.02$^{\#}$
IVRT (ms)	9.19±0.28	9.83±0.31	16.25±0.58*	11.91±0.25$^{+\#}$
E/A	1.35±0.05	1.41±0.05	1.70±0.06*	1.37±0.05^{+}
HR	452.73±9.11	463.5±23.13	426.08±8.59	445.64±8.37
HW/TL	6.94±0.39	7.90±0.77	8.66±0.38*	7.07±0.43
LW/TL	6.39±0.36	7.82±0.53	9.55±0.24*	7.80±0.15$^{\#}$

Supplemental Table 3. Echocardiographic assessments of mice, mixed sexes, overexpressing *CAST* with ultra-processed diet (UPD) feeding. Fractional shortening (FS%), ejection fraction (EF%), left ventricular end-systolic diameter (sD), left ventricular end-diastolic diameter (dD), end-diastolic interventricular septum thickness (dIVS), end-systolic interventricular septum thickness (sIVS), end-diastolic left ventricular posterior wall thickness (dPW), end-systolic left ventricular posterior wall thickness (sPW), isovolumic relaxation time (IVRT), heart rate (HR), heart weight/tibia length (HW/TL), and lung weight/tibia length (LW/TL) (n=8, 6, 8, 10 mice respectively). Data are presented as mean ± S.E.M. *p* values were calculated using a two-way ANOVA with Šidák post-hoc tests. $^{*+}$ $p<0.05$ compared to respective chow fed cohort, $^{\#}$ $p<0.05$ compared to AAV9-cTnT-*eGFP* with UPD group.

	Chow		UPD	
	**Vehicle**	**Calpeptin**	**Vehicle**	**Calpeptin**
FS (%)	46.49±1.08	44.97±0.55	31.70±0.18*	38.80±0.63$^{+\#}$
EF (%)	84.62±0.95	83.32±0.50	68.13±0.25*	77.05±0.69$^{+\#}$
sD(mm)	2.19±0.05	2.25±0.04	3.34±0.05*	2.53±0.05$^{+\#}$
dD (mm)	4.09±0.03	4.09±0.03	4.88±0.07*	4.14±0.04$^{\#}$
dIVS (mm)	0.71±0.01	0.74±0.01	0.82±0.01	0.77±0.01
sIVS (mm)	0.87±0.02	0.93±0.01	1.11±0.04	0.96±0.02
dPW (mm)	0.57±0.01	0.59±0.01	0.90±0.01	0.68±0.02
sPW (mm)	0.91±0.01	0.95±0.02	1.17±0.03	0.98±0.02
IVRT (ms)	9.50±0.29	9.50±0.29	17.50±0.65*	12.50±0.43$^{+\#}$
E/A	1.33±0.02	1.43±0.04	1.96±0.03*	1.40±0.06$^{\#}$
HR	437.75±12.90	474.25±14.83	422.25±7.74	439.00±6.77
HW/TL	6.75±0.23	7.86±0.49	9.45±0.45*	7.12±0.43$^{\#}$
LW/TL	6.61±0.19	7.63±0.24	9.85±0.16*	9.09±0.94

Supplemental Table 4. Echocardiographic assessments of mice, mixed sexes, receiving calpeptin administration with ultra-processed diet (UPD) feeding. Fractional shortening (FS%), ejection fraction (EF%), left ventricular end-systolic diameter (sD), left ventricular end-diastolic diameter (dD), end-diastolic interventricular septum thickness (dIVS), end-systolic interventricular septum thickness (sIVS), end-diastolic left ventricular posterior wall thickness (dPW), end-systolic left ventricular posterior wall thickness (sPW), isovolumic relaxation time (IVRT), heart rate (HR), heart weight/tibia length (HW/TL), and lung weight/tibia length (LW/TL) (n=4, 4, 4, 6 mice respectively). Data are presented as mean ± S.E.M. *p* values were calculated using a two-way ANOVA with Šidák post-hoc tests. $^{*+}$ $p<0.05$ compared to respective chow fed cohort, $^{\#}$ $p<0.05$ compared to UPD without treatment group.

	UPD	UPD+NAC
FS (%)	35.02±3.74	41.64±1.38*
EF (%)	72.54±4.26	80.10±1.41*
sD(mm)	3.11±0.35	2.47±0.07*
dD (mm)	4.79±0.30	4.23±0.04*
dIVS (mm)	0.81±0.06	0.72±0.05*
sIVS (mm)	1.09±0.07	0.98±0.04
dPW (mm)	0.78±0.06	0.69±0.04*
sPW (mm)	1.08±0.07	1.01±0.05
IVRT (ms)	18.75±3.31	13.00±0.82*
E/A	1.83±0.26	1.39±0.05*
HR	448.00±20.50	459.75±20.60
HW/TL	7.28±1.34	5.38±0.35*
LW/TL	8.72±1.21	7.83±0.49

Supplemental Table 5. Echocardiographic assessments of C57BL/6J mice, mixed sexes, receiving NAC treatment with UPD feeding. Fractional shortening (FS%), ejection fraction (EF%), left ventricular end-systolic diameter (sD), left ventricular end-diastolic diameter (dD), end-diastolic interventricular septum thickness (dIVS), end-systolic interventricular septum thickness (sIVS), end-diastolic left ventricular posterior wall thickness (dPW), end-systolic left ventricular posterior wall thickness (sPW), isovolumic relaxation time (IVRT), heart rate (HR), heart weight/tibia length (HW/TL), and lung weight/tibia length (LW/TL) (n=4 mice). Data are presented as mean ± S.E.M. *p* values were calculated using a Mann-Whitney test. * $p<0.05$ compared to UPD group.

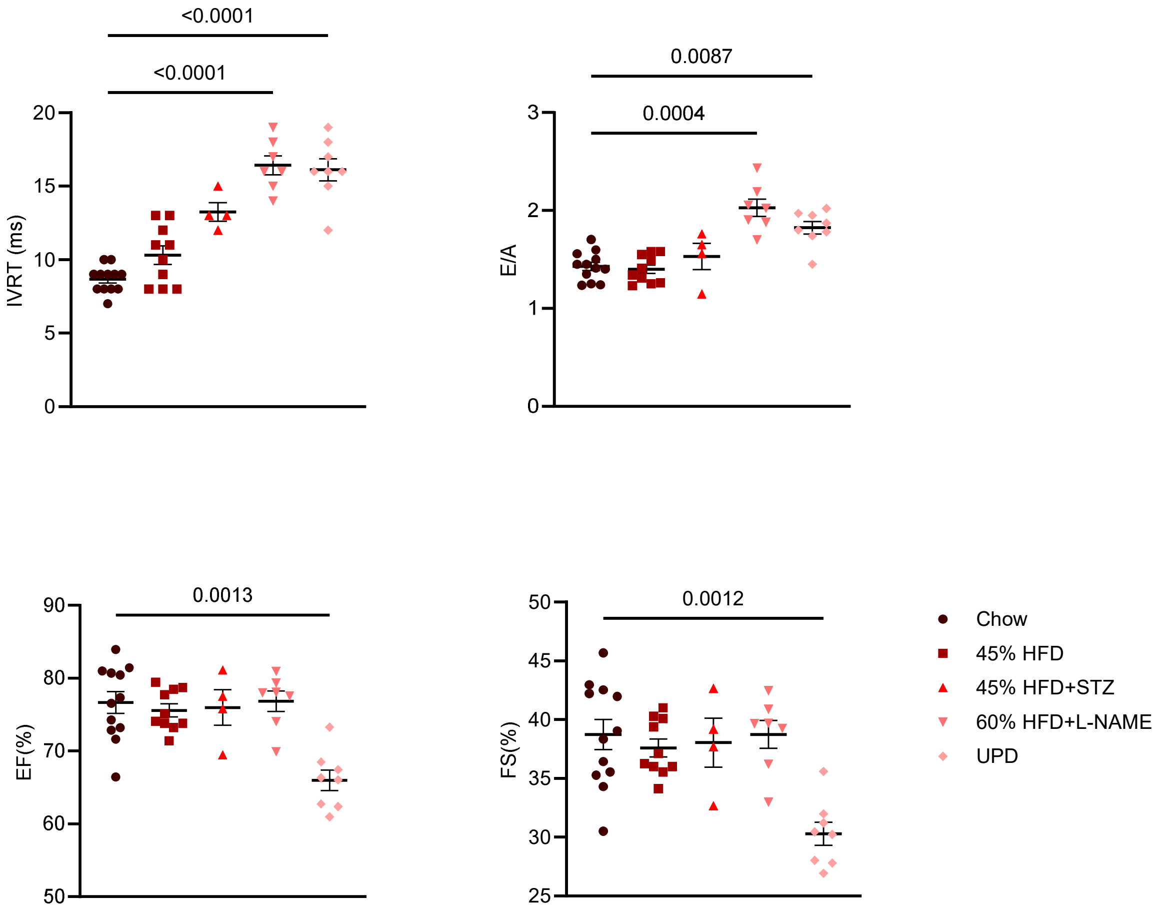

Supplemental Figure 1. Ultra-processed diet (UPD) feeding causes cardiac dysfunction earlier than other models of metabolic disorders

Isovolumic relaxation time (IVRT), ratio of peak velocity blood flow from left ventricular relaxation in early diastole to that in late diastole (E/A), percentage of ejection fraction (EF%) and fractional shortening (FS%) following 12 weeks of chow feeding or metabolic stress (n=4-12 mice). Data are presented as mean ±SEM. *p* values were calculated using Kruskal Wallis with Dunn's post-hoc.

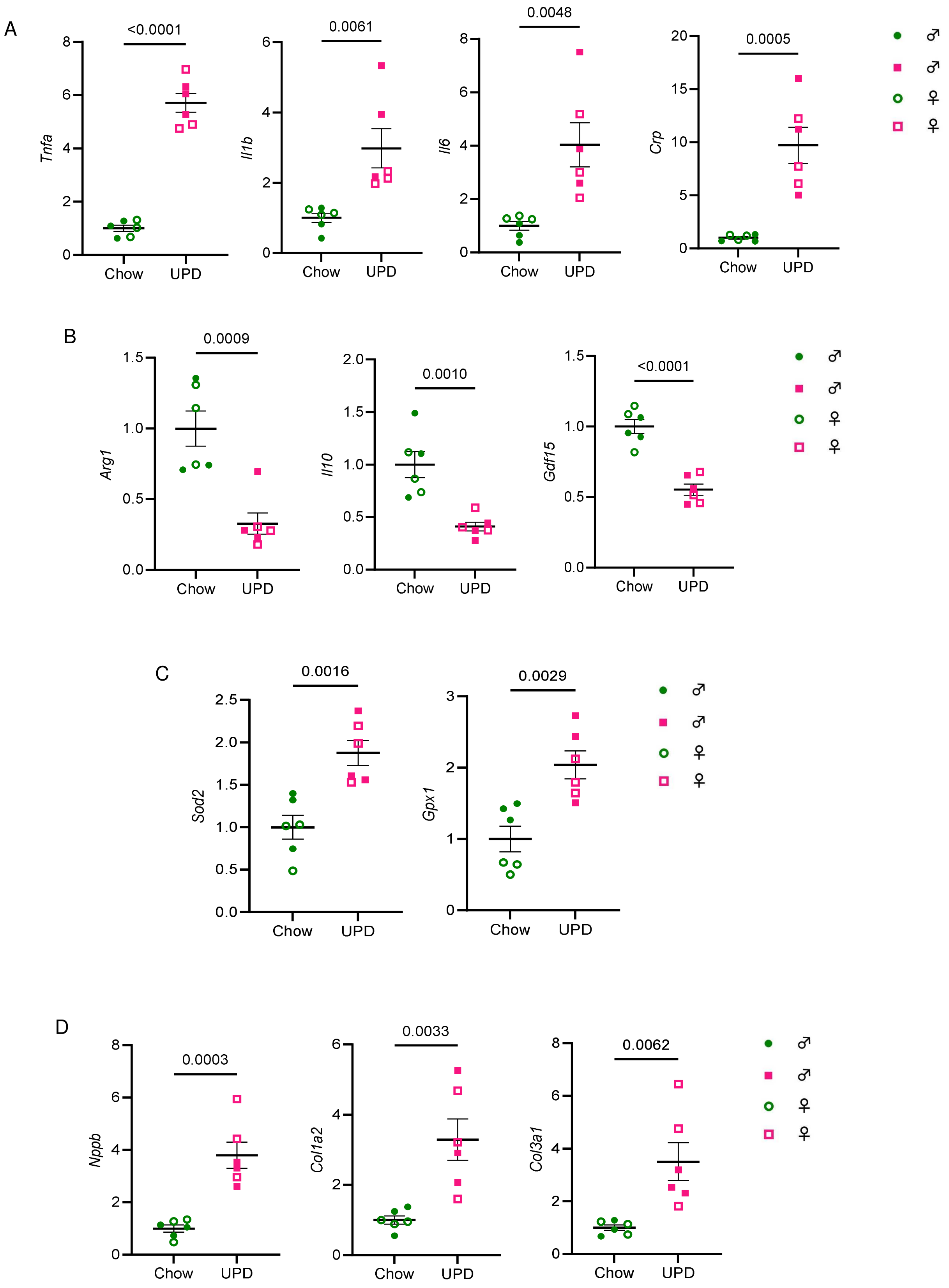

Supplemental Figure 2. Ultra-processed diet (UPD) induces cardiac inflammation, oxidative stress and pathological remodeling

Quantitative PCR of pro-inflammatory (**A**), anti-inflammatory (**B**) and antioxidant genes (**C**), and those associated with cardiac hypertrophy and fibrosis (**D**) (n=6 hearts). Data are presented as mean ±SEM with solid and hollow symbols representing male and female mice respectively. *p* values were calculated using 2-tailed Student's t tests (**A-D**).

Supplemental Figure 3

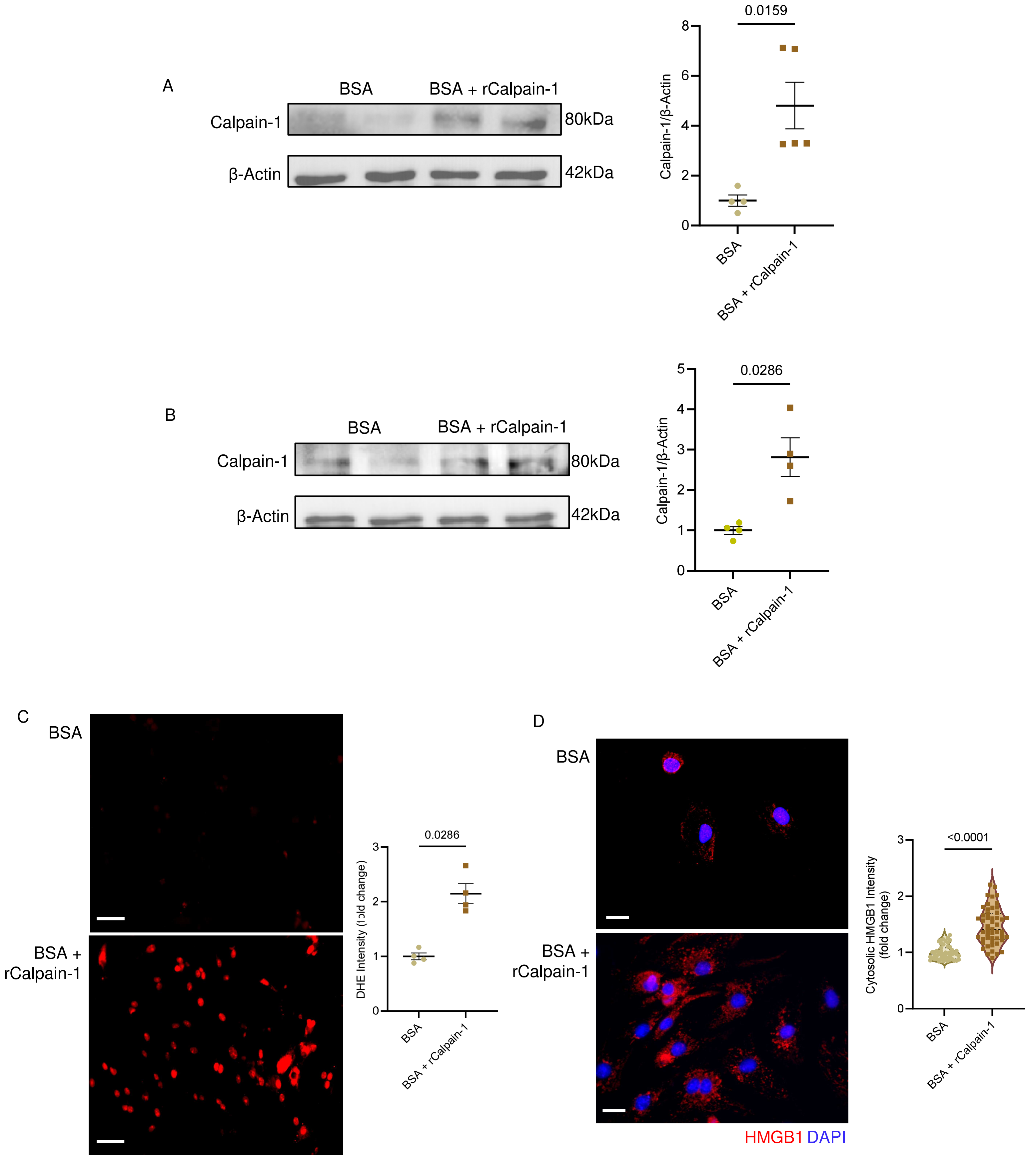

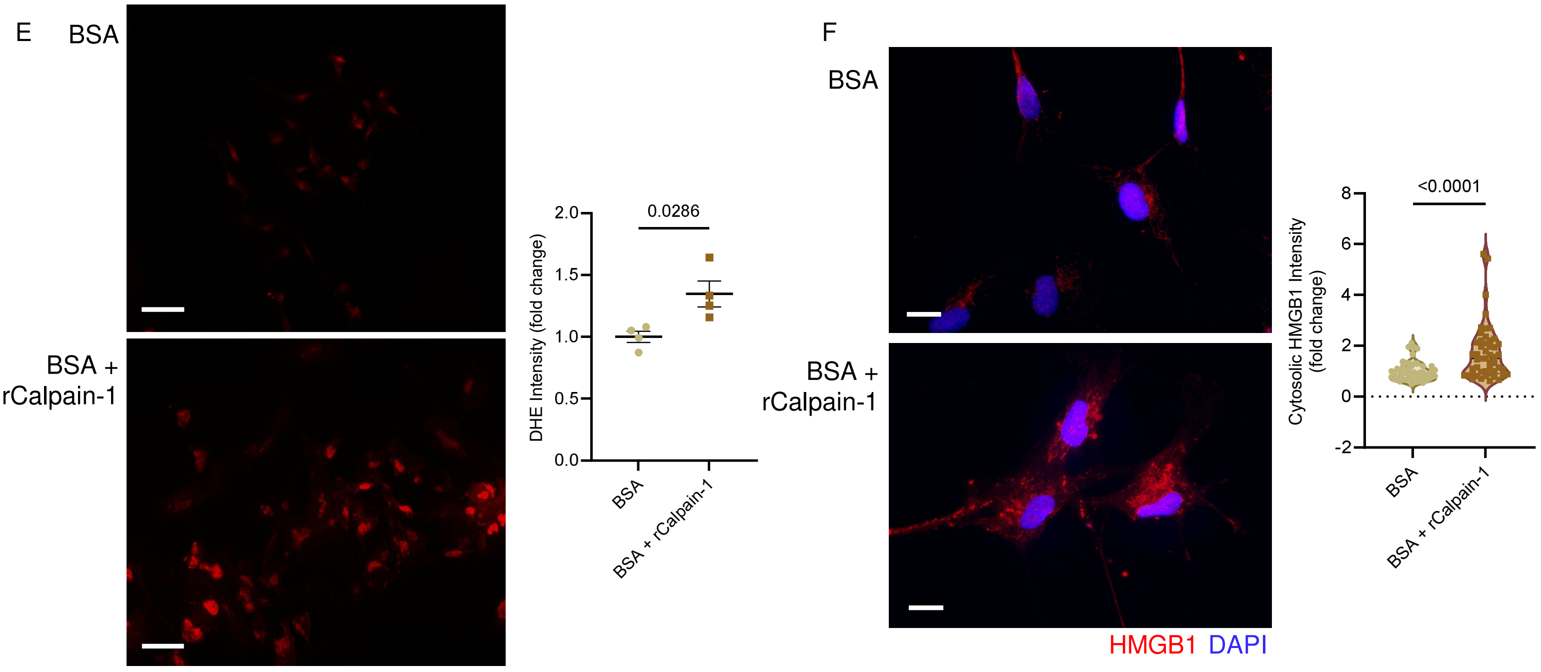

Supplemental Figure 3. Recombinant calpain-1 protein induces reactive oxygen species (ROS) generation and cytosolic HMGB1 upregulation

A-B, Immunoblots and quantification validating an increase in calpain-1 in NRCMs (n=4-5 experiments) (**A**) and hiPSC-CMs (n=4 experiments) (**B**) following treatment with BSA concurrently with or without 9.5 U/mL recombinant calpain-1 (rCalpain-1) protein for 8 and 18 hours respectively, where β-Actin is used as a loading control. **C-D**, Representative images and quantification of DHE (scale bar=50 μm) (n=4 experiments) (**C**), and cytosolic HMGB1 staining (red) with DAPI stained nuclei (blue) (scale bar=20 μm) (n=50 cells across 3 experiments) (**D**) in NRCMs stimulated for 8 hours with BSA with or without concurrent 9.5 U/mL recombinant Calpain-1 protein. **E-F**, Representative images and quantification of DHE (scale bar=50 μm) (n=4 experiments) (**E**), and cytosolic HMGB1 staining (red) with DAPI stained nuclei (blue) (scale bar=20 μm) (n=50 cells across 3 experiments) (**F**) in hiPSC-CMs stimulated for 18 hours with BSA with or without concurrent 9.5 U/mL recombinant calpain. Data are presented as mean ±SEM. *p* values were calculated using Mann-Whitney tests (**A-F**).

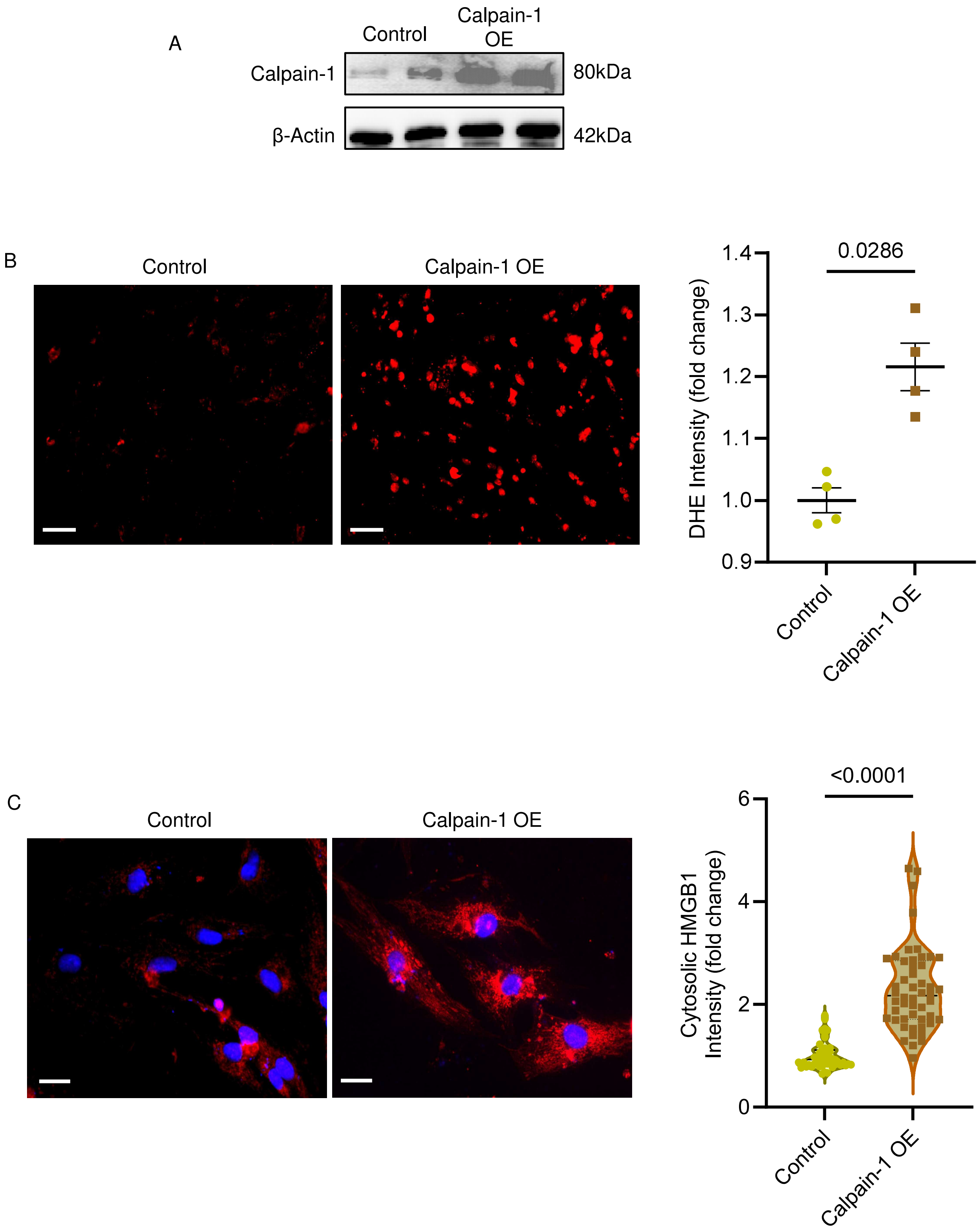

Supplemental Figure 4. Calpain-1 overexpression induces reactive oxygen species (ROS) generation and cytosolic HMGB1 upregulation

A, Immunoblots validating an increase in calpain-1 expression following transfection of human *CAPN1* cDNA in NRCMs, where β-Actin is used as a loading control (4 experiments conducted) **B-C**, Representative images and quantification of DHE (scale bar=50 µm) (n=4 experiments) (**B**), and cytosolic HMGB1 staining (red) with DAPI stained nuclei (blue) (scale bar=20 µm) (n=50 cells across 3 experiments) (**C**) in NRCMs stimulated for 8 hours with BSA with or without calpain-1 overexpression. Data are presented as mean ±SEM. *p* values were calculated using Mann-Whitney tests (**B-C**).

A

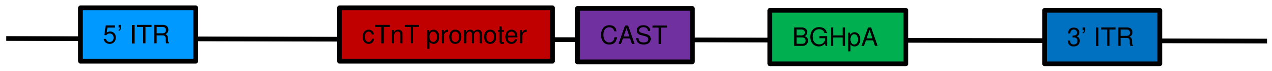

B

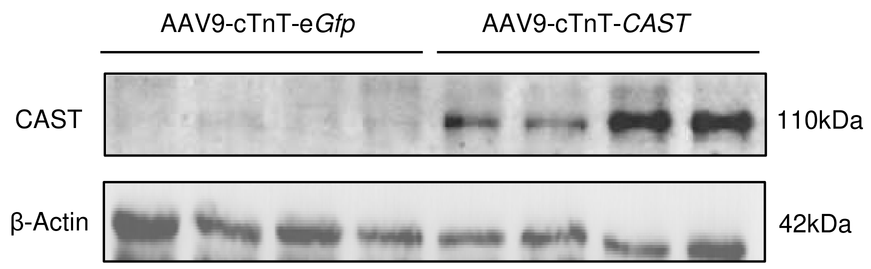

Supplemental Figure 5. AAV9 mediated cardiac CAST overexpression in mice

A, Schematic of the AAV9 viral vector construct of AAV9-cTnT-*CAST*. **B**, Immunoblots validating an increase in CAST expression using AAV9-cTnT-*CAST*, where β-Actin is used as a loading control.

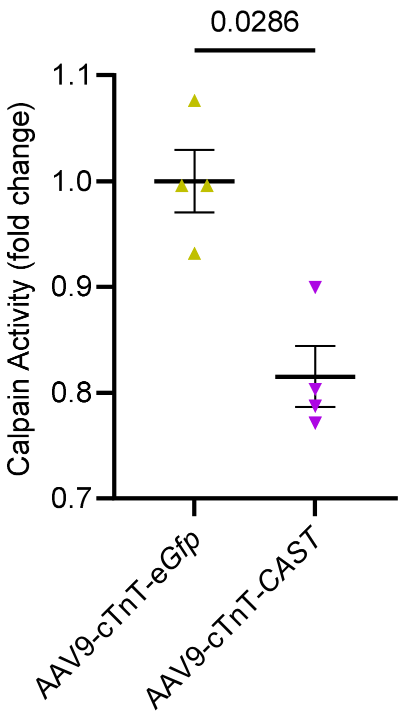

Supplemental Figure 6. AAV9 mediated cardiac CAST overexpression in mice inhibits cardiac calpain activity

Calpain activity assay validating AAV9 mediated cardiac CAST overexpression resulted in the inhibition of cardiac calpain activity (n=4 mice). Data are presented as mean ±SEM. *p* values were calculated using a Mann Whitney test.

Supplemental Figure 7

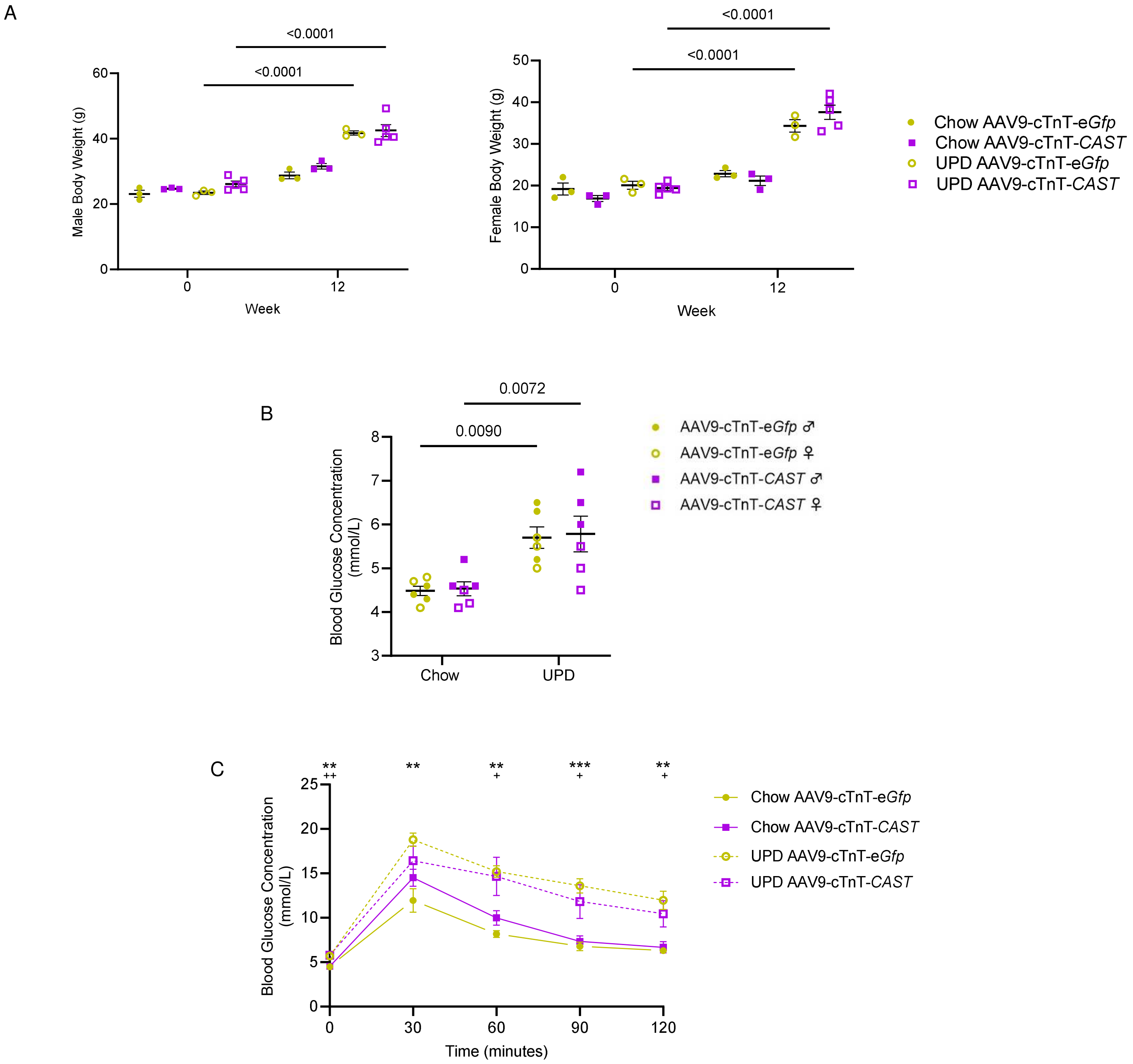

Supplemental Figure 7. Cardiac CAST overexpression does not prevent ultra-processed diet (UPD) induced metabolic disorder

A, Male and female mice body weights at the beginning of the study and following 12 weeks of chow or UPD diet (n=3-5 mice). **B**, Fasting blood glucose, and **C**, glucose tolerance test following 12 weeks of chow or UPD feeding (n=6 mice). Data are presented as mean ±SEM with solid and hollow symbols representing male and female mice respectively (**A-B**). *p* values were calculated using two-way ANOVA with Šidák post-hoc test (**A-C**). Significant differences in blood glucose levels at any timepoint between UPD AAV9-cTnT-e*Gfp* (*) and AAV9-cTnT-*CAST* (⁺) mice and their respective chow-fed cohorts are presented.

Supplemental Figure 8

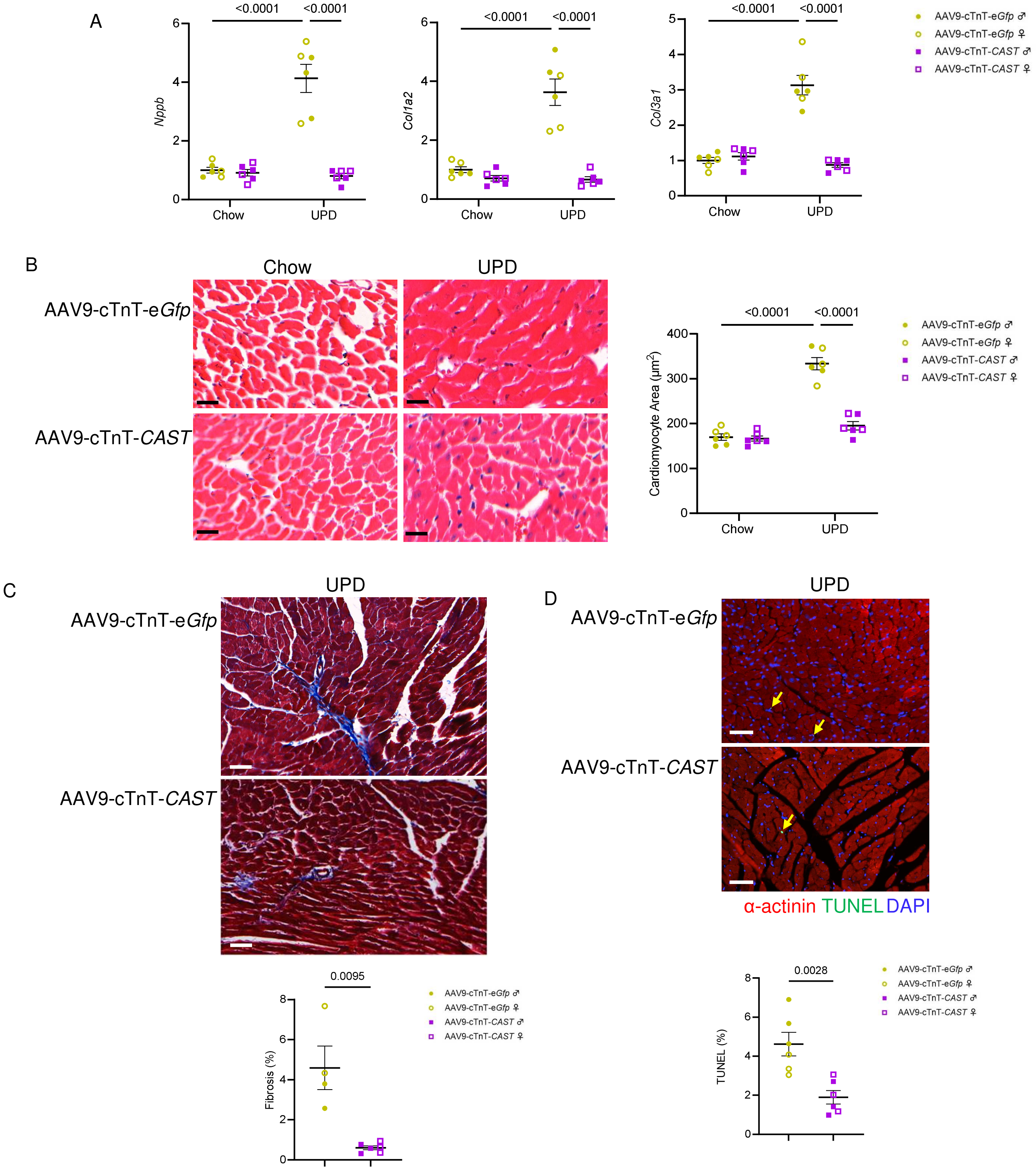

Supplemental Figure 8. Cardiac CAST overexpression protects the heart against ultra-processed diet (UPD) induced pathological remodeling

A, Quantitative PCR of genes related to cardiac hypertrophy, *Nppb*, and fibrosis, *Col1a2* and *Col3a1* (n=6 hearts). **B**, Hematoxylin and eosin staining for cross-sectional area of cardiomyocytes (scale bar=50 μm) (n=6 hearts). **C**, Masson's Trichrome staining of interstitial fibrosis in the heart (scale bar=20 μm) (n=4-6 hearts). **D**, Representative images and quantification of TUNEL staining (scale bar=50 μm, arrows indicate TUNEL-positive cardiomyocytes), DAPI stained nuclei (blue) (n=6 hearts). Data are presented as mean ±SEM with solid and hollow symbols representing male and female mice respectively. *p* values were calculated using 2-tailed Student's t tests (**A, C-D**) and two-way ANOVA with Šidák post-hoc test (**B**).

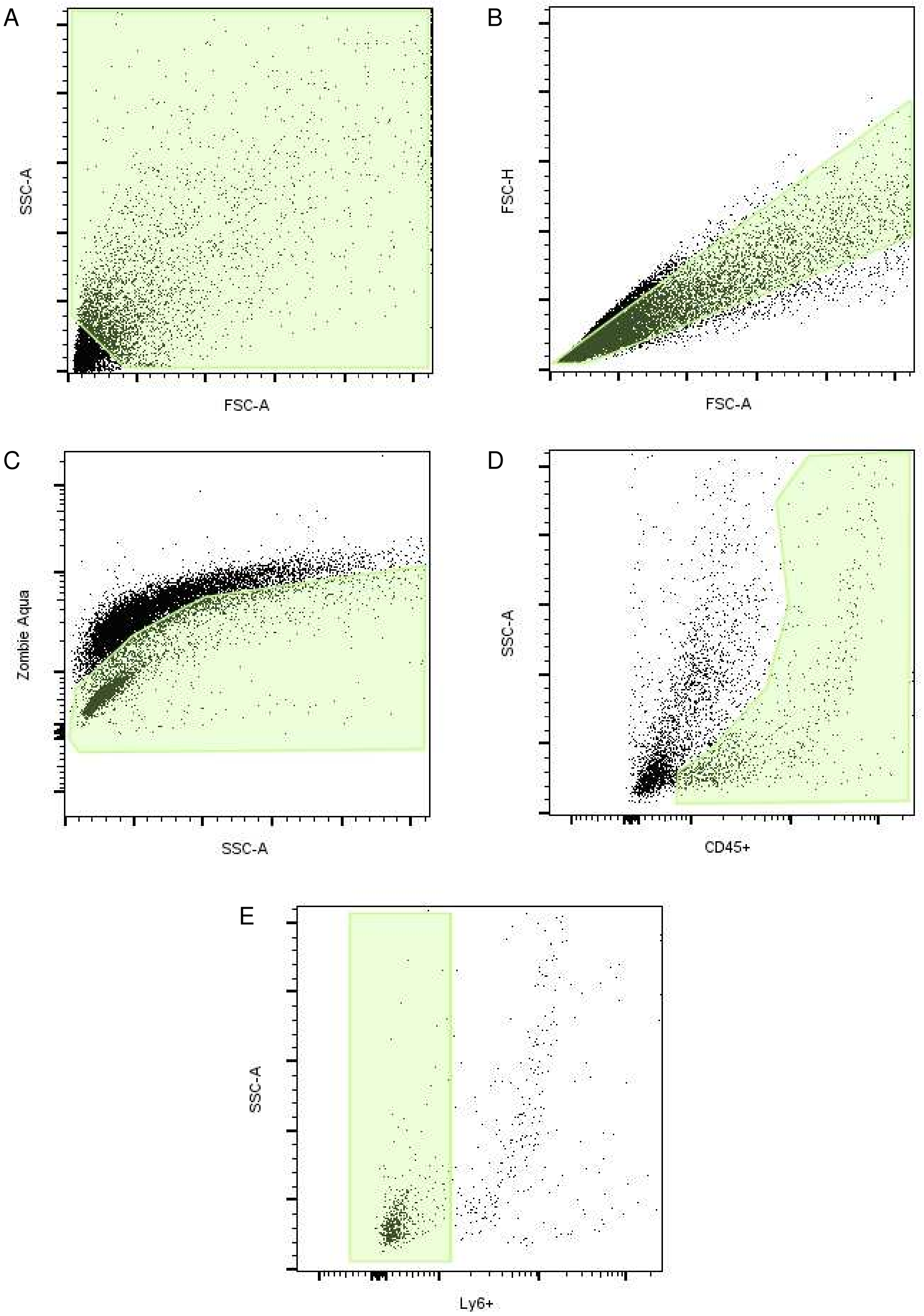

Supplemental Figure 9. Gating strategy relating to Figure 3

Cardiac macrophages were gated using granularity (FSC) (**A**) and size (SSC) (**B**) with the additional exclusion of Zombie Aqua™ stained dead cells (**C**), the inclusion of $CD45^{+}$ stained leukocytes (**D**) and the further exclusion $Ly6^{+}$ stained neutrophils (**E**).

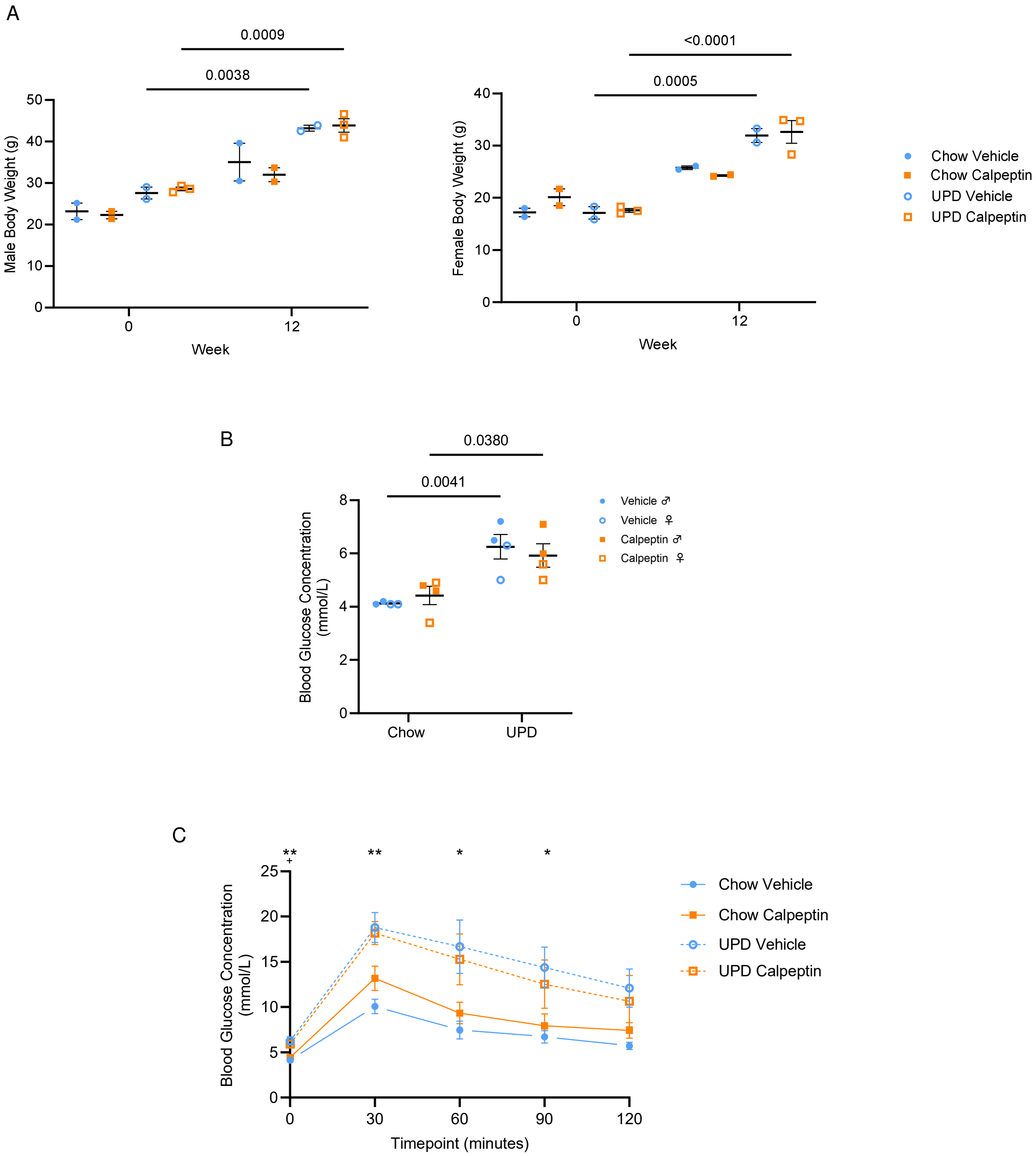

Supplemental Figure 10. Calpeptin administration does not prevent ultra-processed diet (UPD) induced metabolic disorder

A, Male and female body weights at the beginning of the study and following 12 weeks of chow or UPD diet (n=2-3 mice). **B**, Fasting blood glucose, and **C**, glucose tolerance test following 12 weeks of chow or UPD feeding (n=4 mice). Data are presented as mean ±SEM with solid and hollow symbols representing male and female mice respectively (**A-B**). *p* values were calculated using two-way ANOVA with Šidák post-hoc test (**A-C**). Significant differences in blood glucose levels at any timepoint between UPD AAV9-cTnT-e*Gfp* (*) and AAV9-cTnT-*CAST* (+) mice and their respective chow fed cohorts are presented.

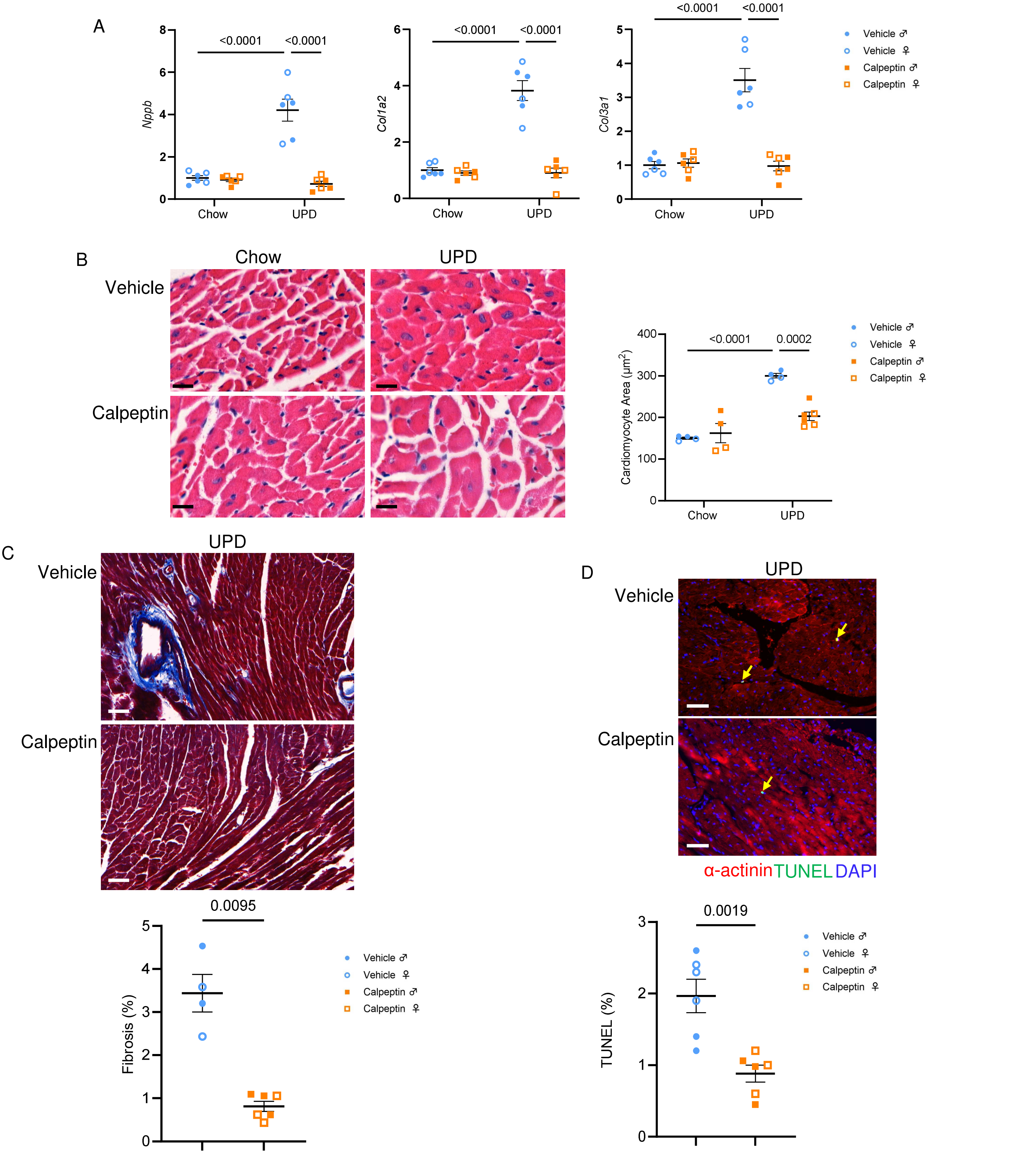

Supplemental Figure 11. Calpeptin administration protects the heart against ultra-processed diet (UPD) induced pathological remodeling

A, Quantitative PCR of genes related to cardiac hypertrophy, *Nppb*, and fibrosis, *Col1a2* and *Col3a1* (n=6 hearts). **B**, Hematoxylin and eosin staining for cross-sectional area (scale bar=50 μm) (n=4-6 hearts). **C**, Masson's Trichrome staining of fibrosis in the heart (scale bar=20 μm) (n=4-6 hearts). **D**, Representative images and quantification of TUNEL staining (scale bar=50 μm, arrows indicate TUNEL-positive cardiomyocytes), DAPI stained nuclei (blue) (n=6 hearts). Data are presented as mean ±SEM with solid and hollow symbols representing male and female mice respectively. *p* values were calculated using 2-tailed Student's t tests (**A, C-D**) and two-way ANOVA with Šidák post-hoc test (**B**).

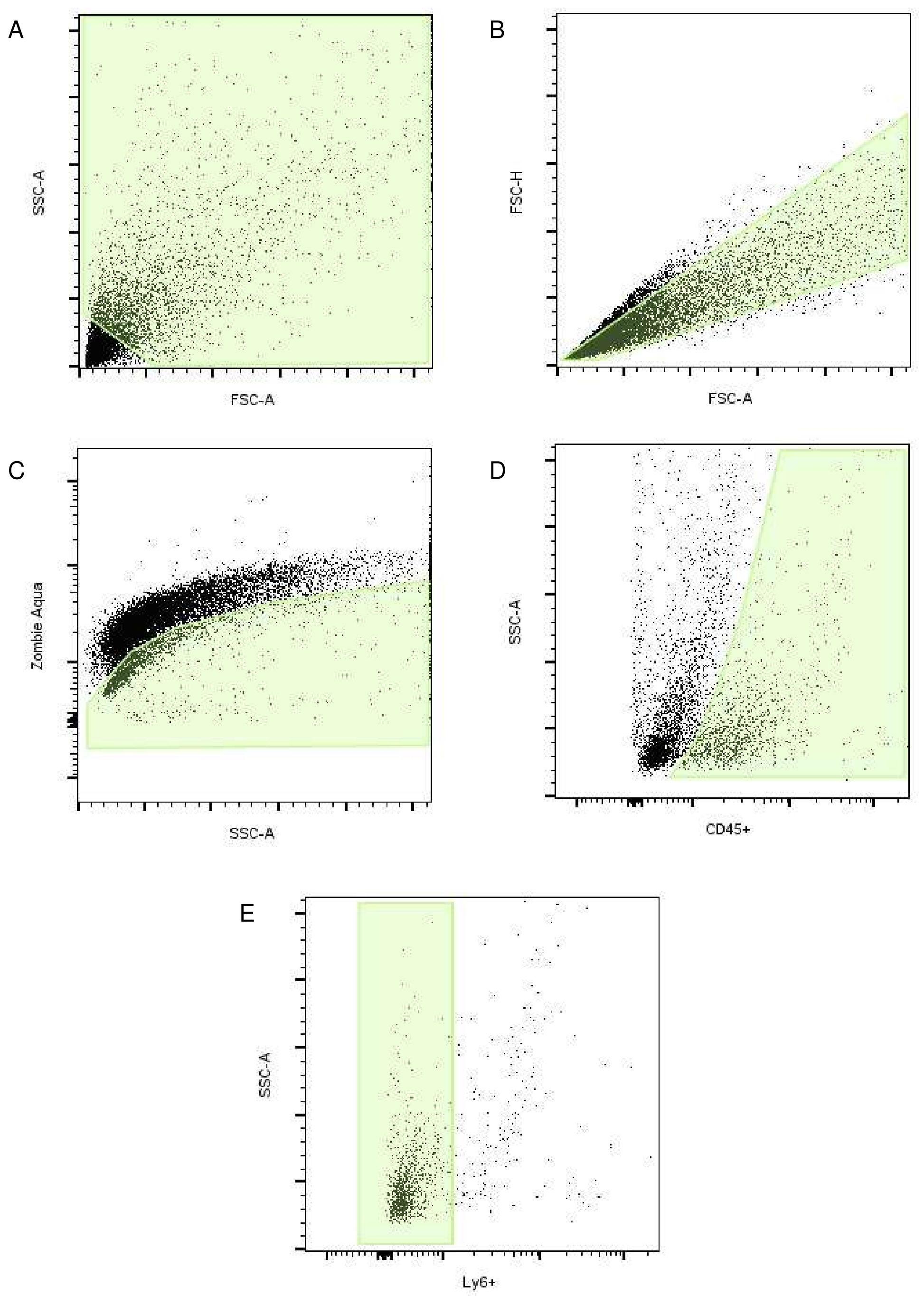

Supplemental Figure 12. Gating strategy relating to Figure 5

Cardiac macrophages were gated using granularity (FSC) (**A**) and size (SSC) (**B**) with the additional exclusion of Zombie Aqua™ stained dead cells (**C**), the inclusion of $CD45^{+}$ stained leukocytes (**D**) and the further exclusion $Ly6^{+}$ stained neutrophils (**E**).

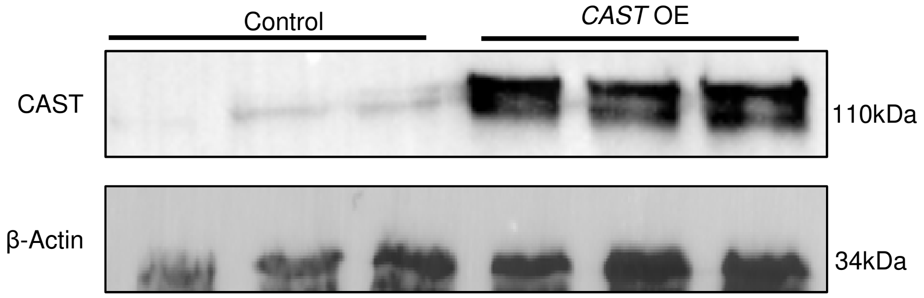

Supplemental Figure 13. Validation of CAST overexpression *in vitro*

Immunoblots validating an increase in CAST expression following transfection of human *CAST* cDNA in neonatal rat cardiomyocytes (NRCMs), where β-Actin is used as a loading control.

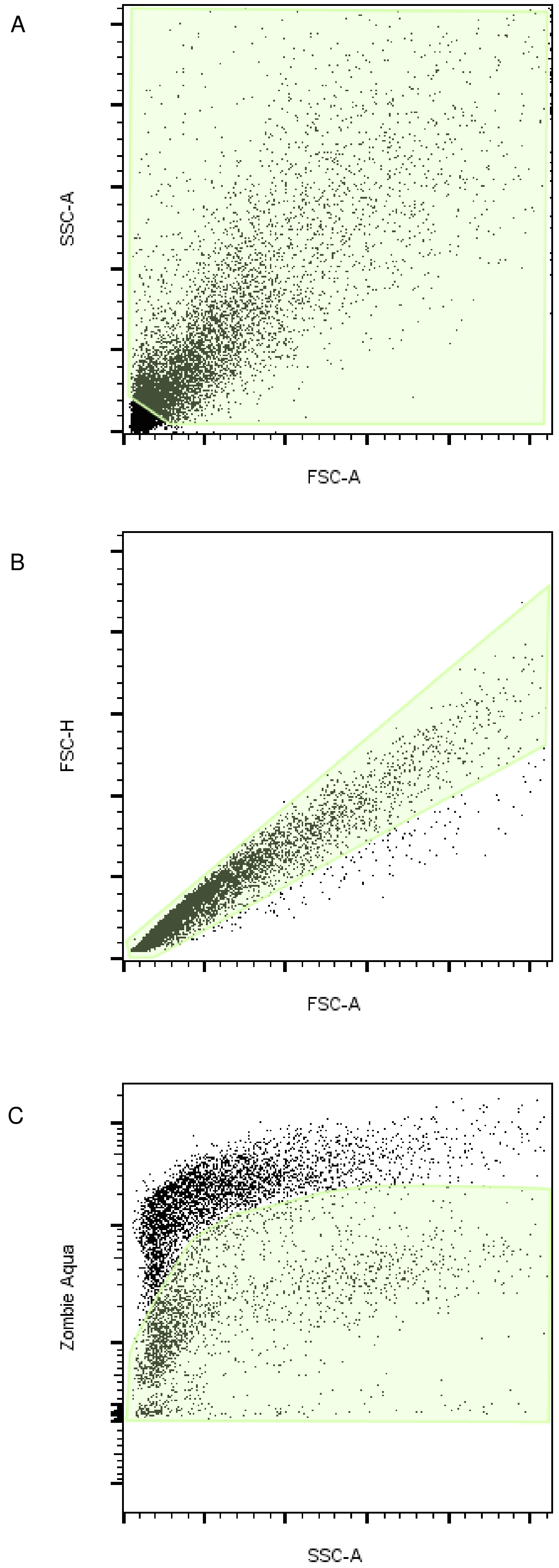

Supplemental Figure 14. Gating strategy relating to Figure 10

THP-1 macrophages were gated using granularity (FSC) (**A**) and size (SSC) (**B**) with the additional exclusion of Zombie Aqua™ stained dead cells (**C**).

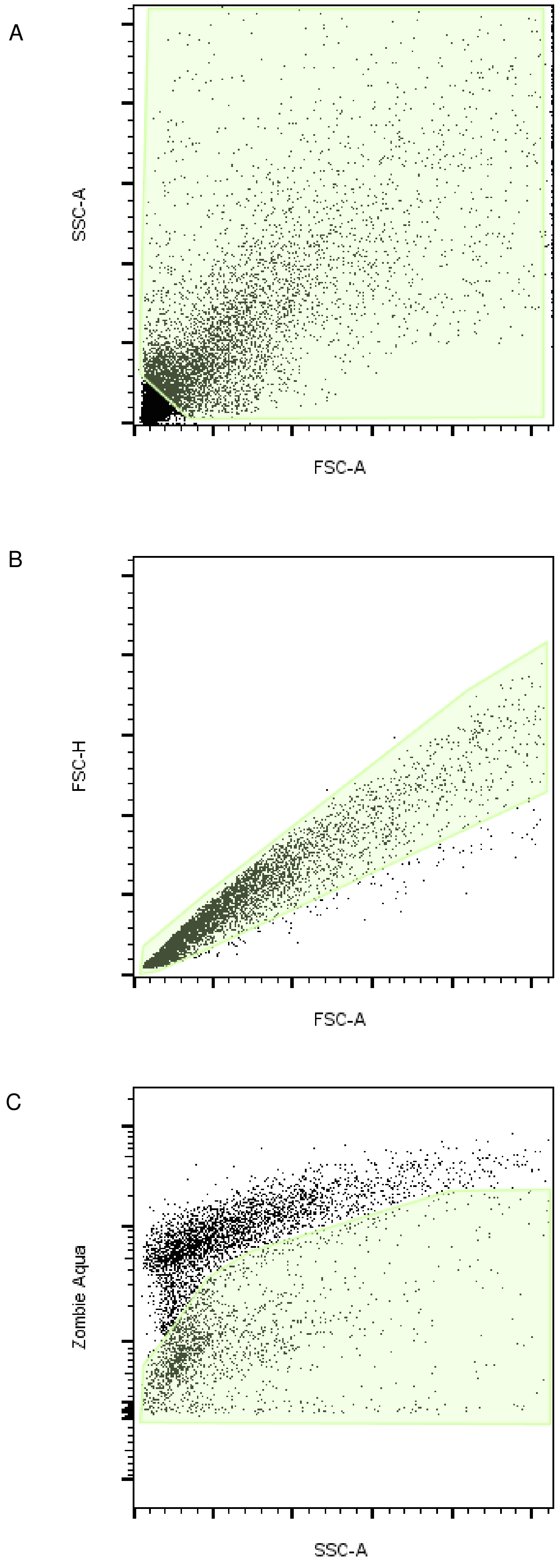

Supplemental Figure 15. Gating strategy relating to Figure 11

THP-1 macrophages were gated using granularity (FSC) (**A**) and size (SSC) (**B**) with the additional exclusion of Zombie Aqua™ stained dead cells (**C**).

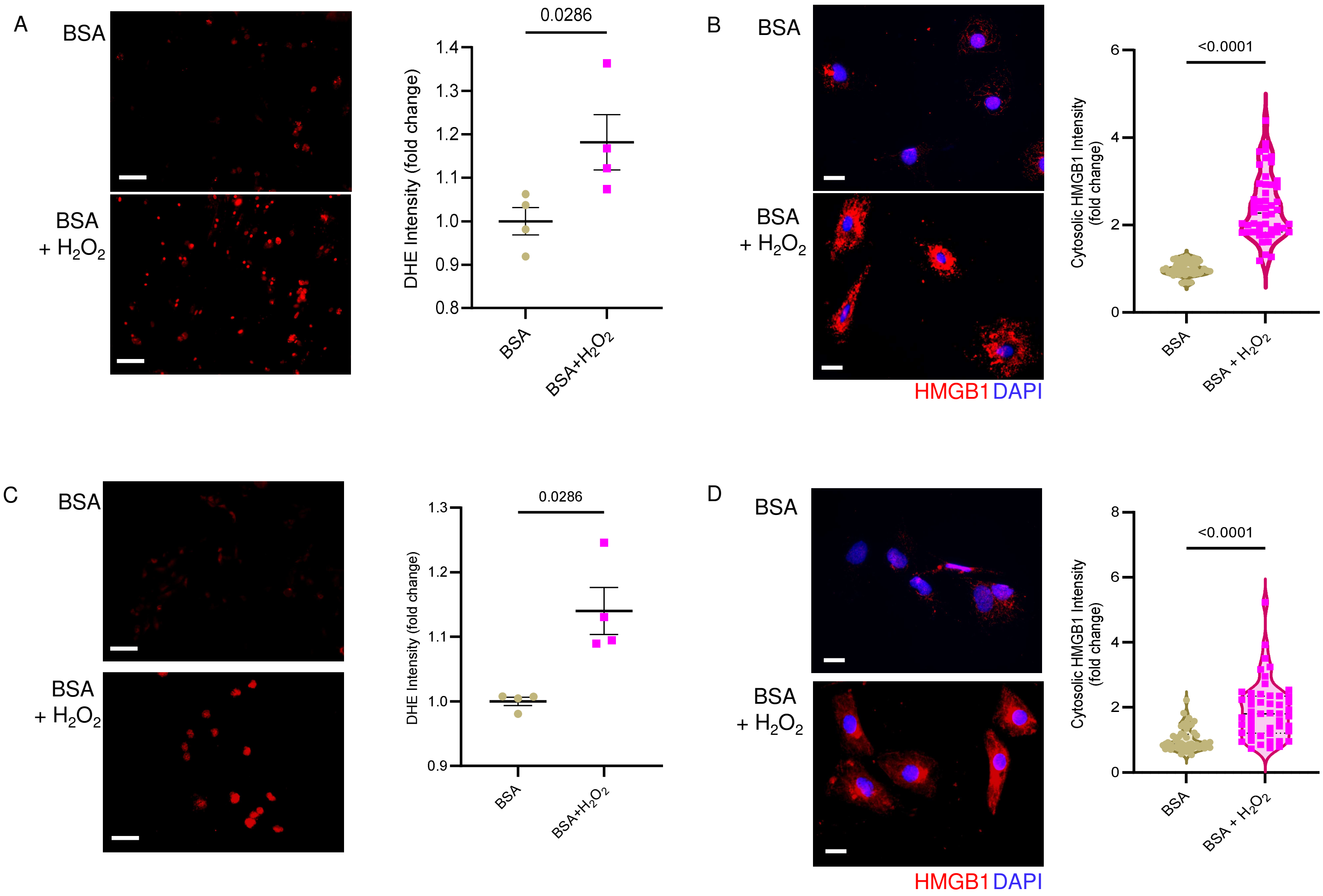

Supplemental Figure 16. Oxidative stress induced increase in cytosolic HMGB1 expression

Representative images and quantification of DHE (scale bar=50 μm) (n=4 experiments) (**A**, **C**) and cytosolic HMGB1 staining (red) with DAPI stained nuclei (blue) (scale bar=20 μm) (n=50 cells across 3 experiments) (**B**, **D**) in NRCMs (**A**-**B**) and hiPSC-CMs (**C**-**D**) for 24 hours with or without H_2O_2 stimulation (200 μM). Data are presented as mean ±SEM. *p* values were calculated using Mann-Whitney test (**A**-**D**).

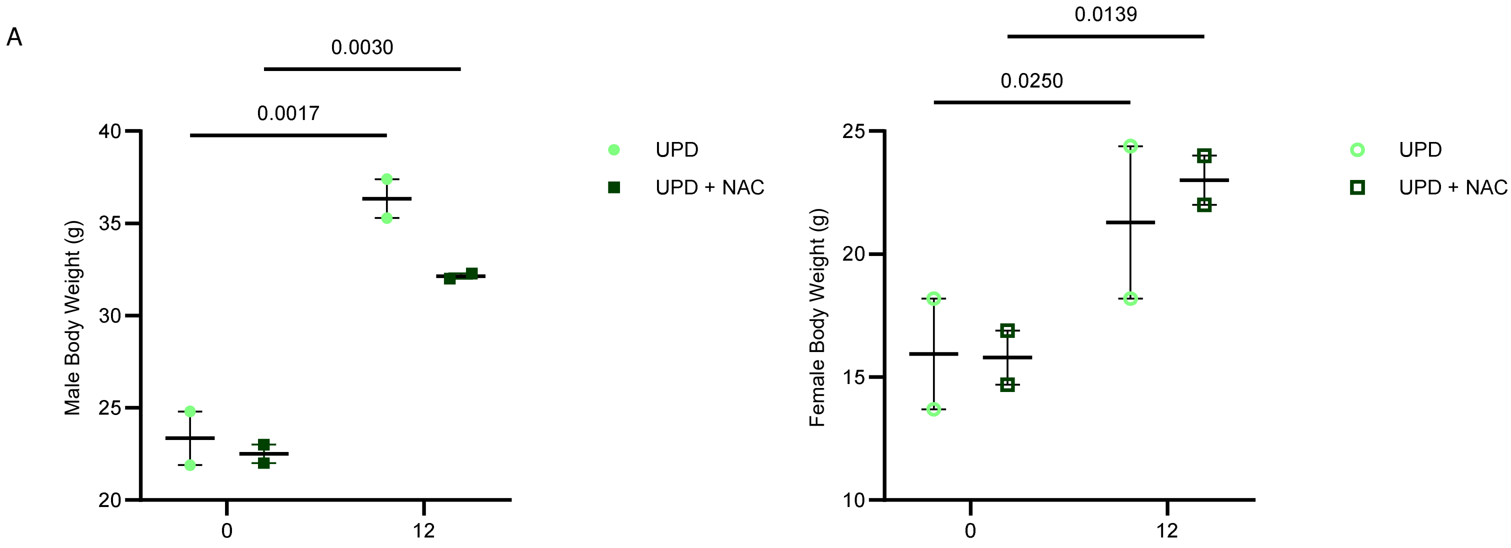

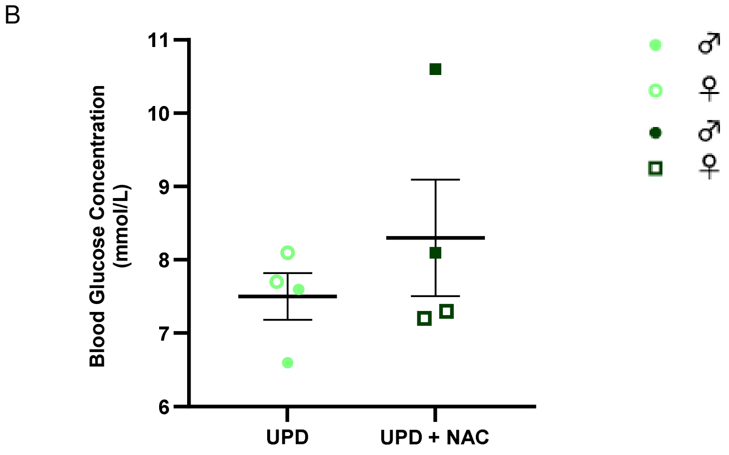

Supplemental Figure 17. NAC administration does not prevent ultra-processed diet (UPD) induced metabolic disorder

A, Male and female body weights at the beginning of the study and following 12 weeks of chow or UPD diet (n=2 male mice / 2 female mice). **B**, Fasting blood glucose following 12 weeks of chow or UPD feeding (n=4 mice). Data are presented as mean ±SEM with solid and hollow symbols representing male and female mice respectively (**A**-**B**). *p* values were calculated using two-way ANOVA with Šidák post-hoc test (**A**) and Mann-Whitney test (**B**).
