# Supplementary material for: Inhibition of calpain-mediated HMGB1 alleviates cardiac inflammation and dysfunction induced by ultra-processed foods
Source: JCI Insight. 2026 Apr 9;11(11):e199622. doi: 10.1172/jci.insight.199622 (PMC13313562; doi:10.1172/jci.insight.199622)

Full unedited gels for figure 2A

Calpain-1

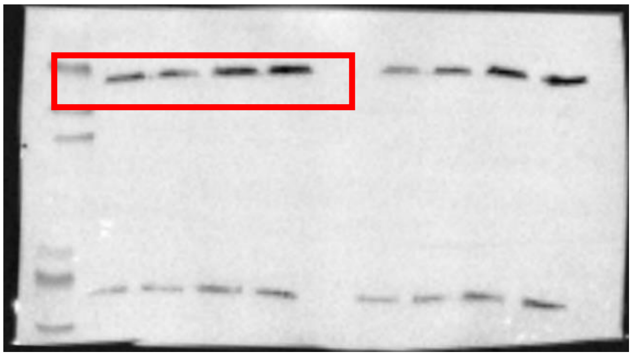

Calpain-2

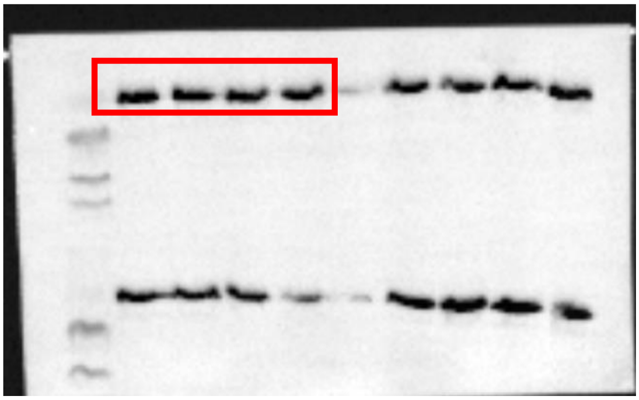

HMGB1

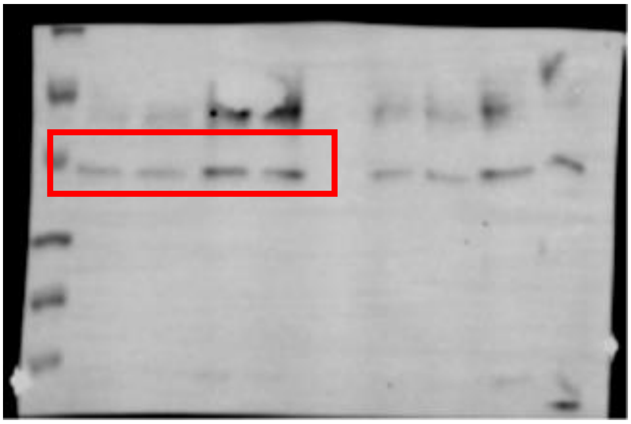

GAPDH

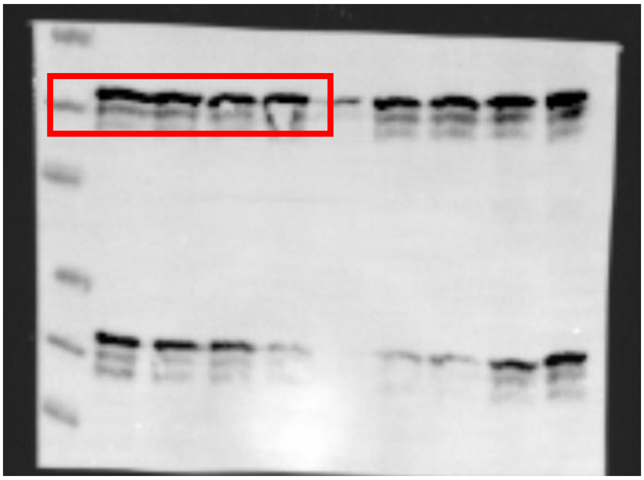

Full unedited gels for figure 2F

Calpain-1

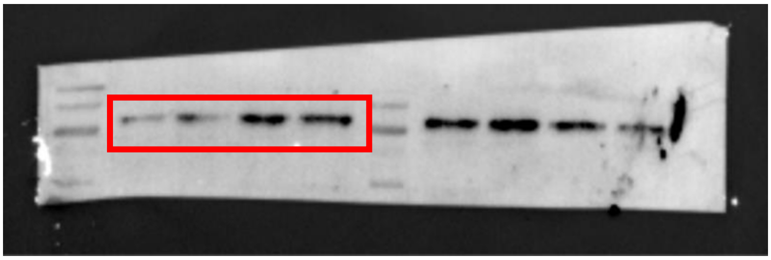

Calpain-2

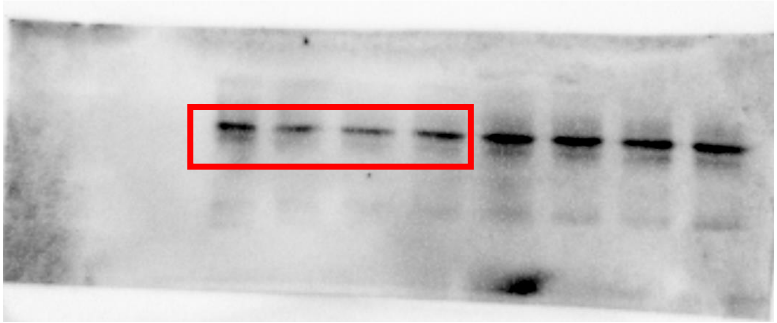

HMGB1

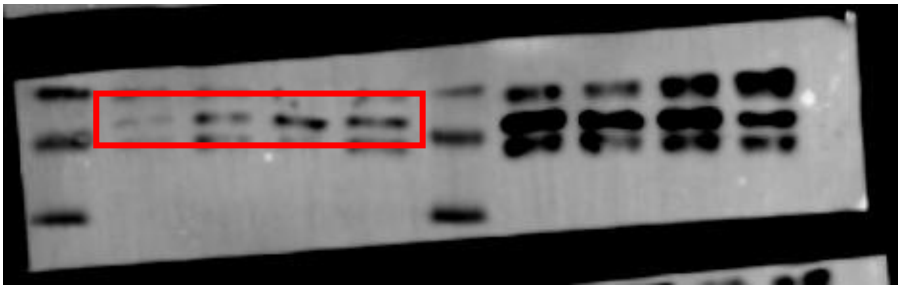

β-Actin

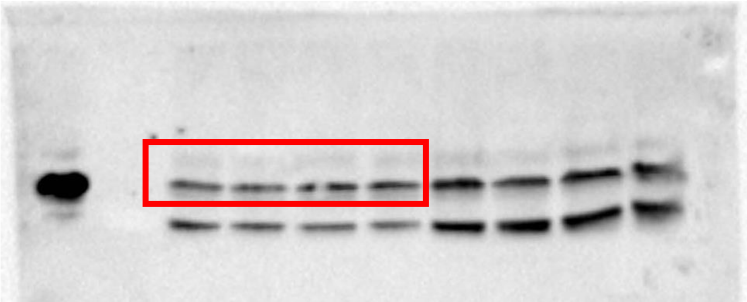

Full unedited gels for figure 5B

HMGB1

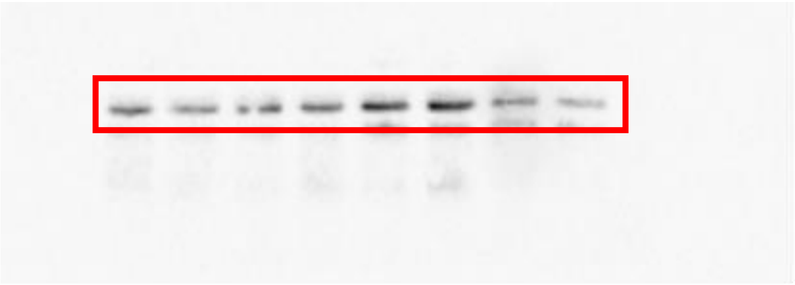

β-Actin

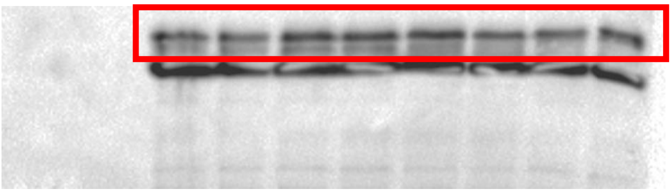

Full unedited gels for figure 8B

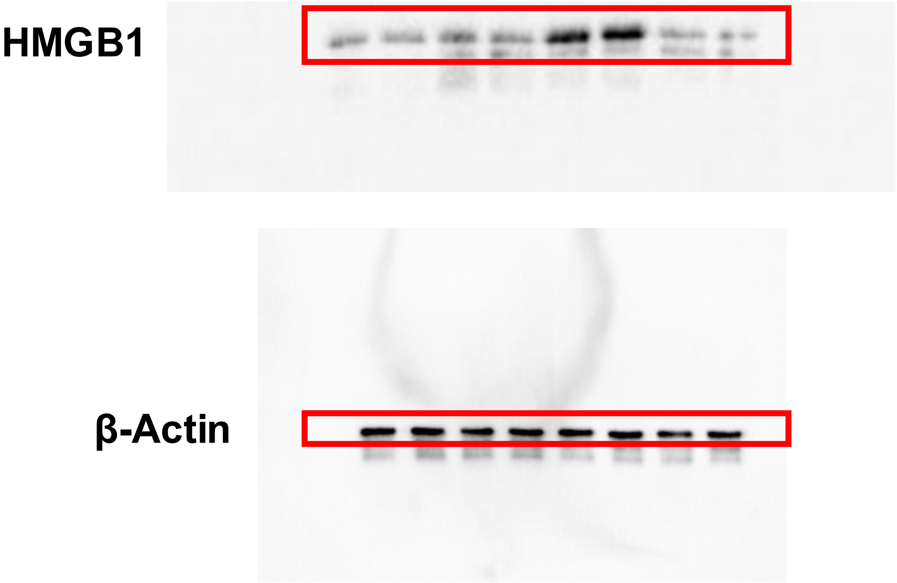

Full unedited gels for figure 9A

HMGB1

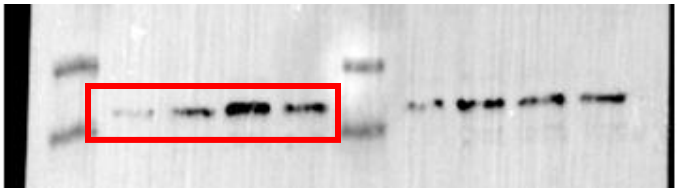

β-Actin

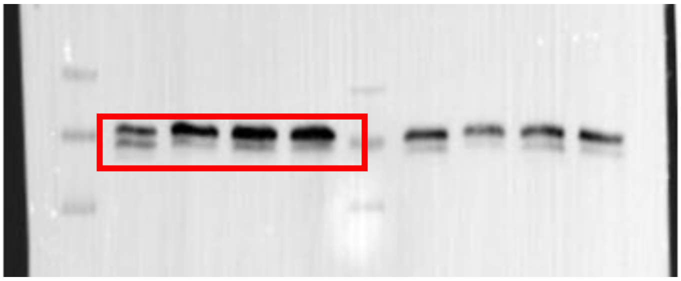

Full unedited gels for figure 9C

HMGB1

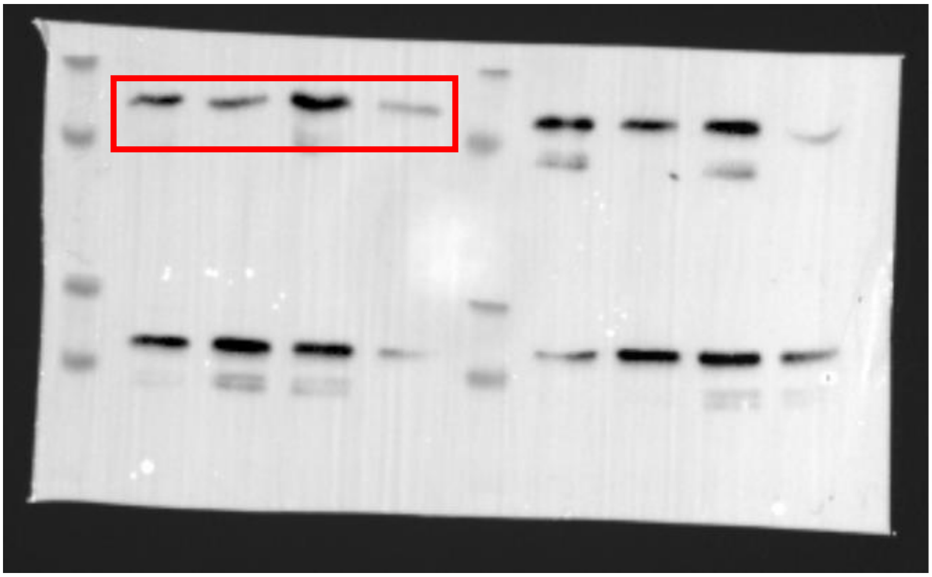

β-Actin

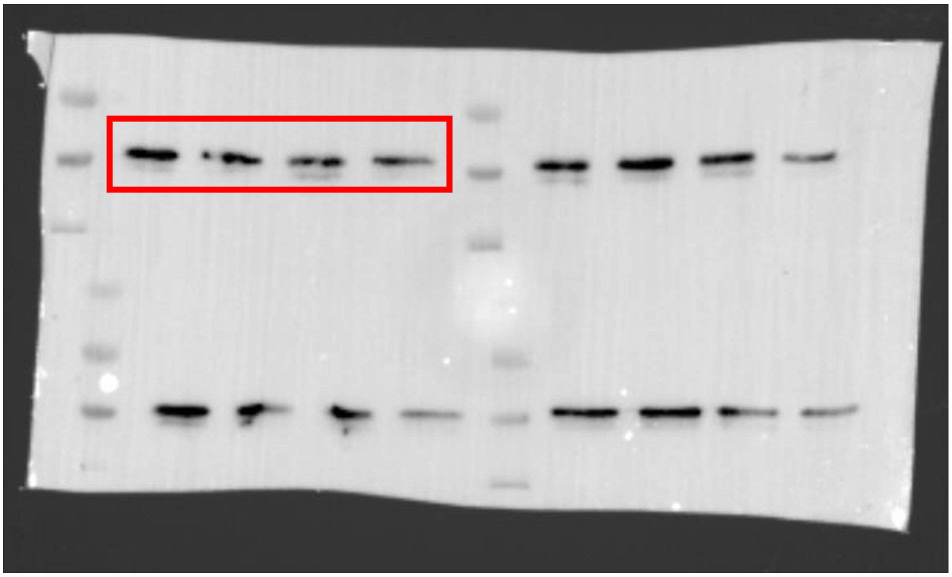

Full unedited gels for figure 11A

HMGB1

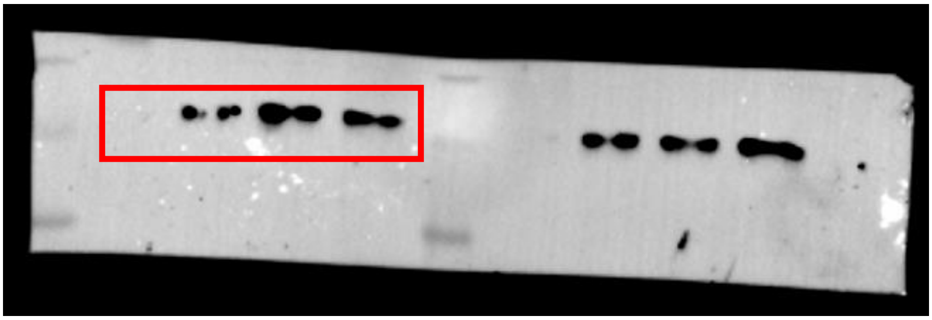

β-Actin

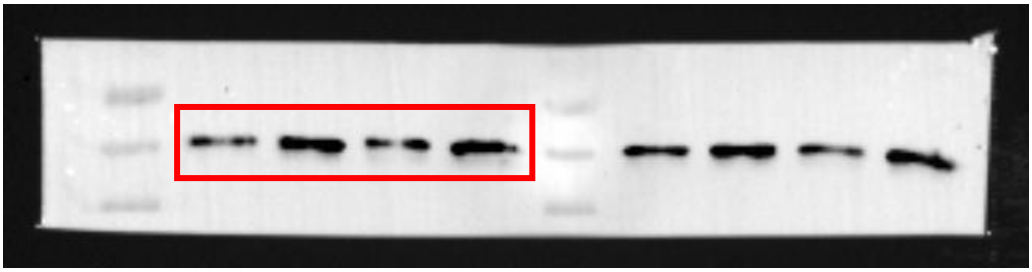

Full unedited gels for figure 13F

HMGB1

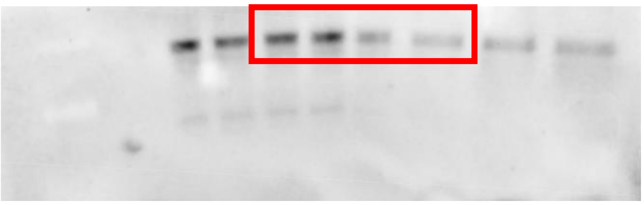

β-Actin

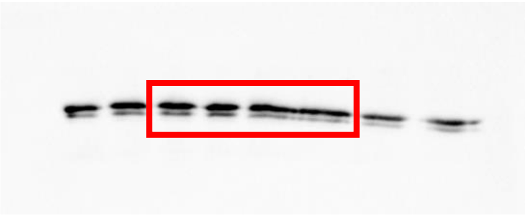

Full unedited gels for supplemental figure 3A

Calpain-1

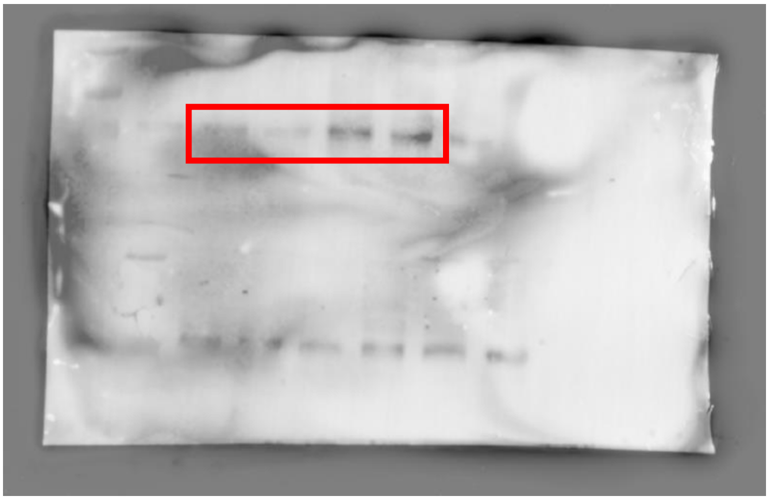

β-Actin

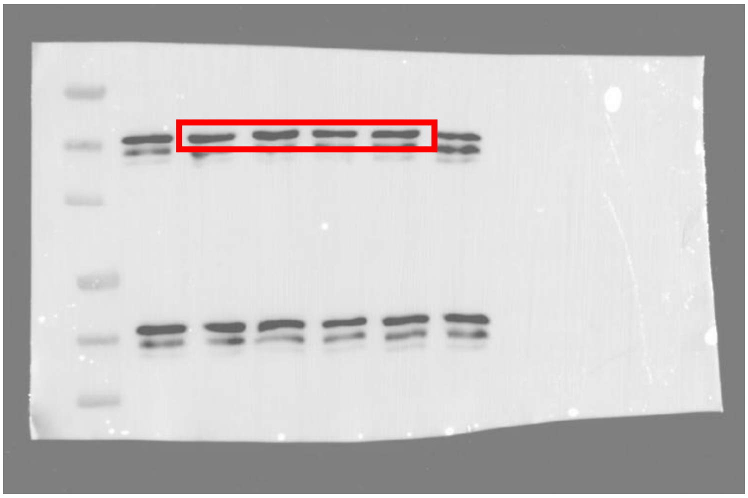

Full unedited gels for supplemental figure 3B

Calpain-1

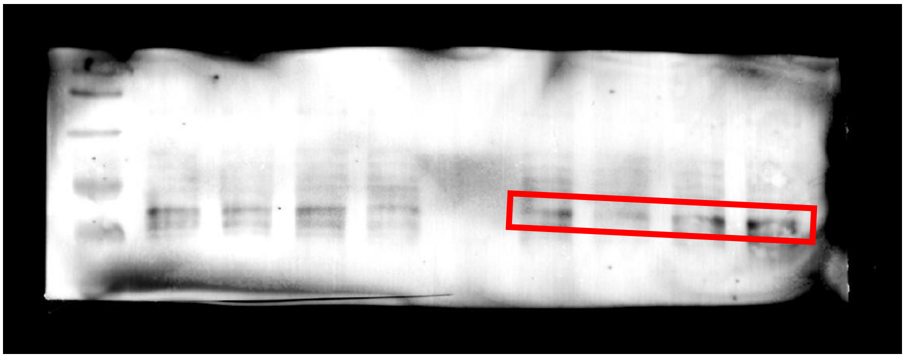

β-Actin

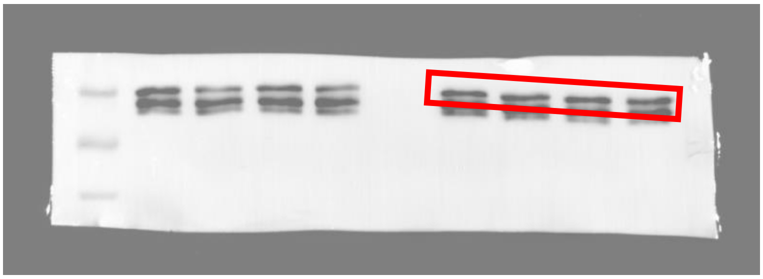

Full unedited gels for supplemental figure 4A

Calpain-1

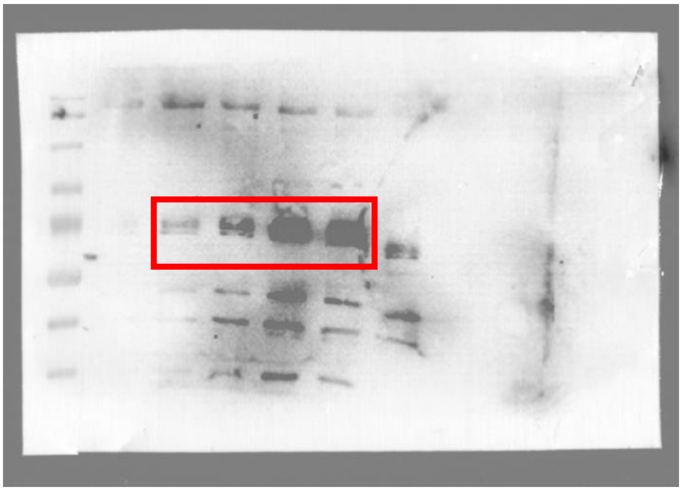

β-Actin

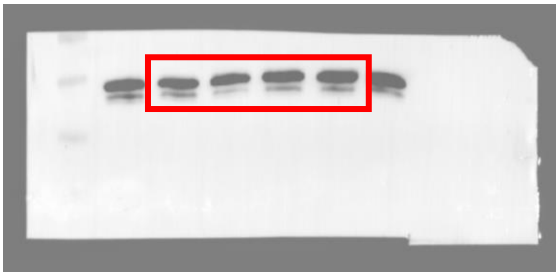

Full unedited gels for supplemental figure 5B

CAST

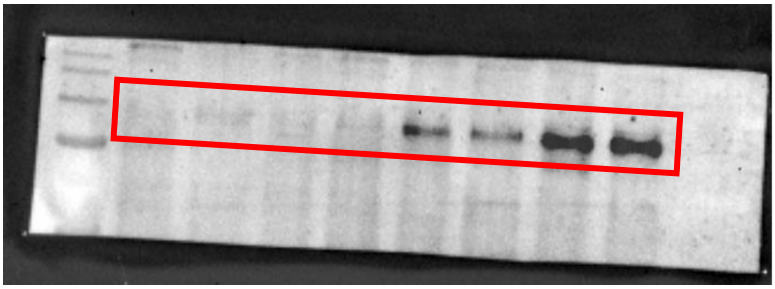

β-Actin

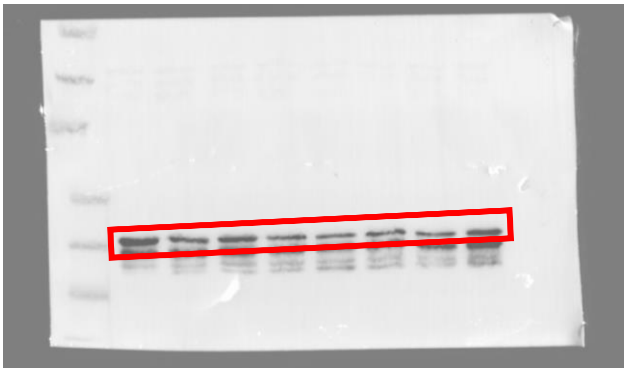

Full unedited gels for supplemental figure 13

CAST

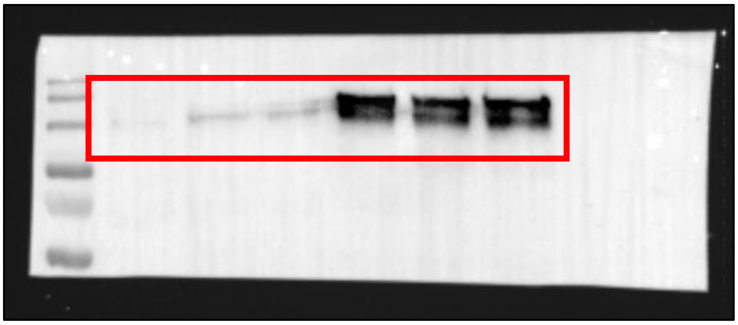

β-Actin

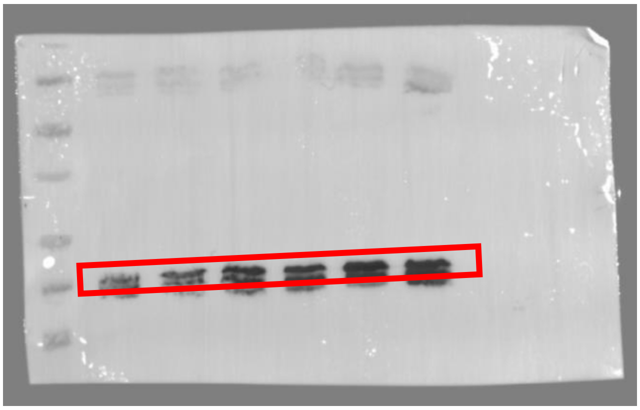

Supplement: Unedited blot and gel images [file jciinsight-11-199622-s133.pdf]
